# Intermolecular Proton-Coupled Electron Transfer Reactivity from a Persistent Charge-Transfer State for Reductive Photoelectrocatalysis

**DOI:** 10.1021/jacs.4c02610

**Published:** 2024-04-26

**Authors:** Pablo Garrido-Barros, Catherine G. Romero, Jay R. Winkler, Jonas C. Peters

**Affiliations:** Division of Chemistry and Chemical Engineering, California Institute of Technology (Caltech), Pasadena, California 91125, United States

## Abstract

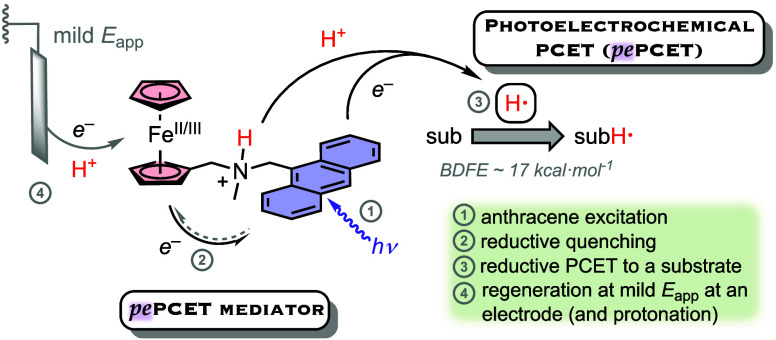

Interest in applying proton-coupled electron transfer (PCET) reagents in reductive electro- and photocatalysis requires strategies that mitigate the competing hydrogen evolution reaction. Photoexcitation of a PCET donor to a charge-separated state (CSS) can produce a powerful H-atom donor capable of being electrochemically recycled at a comparatively anodic potential corresponding to its ground state. However, the challenge is designing a mediator with a sufficiently long-lived excited state for bimolecular reactivity. Here, we describe a powerful ferrocene-derived photoelectrochemical PCET mediator exhibiting an unusually long-lived CSS (τ ∼ 0.9 μs). In addition to detailed photophysical studies, proof-of-concept stoichiometric and catalytic proton-coupled reductive transformations are presented, which illustrate the promise of this approach.

## 1 Introduction

**Figure 1:**
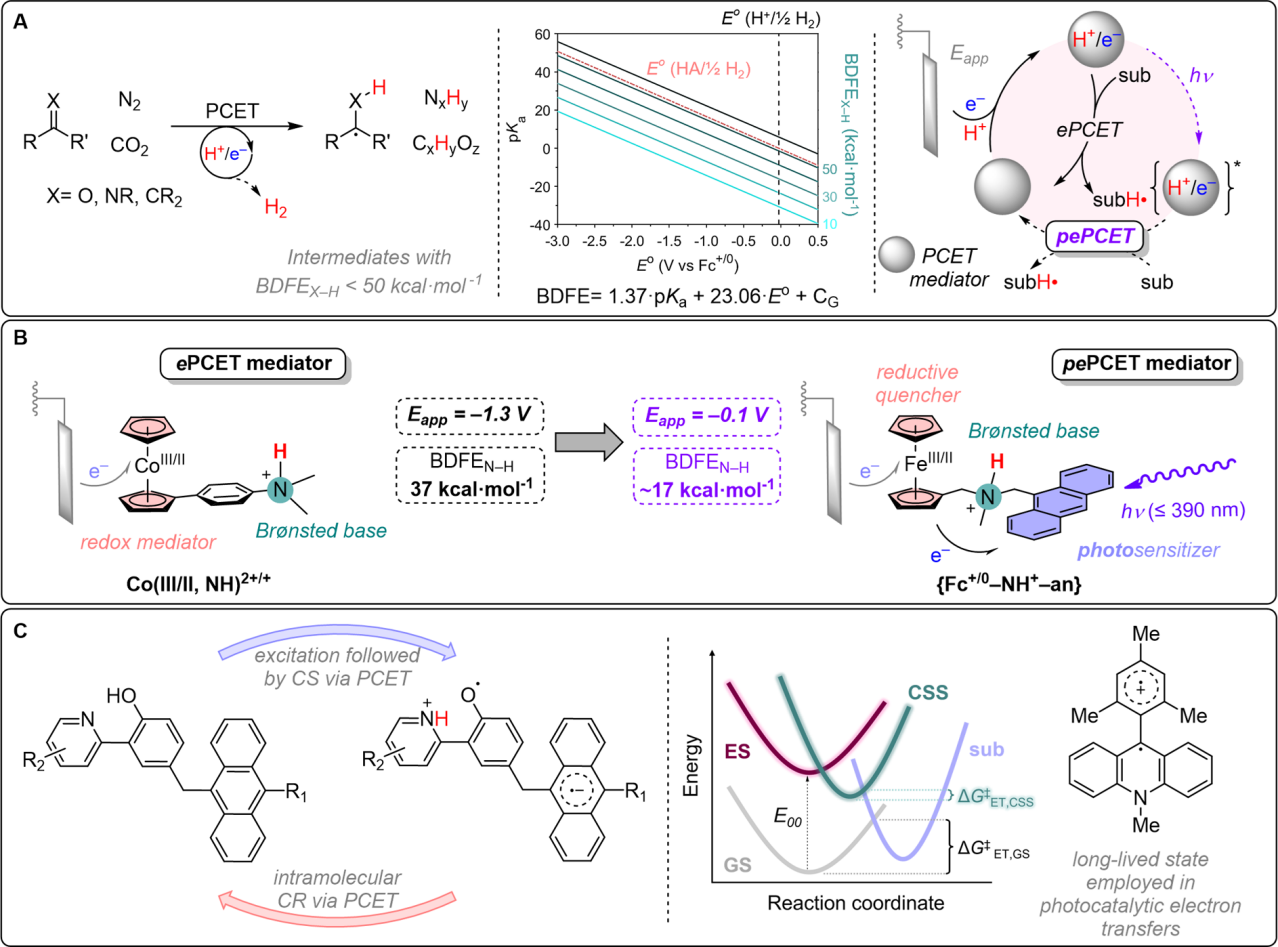
(A) PCET in reductive transformations. Relationship between p*K*_a_ and formal potential for PCET donors with different BDFE values in MeCN (derived using the Bordwell equation in the inset). The formal potential of the H^+^/H_2_ couple is indicated by a dashed black line. C_G_ value in MeCN from ref (13). (B) Previously reported electrocatalytic PCET (*e*PCET) mediator in comparison with this report of a photoelectrocatalytic PCET (*pe*PCET) mediator. (C) Examples of reported systems invoking a charge-separated state (CSS). (CS = charge separation; CR = charge recombination). Simplified energy diagram for ET from a CSS.

Proton/electron transfers to chemical substrates permeate metabolic and synthetic reactions.^1−7^ Proton-coupled electron transfer (PCET) offers a means to bypass high energy pathways associated with stepwise electron/proton transfer (ET/PT) steps.^4^ Yet, such reactions pose a considerable selectivity challenge when strongly reducing conditions are required to generate reactive intermediates featuring weak X–H bonds (bond dissociation free energies, BDFE_X–H_ < 50 kcal mol^–1^; Figure 1A, left). Under such conditions, the hydrogen evolution reaction (HER) is thermodynamically (Figure 1A, middle) and often also kinetically favored (BDFE_H–H_ = 104 kcal mol^–1^). This challenge calls for approaches that disfavor competing HER.^8−12^ The widespread use of stoichiometric SmI_2_/ROH reagents in chemical synthesis underscores this idea.^13−15^

Concomitant with growing interest in electrocatalysis for synthetic organic^16^ as well as solar fuels^17,18^ applications, the challenge of competing HER becomes paramount. Electrode-mediated HER, for example at a commonly used glassy carbon (GC) electrode,^19^ can kinetically dominate at an applied potential (*E*_app_) sufficiently negative to drive a turnover-limiting ET step, irrespective of the inherent selectivity of a soluble chemical catalyst system. To mitigate this issue, our lab recently introduced a strategy wherein a PCET mediator composed of a cobaltocene redox site and an appended dimethylaniline Brønsted base site (Figure 1A, right (inner path), and Figure 1B) colocalizes a highly reactive but kinetically trapped H^+^/e^–^ equivalent (BDFE_N–H_ ∼ 37 kcal mol^–1^). The mediator facilitates single- and multielectron substrate reductions fixed to the *E*_app_ of its redox center. This *E*_app_ is anodic of the electrode-mediated HER window for acids tuned to the mediator’s p*K*_a_ (∼8.6 in MeCN), enabling substrate reductions both in the absence^20−22^ and presence^23,24^ of a tandem cocatalyst; these reductions are not feasible in the absence of the PCET mediator at the same *E*_app_.^12^

The linear relationship between the driving force (ΔBDFE) and the formal potential required to regenerate the reactive form of the PCET mediator can limit its scope. This can be illustrated using a Bordwell analysis,^4,25^ which shows that strong PCET donors (BDFE_X–H_ < 52 kcal mol^–1^) will have formal potentials more negative than the formal potential for HER (*E*°(HA / 1/2 H_2_)) (Figure 1A, middle). An attractive alternative is photochemical generation of highly reactive PCET donors [Figure 1A, right (outer path)].^26,27^ When coupled with acidic or basic functional groups, excited molecules can be powerful PCET reagents.^28^ Recent examples include an anthracene–phenol conjugate (Figure 1C, left)^29^ and a ruthenium tris–diimine complex.^26^ In these systems, intermolecular PCET reactivity is limited by the lifetime of the electronic excited state. For example, in the anthracene–phenol conjugate, the PCET reaction was intramolecular and the <10 ns singlet lifetime of the anthracene component was hence compatible. For bimolecular PCET reactions, however, microsecond survival times are desired for efficient reactivity. Herein, we describe a photochemical system (Figure 1B) that exploits an excitation-quench strategy to extend the lifetime of a powerful PCET reagent that can be electrochemically regenerated at potentials that avoid the HER.

Visible light excitation (*E*_00_ ∼ 2–3 eV) produces transient species that are far more readily reduced than their ground state analogues (eq 1).
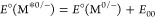
1

Quenching short-lived (exponential decay time τ < 1 μs) excited states with mild reductants (Q) can transiently produce strong PCET donors with highly negative formal potentials (*E*°(M^0/–^)). In our hybrid *pe*PCET approach, the quencher is regenerated at an electrode with an applied potential that is only slightly more negative than *E*°(Q^+/0^). To quench high-energy (*E*_00_ > 3 eV) short-lived singlet excited states of organic molecules (e.g., τ < 20 ns), the electron donor can be covalently coupled to the photosensitizer to force an intramolecular quenching pathway, increasing the relative quantum yield. While permitting rapid excited-state CS, this approach runs the risk of promoting equally rapid and nonproductive charge recombination (CR). The inverted driving-force regime of Marcus theory^30−32^ offers a potential solution to this problem.

We reasoned that if the CR reaction has sufficient driving force and small enough reorganization energy (Figure 1C), the survival time for a CSS might be extended into the microsecond regime, thereby allowing ample time for intermolecular ET or PCET steps. This strategy is the basis of energy storage reactions in photosynthetic reaction centers^31,32^ and has been employed to protract the lifetimes of CS species in photocatalytic ETs (Figure 1C, right).^33,34^

The choice of the hybrid *pe*PCET mediator was motivated by our cobaltocene *e*PCET reagent and features a ferrocene subunit as the reductive quencher and redox mediator appended to an alkylamino Brønsted base and an anthracene photosensitizer (abbreviated herein as **{Fc–NH**^**+**^**–an}** in its iron(II) protonated form; Figure 1B). The synthesis of this complex was originally reported by Farrugia and Magri for the development of a Pourbaix sensor in logic gates.^35^ Near-ultraviolet (390 nm) irradiation of **{Fc–NH**^**+**^**–an}** promotes the anthracene chromophore to its lowest singlet excited state (^**1**^an*, S_1_). This state is quenched by intramolecular ET from Fc, producing **{Fc**^**+**^**–NH**^**+**^**–an**^**•–**^**}** (CSS). If sufficiently long-lived, the powerfully reducing anthracene radical anion, colocalized with a proton on the amine base in the CSS, might be primed for intermolecular PCET reactivity.

## 2 Results and Discussion

**Figure 2:**
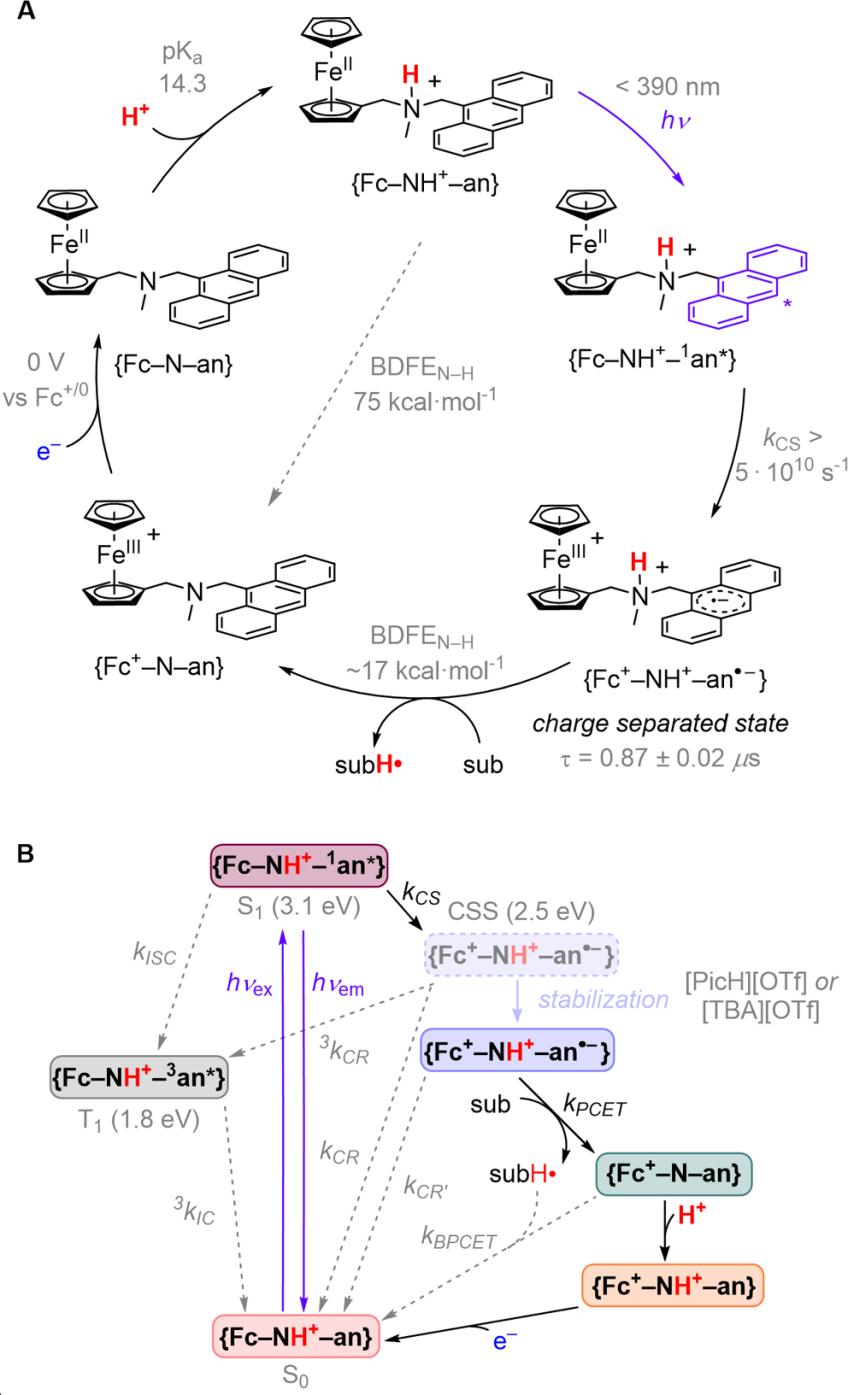
(A) Thermodynamic parameters for the **{Fc–N–an}** system toward ground-state and excited-state PCET, including calculated parameters and measured values. (B) Jablonski representation of the electronic states of **{Fc–N–an}**, indicating a stabilization of the CSS in the presence of a salt. (*k*_BPCET_ = rate of back PCET; *k*_IC_ = rate of internal conversion; *k*_ISC_ = rate of intersystem crossing).

**Figure 3:**
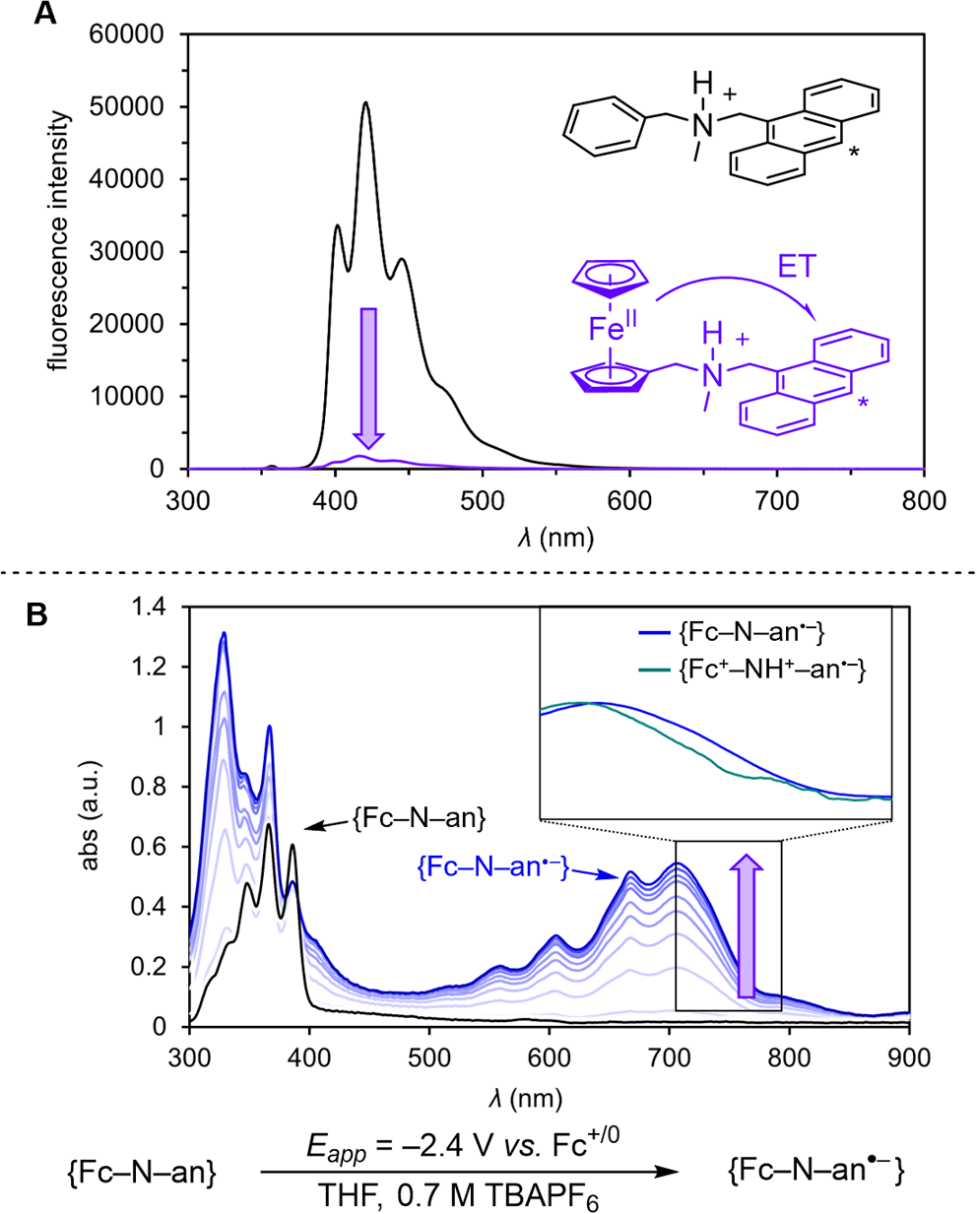
(A) Steady-state emission spectra of **{Fc–NH**^**+**^**–an}** vs **{NH**^**+**^**–an}** in the presence of 15 mM [PicH][OTf] following excitation at 355 nm. (B) Spectroelectrochemistry data for the reduction of **{Fc–N–an}** to **{Fc–N–an**^**•–**^**}** (0.4 mM) in 0.7 M TBAPF_6_ (THF). Inlay includes the TA spectrum of **{Fc**^**+**^**–NH**^**+**^**–an**^**•–**^**}** from 690 to 790 nm. For the steady-state emission data, [**{Fc–N–an}**] = [**{NH**^**+**^**–an}**] = 0.15 mM; [PicH][OTf] = 15 mM in DME. [PicH][OTf] = 2-picolinium triflate.

To guide the following discussion, Figure 2A provides our working model for photoelectrochemical catalysis using **{Fc–NH**^**+**^**–an}** and the pertinent physical parameters. Figure 2B provides a corresponding Jablonski representation of the electronic states of **{Fc–NH**^**+**^**–an}**. Anodic cyclic voltammetry with **{Fc–N–an}** revealed a reversible Fe^III/II^ redox couple [*E*° = 0.00 V vs ferrocenium/ferrocene (Fc^+/0^) in acetonitrile; Fc^+/0^ reference scale used throughout] that shifts to *E*° = 0.13 V upon protonation of the amine group. Cathodic voltammetric sweeps indicate that the anthracene components in **{N–an}** (an organic model derivative without an Fc subunit; see Figure 3A) and **{Fc–N–an}** have similar reduction potentials [*E*°(an^0/•–^) = −2.44 V].^36^ The absorption and fluorescence spectra of the anthracene component in **{Fc–NH**^**+**^**–an}** indicate that *E*_00_ = 3.1 eV, consistent with a potential of *E*°(^1^an^*0/•–^) = 0.7 V in the S_1_ excited state. An NMR titration in deuterated acetonitrile indicates a p*K*_a_ ≈ 14.3 for **{Fc–NH**^**+**^**–an}**. Using this p*K*_a_ value and *E*°(an^0/•–^) for **{Fc–NH**^**+**^**–an}** in a Bordwell analysis, we estimate a very weak BDFE_N–H_ of ≈17 kcal mol^–1^ for **{Fc**^**+**^**–NH**^**+**^**–an**^**•–**^**}** (Figure 2A; *C*_G_ = 52.6 in acetonitrile^3^). This value compares with the ground state BDFE_N–H_ ≈ 75 kcal mol^–1^ in **{Fc–NH**^**+**^**–an}**, where Fc is the source of reducing equivalents (see Supporting Information).

Fluorescence from ^**1**^an* (400–475 nm) in the ET-inactive model complex **{NH**^**+**^**–an}** (Figure 3A) decays with an exponential time constant of 6.45 ± 0.05 ns. Steady state measurements reveal that ^**1**^an* fluorescence from **{Fc–NH**^**+**^**–an}** in the presence of excess acid {picolinium triflate ([PicH][OTf]), 16 mM} is heavily quenched (99%) (Figure 3A), suggesting an excited state lifetime <60 ps. Time-resolved fluorescence measurements reveal a multiexponential decay with the fastest component having a time constant of τ_1_ < 20 ps (see Supporting Information). This lifetime is limited by the response time of our instrument. On the basis of the steady-state spectra it must represent the dominant decay pathway for **{Fc–NH**^**+**^**–**^**1**^an*} when excess acid is present. The rapid fluorescence decay is consistent with rapid ET from Fc to ^**1**^an*, producing **{Fc**^**+**^**–NH**^**+**^**–an**^**•–**^**}** (CSS) with a rate constant of *k*_CS_ > 5 × 10^10^ s^–1^ (−Δ*G*° = 0.6 eV). The slower observed decay components may arise from **{Fc–NH**^**+**^**–**^**1**^an*} conformations that are not suitable for ET, or alternatively a very minor (<1% based on NMR analysis of synthesized material) fluorescent impurity. The ^**1**^an* decay time in **{Fc–NH**^**+**^**–an}** in the absence of excess acid does not exhibit the fastest (<20 ps) decay component and is not as heavily quenched. Instead, the ^**1**^an* decay is biphasic with time constants of 2.5 ± 0.1 (46%) and 11.5 ± 0.2 ns (54%). The faster decay component indicates that CS is much slower in the absence of excess acid. The slower decay component may be a consequence of conformational heterogeneity.

**Figure 4:**
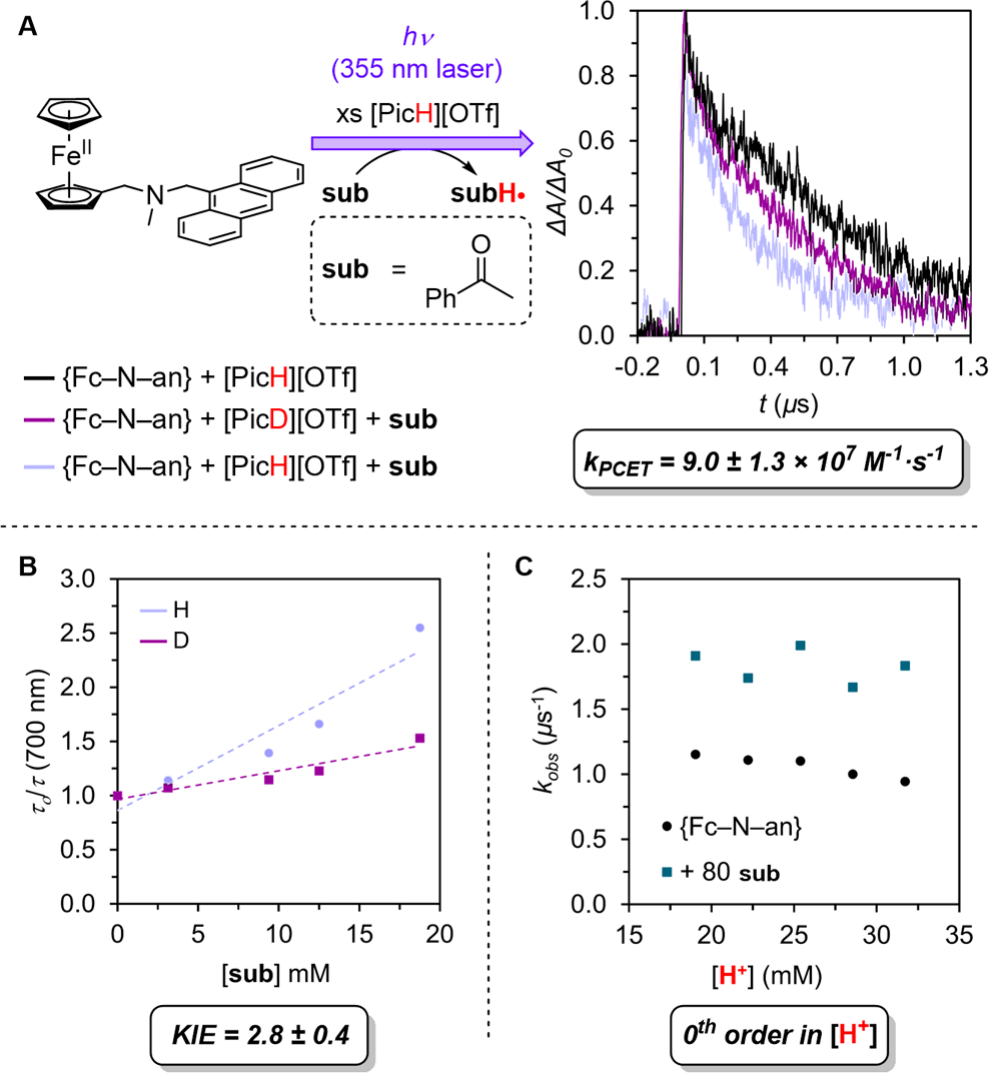
(A) Time-resolved TA decays for **{Fc**^**+**^**–NH**^**+**^**–an**^**•–**^**}** in the presence of excess acid and quencher (acetophenone, **sub**), exciting with a 355 nm laser pulse and monitoring the absorbance at 700 nm after a time-delay of 10 ns. (B) Stern–Volmer quenching plots for the rate of decay of **{Fc**^**+**^**–NH(D)**^**+**^**–an**^**•–**^**}** in the presence of varying concentrations of **sub**, relative to the rate of decay in the absence of **sub**, and an extracted deuterium kinetic isotope effect (KIE). (C) Demonstration of the zero-order dependence of CR and PCET on [H^+^]. For the TA data, [**{Fc–N–an}**] = 0.15 mM; [[PicH(D)][OTf]] = 15 mM; [**sub**] = 18 mM in DME. For the acid-dependence study, [[PicH][OTf]] + [[PicMe][OTf]] = 32 mM.

A transient absorption (TA) spectrum collected after 10 ns laser excitation (355 nm) of **{Fc–NH**^**+**^**–an}** in the presence of excess acid ([PicH][OTf], 15 mM) shows absorbance at 700 nm, consistent with the spectrum of the anthracene radical anion measured spectroelectrochemically (Figure 3B) and hence assignable to **{Fc**^**+**^**–NH**^**+**^**–an**^**•–**^**}** (CSS). TA kinetics monitored at 700 nm further reveal a relatively long-lived species (τ ∼ 0.9 μs; Figure 4A, black trace). The signal has greater amplitude and is somewhat longer-lived (∼0.9 vs 0.6 μs) when excess [PicH][OTf] is present. When [TBA][OTf] (15 mM) is employed as an electrolyte with isolated **{Fc–NH**^**+**^**–an}**, the same species is observed, and its lifetime increases to 1.3 μs, consistent with increased stabilization of the CSS in a higher dielectric environment (Figure 2B). Rate constants for excited-state charge shift and thermal back transfer reactions in **{Fc–NH**^**+**^**–an}** display an inverted driving-force behavior. The rapid excited-state charge shift reaction at low driving force (0.6 eV) implies a modest ET reorganization barrier. Back ET from **{Fc**^**+**^**–NH**^**+**^**–an**^**•–**^**}** to regenerate ground-state **{Fc–NH**^**+**^**–an}** is likely disfavored by the Marcus inverted effect, owing to the high reaction driving force (2.5 eV) and closed shell products.^37^ Conformational dynamics and electronic (spin) barriers might also modulate the observed ET rate constants, warranting future studies to probe them further. The slower CR at a lower driving force is presumably the result of an increase in the reorganizational energy in the higher dielectric medium. A different 420 nm TA feature observed after 10 ns excitation of **{NH**^**+**^**–an}** (τ = 27.8 μs) or **{Fc–NH**^**+**^**–an}** (τ = 2.1 μs) is also present and is attributable to the ^**3**^an* state.^38^ The Jablonski diagram (Figure 2B) illustrates the complex array of radiative and nonradiative pathways available in **{Fc–NH**^**+**^**–an}**.

An alternative assignment for the 700 nm transient would be a species resulting from protonation of the anthracene radical anion by acid ([PicH][OTf], 15 mM) to produce the neutral radical instead. Experimental precedent suggests that protonation at C_9_ by [PicH][OTf] would lead to a neutral radical fragment with absorbance below 500 nm,^38^ in contrast with the observed 700 nm absorbance. Moreover, in an isotope scrambling experiment, where a DME solution of **{Fc–N–an}** and [PicD][OTf] was irradiated at 390 nm for 1 h, ^2^H NMR spectra show no indication of deuterium incorporation into the anthracene moiety (see Supporting Information).

**Figure 5:**
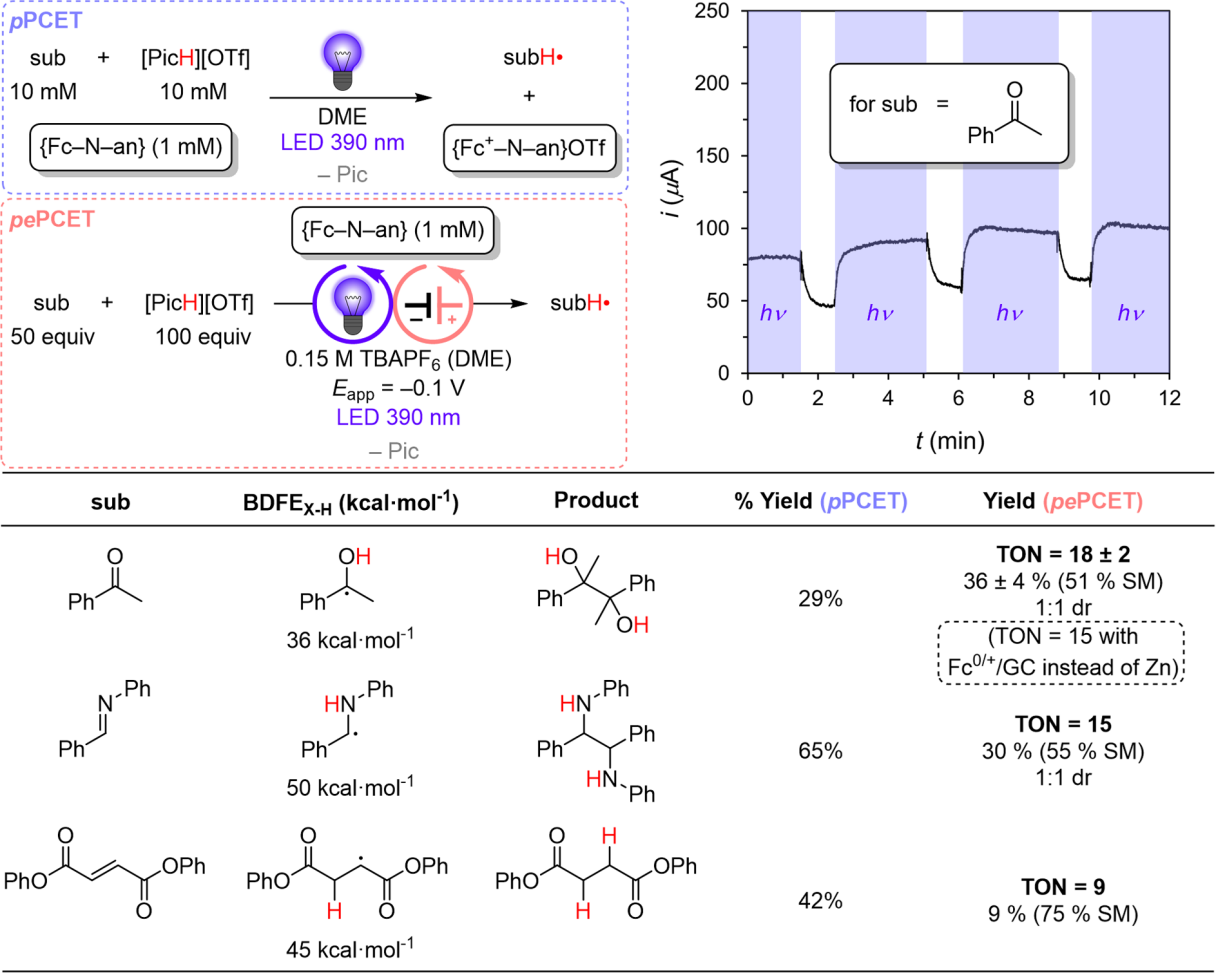
Scope of organic substrates amenable to photochemical reduction via *p*PCET using **{Fc–NH**^**+**^**–an}**. Photoelectrocatalytic reduction of organic substrates by **{Fc–NH**^**+**^**–an}** in the presence of [PicH][OTf] under irradiation at 390 nm and an *E*_app_ of −0.1 V vs Fc^+/0^ using a carbon cloth cathode and either a Zn or GC anode. All reported yields are NMR yields measured against an internal standard (see Supporting Information). Only trace H_2_ (<2% Faradaic efficiency) was detected when **sub** = acetophenone. Calculated BDFE values of key organic intermediates are reported as well.^41^ The inlayed plot is measured current response in the presence and absence of irradiation for the photoelectrocatalytic reduction of acetophenone by **{Fc–NH**^**+**^**–an}**. (SM = starting material).

We next tested whether photochemically generated **{Fc**^**+**^**–NH**^**+**^**–an**^**•–**^**}** could undergo intermolecular PCET reactions, using acetophenone (estimated BDFE_O–H_ = 36 kcal mol^–1^ based on DFT calculations; see Supporting Information)^20^ as an initial test substrate. **{Fc–N–an}** (1 mM) in the presence of [PicH][OTf] (10 mM, p*K*_a_(MeCN) = 13.3)^39^ does not react with acetophenone (10 mM) in the absence of light excitation, owing to the large unfavorable BDFE_X–H_ mismatch. An analogous experiment with 390 nm irradiation, however, afforded 29% of the pinacol-coupled product expected from net H atom transfer to acetophenone and subsequent coupling of the α-ketyl radical intermediates (Figure 5). A photochemical quantum yield of 6% was measured under these conditions (see Supporting Information). Control experiments using **{Fc–NMe**^**+**^**–an}** in the presence of [PicH][OTf], or **{Fc–N–an}** and a weaker acid incapable of protonating the amine base (*p*-CF_3_-benzoic acid), did not produce the (<1%) pinacol product. A binary mixture composed of just the organic fragment **{N–an}** (1 mM) and ferrocene (10 mM) with 10 mM [PicH][OTf] also failed to generate the product, likely owing to the short lifetime of the anthracene singlet excited state and the low energy of the longer-lived anthracene triplet excited state [*E*°(^3^an*^0/•–^) ≈ −0.6 V].^40^ Interestingly, despite the large driving force toward HER, no H_2_ was detected upon irradiation in the absence of the substrate (see Supporting Information). Among the factors that may preclude such reactivity are an unfavorable bimolecular reaction between two cationic species, either **{Fc–NH**^**+**^**–an}** or **{Fc**^**+**^**–NH**^**+**^**–an**^**•–**^**}**, and the statistical improbability of the needed collision between two CSS molecules, given the CSS lifetime.

In the presence of excess [PicH][OTf], transient spectroscopy reveals that the charge-separated intermediate **{Fc**^**+**^**–NH**^**+**^**–an**^**•–**^**}** reacts rapidly with acetophenone (Figure 4A). A plot of the transient decay rate constant versus quencher concentration is linear, consistent with a second-order rate constant ^H^*k*_PCET_ = 9.0 ± 1.3 × 10^7^ M^–1^ s^–1^ (Figure 4B). The second-order rate constant for reaction of **{Fc**^**+**^**–NH**^**+**^**–an**^**•–**^**}** with acetophenone in the presence of [PicD][OTf] (15 mM) is ^D^*k*_PCET_ = 3.2 ± 0.5 × 10^7^ M^–1^ s^–1^ (Figure 4B); the ^H^*k*_PCET_/^D^*k*_PCET_ KIE = 2.8 ± 0.4.

Because an H-bonding interaction has been proposed between acetophenone substrates and phosphoric acids of similar p*K*_a_ to [PicH][OTf] by Knowles,^42^ we explored a similar mechanism in our studies, where [PicH][OTf] would form an H-bonded complex with acetophenone followed by ET from **{Fc**^**+**^**–NH**^**+**^**–an**^**•–**^**}**. We postulated that if this mechanism was operative in the present system, we should see a dependence of the rate of PCET on both [H^+^] and [acetophenone]. To keep the dielectric of the medium relatively constant while varying [[PicH][OTf]], we employed the methylated salt [PicMe][OTf] to maintain the total concentration of ions throughout; [PicMe][OTf] similarly stabilizes the CSS (see Supporting Information). While holding [acetophenone] constant, varying [[PicH][OTf]] shows zero-order dependence (Figure 4C), suggesting that PCET from **{Fc**^**+**^**–NH**^**+**^**–an**^**•–**^**}** to acetophenone does not proceed via H-bonded preassociation with [PicH][OTf]. The KIE value of 2.8 and the lack of dependence on [PicH^+^] is consistent with concerted PT and ET to acetophenone from **{Fc**^**+**^**–NH**^**+**^**–an**^**•–**^**}**.^20^ We also explored the reactivity of **{Fc–NH**^**+**^**–an}** with *N*-phenylbenzylimine and diphenylfumarate (Figure 5). Reaction with *N*-phenylbenzylimine, featuring a C=N π-bond and an associated BDFE_N–H_ for its corresponding iminyl radical calculated to be 50 kcal mol^–1^ (Figure 5), afforded 65% of the aza-pinacol coupling product. Using diphenylfumarate as the substrate (C=C π-bond; BDFE_C–H_ for the succinyl radical calculated to be 45 kcal mol^–1^) afforded 42% of the fully reduced succinate product.^41^

To test the photochemical PCET mediator on an inorganic substrate we turned to the hydrazido complex [(TfO)**W**(NNH_2_)][OTf] [**W** = (dppe)_2_W; OTf = triflate]; we had recently reported its thermal reactivity with a cobalt PCET mediator toward N–N cleavage.^23^ Gratifyingly, irradiation of [(TfO)**W**(NNH_2_)][OTf] in the presence of [PicH][OTf] and **{Fc–N–an}** afforded ∼70% yield of the [(TfO)**W**(NH)][OTf] imido product (evidenced by ^31^P NMR spectroscopy; eq 2). When the ^15^N-labeled complex [(TfO)**W**(^15^N^15^NH_2_)][OTf] was used instead, ^15^NH_4_OTf was detected via ^1^H–^15^N heteronuclear multiple bond correlation (HMBC) NMR spectroscopy. Observation of ^15^NH_4_OTf and also [(TfO)**W**(^15^NH)][OTf] (via ^31^P NMR) are consistent with photoinduced PCET concomitant with N–N bond cleavage (see Supporting Information, Section S14). No reaction occurred in the absence of irradiation or in the absence of **{Fc–N–an}**. However, when **{N–an}** and ferrocene were used in place of **{Fc–N–an}**, appreciable [(TfO)**W**(NH)][OTf] was generated (35% yield), in contrast to a related control experiment for the photochemical reduction of acetophenone, where none of the reduced product is detected (see Supporting Information). We suspect that this result reflects reactivity between the triplet excited state of **{NH**^**+**^**-an}** and [(TfO)**W**(NNH_2_)][OTf].
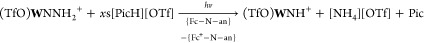
2

We then tested the efficacy of **{Fc–NH**^**+**^**–an}** under photoelectrocatalytic conditions using a high surface area carbon cloth cathode held at a constant *E*_app_ relative to the **{Fc**^**+/0**^**–N–an}** wave. With acetophenone as a test substrate (50 mM), a controlled potential electrolysis, using a divided cell with a DME solution containing 0.15 M TBAPF_6_, 100 mM [PicH][OTf] and 1 mM **{Fc–N–an}**, afforded 36 ± 4% (TON = 18 ± 2) of the pinacol coupled product after 24 h at an *E*_app_ of −0.1 V under 390 nm irradiation (Figure 5). Accounting for the remaining starting material (SM) provided a mass balance of 87%. For comparison, electrocatalytic turnover to the same pinacol product using the previously reported **Co(II, NH)**^**+**^ mediator required an *E*_app_ of −1.3 V.^20^ The photoactive appendage in the ferrocene-derived mediator enables an *E*_app_ that is positively shifted by more than 1 V. The effect of photoirradiation on the electroreduction can be gleaned via light on/off cycles (Figure 5); on removing light, the reductive current decreases as **{Fc**^**+**^**–NH**^**+**^**–an}** is depleted from the surface of the electrode. When irradiation resumes, the current increases as **{Fc**^**+**^**–NH**^**+**^**–an}** is regenerated via PCET to acetophenone.

Photoelectrochemical reduction of *N*-phenylbenzylimine (Figure 5) parallels these results, with the corresponding coupling product obtained in 30% yield (TON = 15), and an 85% mass balance accounting for the remaining SM. In the case of diphenylfumarate, the fully hydrogenated succinate product is obtained in only 9% yield (TON = 9), with a mass balance of 84%. By comparison, diphenylfumarate reduction is more favorably mediated by the cobaltocene-derived mediator under electrochemical conditions.^21^ In the latter case, reduction of the succinyl radical intermediate by the electrode (*E*_app_ = −1.3 V) enabled the net two-electron process. For *pe*PCET with **{Fc–N–an}**, reduction of the radical intermediate (*E*_red_ = −0.7 V) is not feasible. Interestingly, since some reduction still occurs for the case of **{Fc–N–an}**, a multiproton/electron process is apparently feasible via *pe*PCET.

As a control, in the absence of **{Fc–N–an}** or at an applied potential anodic of the **{Fc**^**+/0**^**–N–an}** couple, <1% or TON <1 was observed for all substrates. Furthermore, these various reductions could not be achieved via direct electrolysis at an *E*_app_ set to that of the reduced anthracene moiety; at such a reducing potential, the background HER dominates, as evidenced by CV (see Supporting Information). Taken together, these data show that *pe*PCET from a mediator that operates through a long-lived CSS is a promising strategy to achieve electrocatalytic proton-coupled reductions at modest applied potentials using light as the primary driving force.

## 3 Conclusions

Strategies that harness light for selective PCET to a substrate offer an attractive approach for solar-to-chemicals conversions. In our *pe*PCET system using a 3-electrode potentiostat, the ultimate source of reducing equivalents was the reaction at the counter electrode. In most cases, this involved oxidation of a zinc electrode, although we also examined a bulk photoelectrochemical reduction of acetophenone using Fc in the counter-electrode compartment (as a sacrificial donor to recycle the ferrocene-derived *pe*PCET mediator). The reaction with Fc proceeded similarly (Figure 5), and Fc^+^ was generated in the counter-electrode compartment as expected. This approach offers attractive opportunities for photoelectrochemical catalysis using a two-electrode electrochemical cell with a counter electrode based on the HA / 1/2 H_2_ redox couple. Because *E*°(Fc^+^/Fc) is anodic of *E*°(HA / 1/2 H_2_) for acids with p*K*_a_ > 0 (Figure 1B), an applied potential would be necessary only to drive higher currents, allowing H_2_ to be both the ultimate source of electrons and protons for PCET reactivity. For acetophenone, we calculate the addition of H_2_ to be endergonic (H_2_ + 2 Ph(Me)CO → Ph(Me)(HO)C–C(OH)(Me)Ph; Δ*G*^0^ = +14 kcal mol^–1^ in MeCN at RT), implying the possibility of photochemical energy storage. Future studies will be needed to explore engineering half–cell reactions that work in synergy such that H_2_ oxidation can be partnered with reductive *pe*PCET as a means of using light to generate energy-rich chemical products. The design of second generation *pe*PCET mediators with even longer CSS lifetimes correlated to higher quantum efficiency would complement such efforts.

## Data Availability

All data are available in the main text or the Supporting Information.

## Supporting Information Available

Experimental methods; synthetic details; electrochemical data; UV-vis data; p*K*_a_ calculation; data from stoichiometric and catalysis experiments; fluorescence data; transient absorption data; reaction quantum yield determination; spectroelectrochemistry of **{Fc–N–an}**; isotope scrambling experiment; H_2_ quantification for CPE; and DFT calculations (PDF)

## References

[ref1] Weinberg D. R.; Gagliardi C. J.; Hull J. F.; Murphy C. F.; Kent C. A.; Westlake B. C.; Paul A.; Ess D. H.; McCafferty D. G.; Meyer T. J. Proton-Coupled Electron Transfer. Chem. Rev. 2012, 112 (7), 4016–4093. 10.1021/cr200177j. 22702235

[ref2] Cukier R. I.; Nocera D. G. Proton-Coupled Electron Transfer. Annu. Rev. Phys. Chem. 1998, 49, 337–369. 10.1146/annurev.physchem.49.1.337. 9933908

[ref3] Agarwal R. G.; Coste S. C.; Groff B. D.; Heuer A. M.; Noh H.; Parada G. A.; Wise C. F.; Nichols E. M.; Warren J. J.; Mayer J. M. Free Energies of Proton-Coupled Electron Transfer Reagents and Their Applications. Chem. Rev. 2022, 122 (1), 1–49. 10.1021/acs.chemrev.1c00521. 34928136 PMC9175307

[ref4] Mayer J. M. Proton-Coupled Electron Transfer: A Reaction Chemist’s View. Annu. Rev. Phys. Chem. 2004, 55, 363–390. 10.1146/annurev.physchem.55.091602.094446. 15117257

[ref5] Tyburski R.; Liu T.; Glover S. D.; Hammarström L. Proton-Coupled Electron Transfer Guidelines, Fair and Square. J. Am. Chem. Soc. 2021, 143 (2), 560–576. 10.1021/jacs.0c09106. 33405896 PMC7880575

[ref6] Gentry E. C.; Knowles R. R. Synthetic Applications of Proton-Coupled Electron Transfer. Acc. Chem. Res. 2016, 49 (8), 1546–1556. 10.1021/acs.accounts.6b00272. 27472068 PMC5102158

[ref7] Murray P. R. D.; Cox J. H.; Chiappini N. D.; Roos C. B.; McLoughlin E. A.; Hejna B. G.; Nguyen S. T.; Ripberger H. H.; Ganley J. M.; Tsui E.; Shin N. Y.; Koronkiewicz B.; Qiu G.; Knowles R. R. Photochemical and Electrochemical Applications of Proton-Coupled Electron Transfer in Organic Synthesis. Chem. Rev. 2022, 122 (2), 2017–2291. 10.1021/acs.chemrev.1c00374. 34813277 PMC8796287

[ref8] Elgrishi N.; Chambers M. B.; Fontecave M. Turning it Off! Disfavouring Hydrogen Evolution to Enhance Selectivity for CO Production During Homogeneous CO_2_ Reduction By Cobalt-Terpyridine Complexes. Chem. Sci. 2015, 6 (4), 2522–2531. 10.1039/C4SC03766A. 28706660 PMC5489026

[ref9] Ren Y.; Yu C.; Tan X.; Huang H.; Wei Q.; Qiu J. Strategies to Suppress Hydrogen Evolution for Highly Selective Electrocatalytic Nitrogen Reduction: Challenges And Perspectives. Energy Environ. Sci. 2021, 14 (3), 1176–1193. 10.1039/D0EE03596C.

[ref10] Barlow J. M.; Yang J. Y. Thermodynamic Considerations for Optimizing Selective CO_2_ Reduction by Molecular Catalysts. ACS Cent. Sci. 2019, 5 (4), 580–588. 10.1021/acscentsci.9b00095. 31041377 PMC6487447

[ref11] Smieja J. M.; Benson E. E.; Kumar B.; Grice K. A.; Seu C. S.; Miller A. J. M.; Mayer J. M.; Kubiak C. P. Kinetic and Structural Studies, Origins of Selectivity, and Interfacial Charge Transfer in the Artificial Photosynthesis of CO. Proc. Natl. Acad. Sci. U.S.A. 2012, 109 (39), 15646–15650. 10.1073/pnas.1119863109. 22652573 PMC3465436

[ref12] Peters J. C. Advancing Electrocatalytic Nitrogen Fixation: Insights from Molecular Systems. Faraday Discuss. 2023, 243 (0), 450–472. 10.1039/D3FD00017F. 37021388 PMC10524484

[ref13] Girard P.; Namy J. L.; Kagan H. B. Divalent Lanthanide Derivatives in Organic Synthesis. 1. Mild Preparation of Samarium Iodide and Ytterbium Iodide and Their Use as Reducing or Coupling Agents. J. Am. Chem. Soc. 1980, 102 (8), 2693–2698. 10.1021/ja00528a029.

[ref14] Szostak M.; Fazakerley N. J.; Parmar D.; Procter D. J. Cross-Coupling Reactions Using Samarium(II) Iodide. Chem. Rev. 2014, 114 (11), 5959–6039. 10.1021/cr400685r. 24758360

[ref15] Chciuk T. V.; Anderson W. R.; Flowers R. A. Proton-Coupled Electron Transfer in the Reduction of Carbonyls by Samarium Diiodide-Water Complexes. J. Am. Chem. Soc. 2016, 138 (28), 8738–8741. 10.1021/jacs.6b05879. 27367158

[ref16] Yan M.; Kawamata Y.; Baran P. S. Synthetic Organic Electrochemical Methods Since 2000: On the Verge of a Renaissance. Chem. Rev. 2017, 117 (21), 13230–13319. 10.1021/acs.chemrev.7b00397. 28991454 PMC5786875

[ref17] Lewis N. S. Research Opportunities to Advance Solar Energy Utilization. Science 2016, 351 (6271), aad1920 10.1126/science.aad1920. 26798020

[ref18] De Luna P.; Hahn C.; Higgins D.; Jaffer S. A.; Jaramillo T. F.; Sargent E. H. What Would it Take for Renewably Powered Electrosynthesis to Displace Petrochemical Processes?. Science 2019, 364 (6438), eaav3506 10.1126/science.aav3506. 31023896

[ref19] McCarthy B. D.; Martin D. J.; Rountree E. S.; Ullman A. C.; Dempsey J. L. Electrochemical Reduction of Brønsted Acids by Glassy Carbon in Acetonitrile—Implications for Electrocatalytic Hydrogen Evolution. Inorg. Chem. 2014, 53 (16), 8350–8361. 10.1021/ic500770k. 25076140

[ref20] Chalkley M. J.; Garrido-Barros P.; Peters J. C. A Molecular Mediator for Reductive Concerted Proton-Electron Transfers via Electrocatalysis. Science 2020, 369 (6505), 850–854. 10.1126/science.abc1607. 32792399

[ref21] Derosa J.; Garrido-Barros P.; Peters J. C. Electrocatalytic Reduction of C-C π-Bonds via a Cobaltocene-Derived Concerted Proton-Electron Transfer Mediator: Fumarate Hydrogenation as a Model Study. J. Am. Chem. Soc. 2021, 143 (25), 9303–9307. 10.1021/jacs.1c03335. 34138550

[ref22] Derosa J.; Garrido-Barros P.; Peters J. C. Electrocatalytic Ketyl-Olefin Cyclization at a Favorable Applied Bias Enabled by a Concerted Proton-Electron Transfer Mediator. Inorg. Chem. 2022, 61 (17), 6672–6678. 10.1021/acs.inorgchem.2c00839. 35436099

[ref23] Garrido-Barros P.; Derosa J.; Chalkley M. J.; Peters J. C. Tandem Electrocatalytic N_2_ Fixation via Proton-Coupled Electron Transfer. Nature 2022, 609 (7925), 71–76. 10.1038/s41586-022-05011-6. 36045240 PMC10281199

[ref24] Derosa J.; Garrido-Barros P.; Li M.; Peters J. C. Use of a PCET Mediator Enables a Ni-HER Electrocatalyst to Act as a Hydride Delivery Agent. J. Am. Chem. Soc. 2022, 144 (43), 20118–20125. 10.1021/jacs.2c09786. 36264765

[ref25] Bordwell F. G.; Cheng J. P.; Harrelson J. A. Homolytic Bond Dissociation Energies in Solution from Equilibrium Acidity and Electrochemical Data. J. Am. Chem. Soc. 1988, 110 (4), 1229–1231. 10.1021/ja00212a035.

[ref26] Pannwitz A.; Wenger O. S. Proton Coupled Electron Transfer from the Excited State of a Ruthenium(II) Pyridylimidazole Complex. Phys. Chem. Chem. Phys. 2016, 18 (16), 11374–11382. 10.1039/C6CP00437G. 27094541

[ref27] Park Y.; Kim S.; Tian L.; Zhong H.; Scholes G. D.; Chirik P. J. Visible Light Enables Catalytic Formation of Weak Chemical Bonds with Molecular Hydrogen. Nat. Chem. 2021, 13 (10), 969–976. 10.1038/s41557-021-00732-z. 34253889

[ref28] Tarantino K. T.; Liu P.; Knowles R. R. Catalytic Ketyl-Olefin Cyclizations Enabled by Proton-Coupled Electron Transfer. J. Am. Chem. Soc. 2013, 135 (27), 10022–10025. 10.1021/ja404342j. 23796403

[ref29] Parada G. A.; Goldsmith Z. K.; Kolmar S.; Pettersson Rimgard B.; Mercado B. Q.; Hammarström L.; Hammes-Schiffer S.; Mayer J. M. Concerted Proton-Electron Transfer Reactions in the Marcus Inverted Region. Science 2019, 364 (6439), 471–475. 10.1126/science.aaw4675. 30975771 PMC6681808

[ref30] Closs G. L.; Miller J. R. Intramolecular Long-Distance Electron Transfer in Organic Molecules. Science 1988, 240 (4851), 440–447. 10.1126/science.240.4851.440. 17784065

[ref31] Marcus R. A. Electron-Transfer Reactions In Chemistry - Theory And Experiment (Nobel Lecture). Angew. Chem., Int. Ed. Engl. 1993, 32 (8), 1111–1121. 10.1002/anie.199311113.

[ref32] Marcus R. A.; Sutin N. Electron Transfers in Chemistry and Biology. Biochim. Biophys. Acta, Rev. Bioenerg. 1985, 811 (3), 265–322. 10.1016/0304-4173(85)90014-X.

[ref33] Fukuzumi S.; Ohkubo K.; Suenobu T. Long-Lived Charge Separation and Applications in Artificial Photosynthesis. Acc. Chem. Res. 2014, 47 (5), 1455–1464. 10.1021/ar400200u. 24793793

[ref34] Kamimura T.; Ohkubo K.; Kawashima Y.; Ozako S.; Sakaguchi K.; Fukuzumi S.; Fumito T. Long-Lived Photoinduced Charge Separation in Inclusion Complexes Composed of a Phenothiazine-Bridged Cyclic Porphyrin Dimer and Fullerenes. J. Phys. Chem. C 2015, 119, 25634 10.1021/acs.jpcc.5b09147.

[ref35] Farrugia T. J.; Magri D. C. ‘Pourbaix Sensors’: A New Class of Fluorescent pE-pH Molecular AND Logic Gates Based on Photoinduced Electron Transfer. New J. Chem. 2013, 37 (1), 148–151. 10.1039/C2NJ40732A.

[ref36] Chapin B. M.; Metola P.; Vankayala S. L.; Woodcock H. L.; Mooibroek T. J.; Lynch V. M.; Larkin J. D.; Anslyn E. V. Disaggregation is a Mechanism for Emission Turn-On of ortho-Aminomethylphenylboronic Acid-Based Saccharide Sensors. J. Am. Chem. Soc. 2017, 139 (15), 5568–5578. 10.1021/jacs.7b01755. 28358506

[ref37] McCleskey T. M.; Winkler J. R.; Gray H. B. Driving-Force Effects on the Rates of Bimolecular Electron-Transfer Reactions. J. Am. Chem. Soc. 1992, 114 (17), 6935–6937. 10.1021/ja00043a060.

[ref38] Krechkivska O.; Wilcox C. M.; Nauta K.; Kable S. H.; Schmidt T. W. Quantum-Induced Symmetry Breaking in the Deuterated Dihydroanthracenyl Radical. J. Phys. Chem. A 2019, 123 (31), 6711–6719. 10.1021/acs.jpca.9b04561. 31310135

[ref39] Sooväli L.; Kaljurand I.; Kütt A.; Leito I. Uncertainty Estimation in Measurement of pKa Values In Nonaqueous Media: A Case Study on Basicity Scale in Acetonitrile Medium. Anal. Chim. Acta 2006, 566 (2), 290–303. 10.1016/j.aca.2006.03.020.

[ref40] Howell J. O.; Wightman R. M. Ultrafast Voltammetry of Anthracene and 9,10-diphenylanthracene. J. Phys. Chem. 1984, 88 (18), 3915–3918. 10.1021/j150662a001.

[ref41] While our computationally obtained BDFE_X-H_ values are internally consistent for this study, other values may be derived using different computational models, for example that of the acetophenone O–H ketyl radical species.^41^ Due to an inherent uncertainty in the estimation of the free energy of H^•^, we address these discrepancies by attributing to all of our computationally derived BDFE_X–H_ values an error of ±4 kcal mol^–1^. (See Supporting Information for further details).

[ref42] Qiu G.; Knowles R. R. Rate-Driving Force Relationships in the Multisite Proton-Coupled Electron Transfer Activation of Ketones. J. Am. Chem. Soc. 2019, 141 (6), 2721–2730. 10.1021/jacs.8b13451. 30665301 PMC6508549

