# Supplementary material for: Intermolecular Proton-Coupled Electron Transfer Reactivity from a Persistent Charge-Transfer State for Reductive Photoelectrocatalysis
Source: J Am Chem Soc. 2024 Apr 26;146(18):12750–7. doi: 10.1021/jacs.4c02610 (PMC11082884; doi:10.1021/jacs.4c02610)
Supplement: Supplementary file 1 — ja4c02610_si_001.pdf [file ja4c02610_si_001.pdf]

Supporting Information for

Intermolecular Proton-coupled Electron Transfer Reactivity from a Persistent Charge-Transfer State for Reductive Photoelectrocatalysis

Pablo Garrido-Barros,‡ Catherine G. Romero,‡ Jay R. Winkler,* and Jonas C. Peters*

Division of Chemistry and Chemical Engineering, California Institute of Technology (Caltech); Pasadena, California 91125, United States

Table of Contents

S1. General experimental information……………………………………………2-4
S2. Synthetic details…………………………………………………………………..5-11
S3. Electrochemical data……………………………………………………………12-16
S4. UV-vis data……………………………………………………………………17-19
S5. pK_a calculation………………………………………………………………..20-21
S6. Stoichiometric photochemical reactions………………………………………...22-31
S7. Fluorescence data……………………………………………………………….32-35
S8. Transient absorption data………………………………………………………..36-67
S9. Reaction quantum yield determination…………………………………………...68-69
S10. Spectroelectrochemistry of **{Fc–N–an}**……………………………………...70-71
S11. Isotope scrambling experiment………………………………………………...72
S12. Photoelectrocatalytic reactions………………………………………………...73-80
S13. H_2 Quantification for CPE……………………………………………………81
S14. Stoichiometric reaction with $W(dppe)_2(NNH_2)(OTf)_2$………………………82-87
S15. DFT calculations………………………………………………………………88-95
S16. References……………………………………………………………………96-97

S1. General Experimental Information

S1.1 Chemical/Reagent Considerations:

All manipulations were carried out using standard Schlenk or glovebox techniques under an N_2 or Ar atmosphere as specified. Solvents were deoxygenated and dried by thoroughly sparging with N_2 followed by passage through an activated alumina column in a solvent purification system by SG Water, USA LLC. Subsequently, the solvents were further dried and stored under N_2 atmosphere inside a glove box with molecular sieves obtained from Sigma Aldrich that were activated at 200ºC overnight under vacuum. Non-halogenated solvents were tested with sodium benzophenone ketyl in tetrahydrofuran (THF) in order to confirm the absence of oxygen and water. Deuterated DMSO-d_6, CD_3CN, and $CDCl_3$ solvents (D, 99.9% with a purity of 99.5%) were purchased from Cambridge Isotope Laboratories, Inc., and used as received.

N_2 gas in a NEXUS glovebox (Vacuum Atmospheres Company) was purified by a Nexus modular purification system. The purity of N_2 gas was assessed via colorimetric, gas chromatography and NMR methods, with regard to NH_3, N_2O, NO_2^- and NO_3^- impurities.[1]

Triflic acid (HOTf), ferrocene, ferrocenecarboxaldehyde, bis(*p*-toluenesulfonyl)amine ($HNTs_2$), 2-picoline, tetrabutylammonium triflate ([TBA][OTf]), and methyl triflate (MeOTf) were all used as purchased from Sigma Aldrich. Tetrabutylammonium hexafluorophosphate ([TBA][PF_6]) from Sigma Aldrich was recrystallized from ethanol prior to use. Silver triflate (AgOTf) was purchased from Strem and used without further purification. Whenever water was specified as solvent, deionized water OmniSolv (Supelco, Sigma Aldich) was used to prepare the solutions.

Teflon-coated magnetic stir bars were soaked in concentrated nitric acid for at least 1 h, washed repeatedly with deionized water then acetone, and dried in an oven prior to use. In air- or moisture-sensitive reactions, solvents were deoxygenated and dried by thoroughly sparging with N_2 followed by passage through an activated alumina column in a solvent purification system by SG Water, USA LLC.

S1.2 NMR Spectroscopy:

^{1}H, ^{31}P, and ^{15}N spectra were recorded with Varian or Bruker 400 MHz spectrometers. Spectra were internally referenced to solvent signals when deuterated solvents were used or an internal standard. ^{15}N-NMRs are referenced to liquid NH_3 scale.

S1.3 UV-Vis Spectroscopy:

Measurements were taken on a Cary 50 UV-Visible spectrophotometer using a 1 cm quartz cell sealed with a Teflon stopcock.

S1.4 Electrochemistry:

A CHI instrument 600B and Biologic VSP potentiostat were used for all electrochemical data collection. Cyclic voltammetry (CV) experiments were carried out in a one-compartment three-electrode cell using a boron doped diamond (BDD) disk as the working electrode (3 mm diameter),

a glassy carbon (GC) disk as the counter electrode, and a Ag/AgOTf (5 mM) reference electrode. Details for the CVs are noted as they appear. $E_{1/2}$ values for the reversible waves were obtained from the half potential between the oxidative and reductive peaks. For all measurements, IR compensation was applied accounting for 85% of the total resistance. All the reported potentials are referenced to the ferrocenium/ferrocene couple ($Fc^{+/0}$). The BDD disk electrode for cyclic voltammetry was polished using a PK-3 polishing kit with 6 μm diamond (Biologic), while the GC electrode was polished using alumina 0.05 μm powder.

S1.5 Spectroelectrochemistry

Spectroelectrochemistry was performed in a nitrogen-filled glove box with a Biologic SP-300 potentiostat. Measurements were performed with a quartz spectroelectrochemical cell with a 0.17 cm path length from Pine Research Instrumentation (AKSTCKIT3), an Ag wire pseudo-reference electrode (Kurt J. Lesker), a gold honeycomb electrode (Pine Instruments), and a platinum wire counter electrode (Kurt J. Lesker). Potentiostatic electrochemical impedance spectra (PEIS) were recorded to obtain Nyquist plots to determine the uncompensated resistance. 95% of the uncompensated resistance was accounted for using electronic compensation. Measurements were recorded using a StellarNet SL4 deuterium and tungsten halogen UV-vis-NIR light source coupled to StellarNet Black Comet UV-vis and DWARF-Star NIR spectrometers. All spectra were acquired in THF solution with 0.7 M $TBAPF_6$ electrolyte.

S1.6 Gas chromatography:

Gas chromatography coupled to a thermal conductivity detector (GC-TCD) was used for H_2 quantification. A 100 μL Hamilton syringe was used to sample the headspace and to inject into the GC-TCD. GC-TCD was performed in the Environmental Analysis Center (Caltech) using a HP 5890 Series II instrument with N_2 as the carrier gas. Calibration was determined by direct injection of known volumes of H_2 according to previous work with this instrument.[2]

S1.7 Luminescence and Transient Absorption

Time-resolved luminescence measurements and transient absorption measurements were carried out in the Beckman Institute Laser Resource Center at Caltech. All measurements were performed with samples under an N_2 atmosphere at room temperature. Samples were prepared in air-tight 1 cm path length quartz cuvettes in a dark, N_2 filled glovebox. Prior to measurement, all samples were protected from light by wrapping in aluminum foil.

Steady-state Luminescence

Fluorescence spectra were recorded using a modified Jobin Yvon Fluorolog instrument. Excitation was provided by a 450 W Xe arc lamp, wavelength-selected with a 0.25 m monochromator. Luminescence was collected at 90° with reflective optics, focused onto an optical fiber bundle and fed to an Ocean Optics QE Pro cooled CCD spectrometer. The instrument was controlled with software written in MATLAB. All spectra were corrected for instrument response.

Time-resolved Fluorescence

Laser excitation was provided by regeneratively amplified (Continuum) pulses from a diode-pumped passively mode-locked Nd:YAG laser (Spectra Physics Vanguard 2000). The output from the regenerative amplifier was tripled (355 nm, ~10 ps) and directed on to the sample held in a stirred 1-cm fluorescence cuvette. Fluorescence was collected at 90° using reflective optics and focused onto the entrance slit of a 0.275 m spectrograph (Acton SpectraPro 275). A fiber bundle at the spectrograph image plane collected the fluorescence and directed it to the entrance slit of a picosecond streak camera (Hamamatsu C5680). The streak camera was operated in photon-counting mode using High Performance Digital Temporal Analyzer software (Hamamatsu). The instrument time resolution is ~20 ps.

Transient Absorption

Samples in stirred air-tight cuvettes were excited with 355-nm pulses (~8 ns) from a Q-switched Nd:YAG laser (Spectra-Physics Quanta-Ray PRO-Series) or a tunable Nd:YAG-pumped optical parametric oscillator (Opotek Radiant QX8130U), both operating at a 10 Hz repetition rate. Probe light from a current-pulsed (1 ms) 75-W Xe arc lamp was directed with all-reflective optics colinearly with the laser excitation through the cuvette and wavelength selected using a double monochromator (ISA DH10). Wavelength-selected probe light was detected by a photomultiplier tube (PMT, Hamamatsu R955) wired for 5 gain stages. The PMT output was amplified (Femto DHPCA-100), offset in a wideband differential amplifier, and digitized at speeds up to 1 GS/s with a transient digitizer (GageScope). For luminescence decay measurements the probe light was blocked and sample fluorescence was detected by the PMT. For timescales >400 μs the Xe arc lamp was operated in CW mode. Data collection was controlled by a PC with software written in LabView (National Instruments). Signals were averaged for several hundred laser shots to optimize signal-to-noise levels.

S1.8 Computational details

All DFT calculations were performed in the Gaussian 09,[3] using the TPSS (meta-GGA)[4] functional with def2-TZVPP[5,6] on all atoms, Grimme-d3 dispersion correction,[7] and, when explicitly indicated, SMD[8] implicit solvation modelling acetonitrile for thermochemical parameters (for direct comparison with experimental available data). Geometry optimizations were computed in solution without symmetry restrictions. All calculated structures were stationary points as confirmed by single-point vibrational frequency calculations. Free energy corrections were calculated at 298.15 K and 105 Pa pressure, including zero-point energy corrections (ZPE). Unless otherwise mentioned, all reported energy values are free energies in solution under standard state conditions. Reduction potentials were determined via calculated exchange reactions with ferrocene/ferrocenium. The pK_a values were likewise determined via exchange reactions with 2-chloroanilinium/2-chloroaniline.[9] The bond dissociation free energies (BDFEs) were calculated directly based on the energy for the different species involved in the H-atom transfer reaction SubH· → Sub + H·, using the free energy of H·. This DFT methodology has already been proven to successfully reproduce the thermodynamic data associated with this catalytic system as reported in previous publications.

S2. Synthetic details

{Fc–N–an},[10] $W(N_2)_2(dppe)_2$,[11] diphenylfumarate,[12] and **{N–an}**[13] were all synthesized according to literature procedures.

2-picolinium triflate ([PicH][OTf] and [PicD][OTf]) 1 mL (0.94 g, 10 mmol) 2-picoline was dissolved in 3 mL diethyl ether. 1 mL (1.7 g, 11 mmol) trifluoromethanesulfonic acid (HOTf) was dissolved separately in 2 mL diethyl ether, and both were cooled to -78 °C for 20 minutes. At -78 °C, the HOTf solution was added dropwise to the solution of 2-picoline which resulted in the immediate formation of a white precipitate. The suspension was stirred at room temperature for an additional 30 minutes before washing the solid with 20 mL of diethyl ether and drying *in vacuo*. The resulting solid was dissolved in 2 mL THF, layered with 3 mL diethyl ether, and kept undisturbed at -35 °C overnight, forming large crystals of [PicH][OTf]. (2.4 g, 99 % yield). **^{1}H-NMR** (400 MHz, DMSO-*d_6*) δ 2.73 (s, 3H), δ 7.91 (m, 2H), δ 8.48 (td, 1H), δ 8.80 (dd, 1H), δ 15.5 (br s, 1H). [PicD][OTf], used for the KIE study, was prepared in the same manner using DOTf, and resulting in an 85% D label by ^{1}H-NMR.

1,2-dimethylpyridinium ([PicMe][OTf]) 200 µL (0.19 g, 2 mmol) 2-picoline was dissolved in 2 mL diethyl ether. 220 µL (0.33 g, 2 mmol) methyl trifluoromethanesulfonate (MeOTf) was dissolved separately in 1 mL diethyl ether, and both were cooled to -78 °C for 20 minutes. At -78 °C, the MeOTf solution was added dropwise to the solution of 2-picoline which resulted in the immediate formation of a white precipitate. The suspension was stirred at room temperature for an additional 30 minutes before washing the solid with 20 mL of diethyl ether and drying *in vacuo*. (0.50 g, 96 % yield). **^{1}H-NMR** (400 MHz, MeCN-*d_3*) δ 2.73 (s, 3H), δ 4.13 (s, 3H), δ 7.83 (m, 2H), δ 8.34 (td, 1H), δ 8.56 (dd, 1H)

{Fc–NH$^+$–an}OTf The monoprotonated complex was synthesized by pronation with HOTf as follows: 50 mg (0.12 mmol) **{Fc–N–an}** was dissolved in 2 mL anhydrous diethyl ether. A solution of 10.5 µL HOTf (0.12 mmol) was dissolved separately in 1 mL anhydrous diethyl ether. While stirring, the solution of HOTf was added dropwise to that of **{Fc–N–an}** and stirred at room temperature for 15 minutes, after which a yellow precipitate formed. The precipitate was washed with 10 mL anhydrous diethyl ether and 5 mL pentane before being fully dried *in vacuo*. (61 mg, 90 % yield). **^{1}H-NMR** (400 MHz, DMSO-*d_6*) δ 2.60 (d, 3H), δ 4.32 (s, 5H), δ 4.56 (m, 6H), δ 5.13 (m, 1H), δ 5.39 (d, 1H), δ 7.63 (m, 4H), δ 8.19 (d, 2H), δ 8.78 (s, 1H) **^{19}F-NMR** (400 MHz, DMSO-*d_6*) δ -79.35

{Fc–NMe$^+$–an}OTf The methylated complex was synthesized with MeOTf as follows: 10 mg (0.024 mmol) **{Fc–N–an}** was dissolved in 2 mL anhydrous diethyl ether. 2.6 µL MeOTf (0.024 mmol) was dissolved separately in 1 mL anhydrous diethyl ether. While stirring, the solution of MeOTf was added dropwise to that of **{Fc–N–an}** and stirred at room temperature for 15 minutes, after which a yellow precipitate formed. The precipitate was washed with 10 mL anhydrous diethyl ether and 5 mL pentane before being fully dried *in vacuo*. (12 mg, 86 % yield). **^{1}H-NMR** (400 MHz, MeCN-*d_3*) δ 2.72 (s, 6H), δ 4.19 (s, 5H), δ 4.41 (s, 2H), δ 4.49 (s, 2H), δ 4.55 (s, 2H), δ 5.53 (s, 2H), δ 7.61 (t, 2H), δ 7.72 (t, 2H), δ 8.20 (d, 2H), δ 8.31 (d, 2H), δ 8.85 (s, 1H) **^{19}F-NMR** (400 MHz, MeCN-*d_3*) δ -79.36

W(NNH$_2$)(dppe)$_2$(OTf)$_2$ W(N$_2$)$_2$(dppe)$_2$ (25 mg, 0.024 mmol) was dissolved in 5 mL dry benzene in an N$_2$ filled glovebox. HOTf (4.3 µL, 0.048 mmol) was diluted with 300 µL benzene and added dropwise with stirring. The resulting buff-colored solution was stirred for 15 minutes at room temperature and then lyophilized (31 mg, 99 % yield). **^{1}H-NMR** (400 MHz, C$_6$D$_6$) δ 2.49 (m, 4H), δ 2.62 (m, 4H), δ 5.17 (brs, 2H, NN**H$_2$**), δ 6.83 (m, 8H), δ 6.96 (m, 16H), δ 7.11 (m, 8H), δ 7.36 (m, 8H) **^{19}F-NMR** δ -78.3, δ -76.5 **^{31}P-NMR** δ 37.2. The ^{15}N-labeled compound was synthesized using W(^{15}N$_2$)$_2$(dppe)$_2$ which was synthesized according to literature procedure [14] under ^{15}N$_2$ and handled using standard Schlenk techniques under Ar. The spectral features are analogous to the unlabeled compound except the following: **^{1}H-NMR** (400 MHz, C$_6$D$_6$) δ 5.39 (d, 2H, ^{15}N^{15}N**H$_2$** *J* (^{15}N–^{1}H) = 92 Hz) **^{31}P-NMR** δ 37.2 (d, *J* (^{15}N–^{31}P) = 4.4 Hz.

NMR spectra

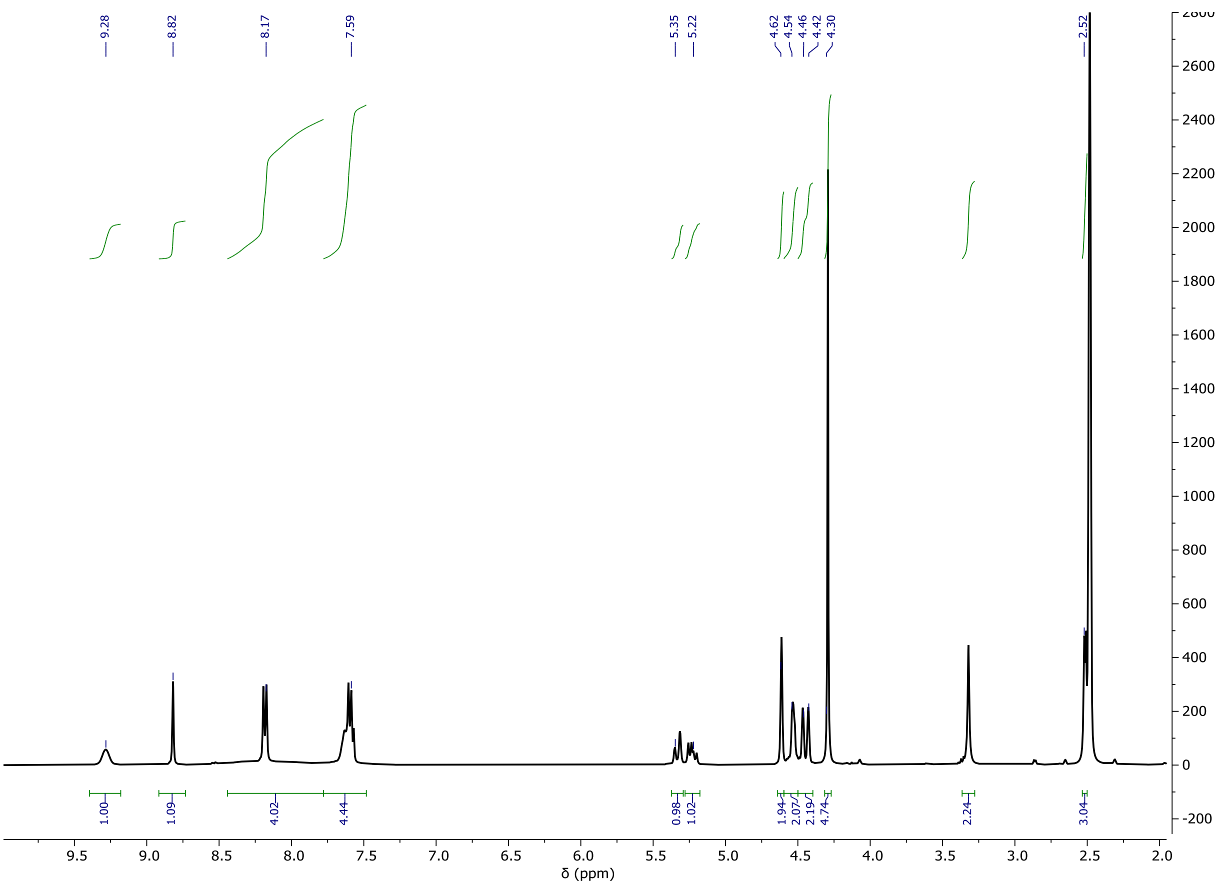

Fig. S1. ^{1}H NMR spectrum (400 MHz, DMOS-d_6) of **{Fc–NH$^+$–an}** generated *via* protonation with 1 equivalent HOTf.

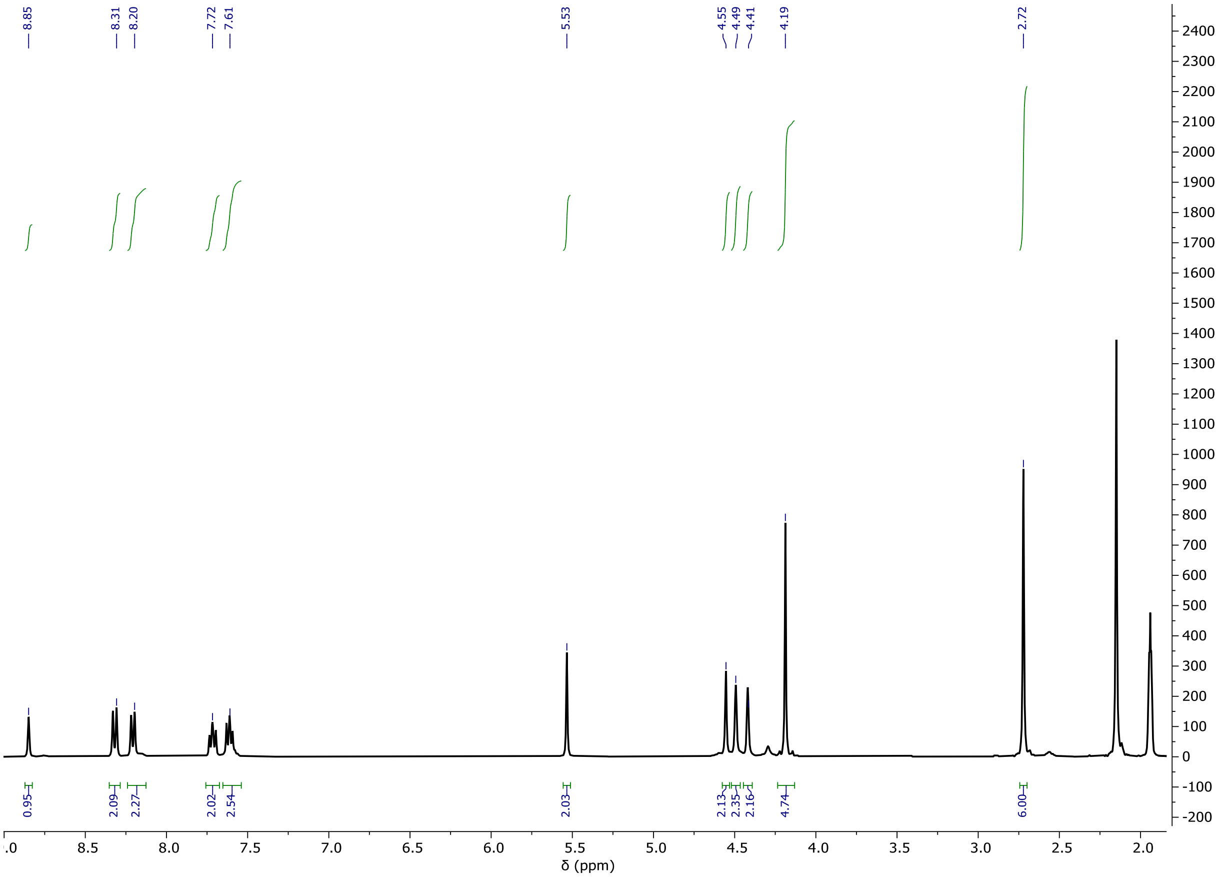

Fig. S2. ^{1}H NMR spectrum (400 MHz, MeCN-d_3) of **{Fc–NMe$^+$–an}** generated with 1 equivalent MeOTf.

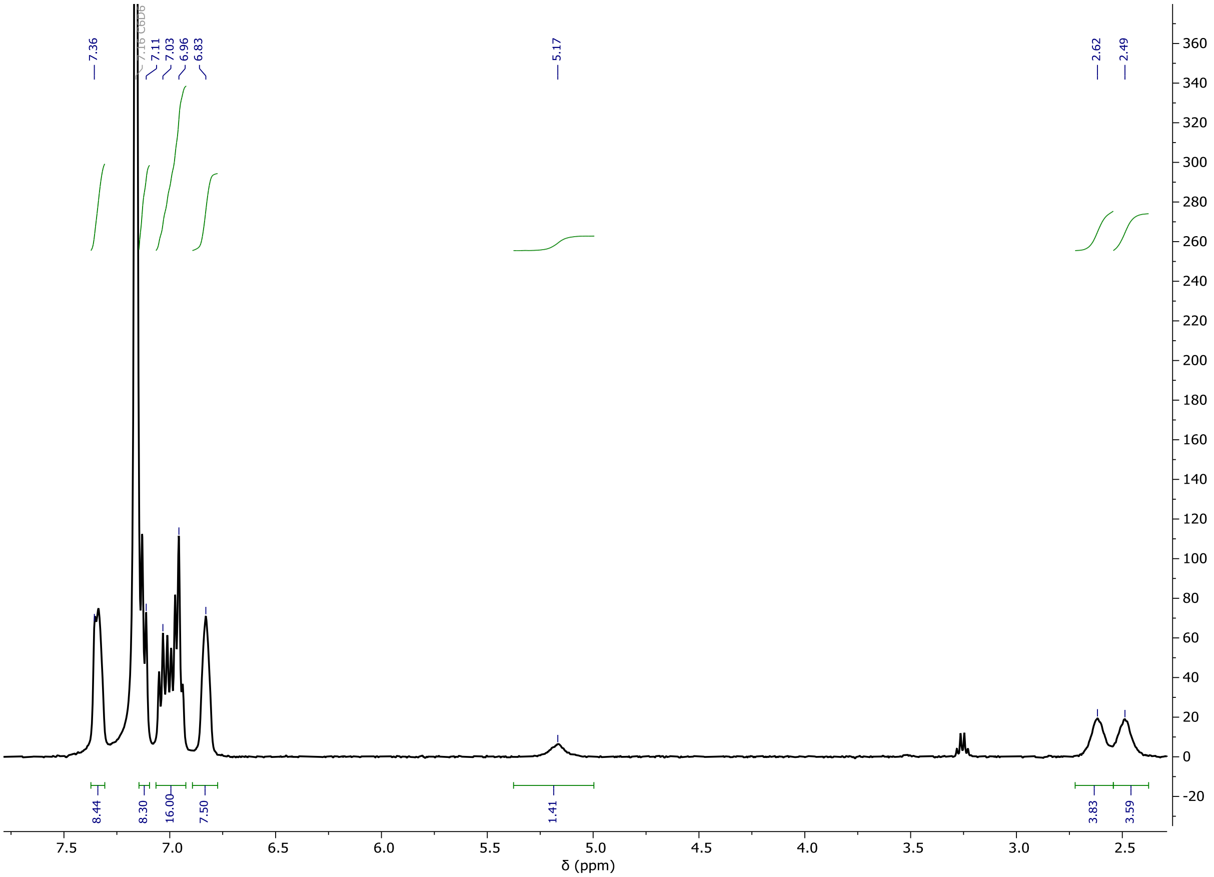

Figure S3. ^{1}H-NMR spectrum (400 MHz, C_6D_6) of $W(NNH_2)(dppe)_2(OTf)_2$.

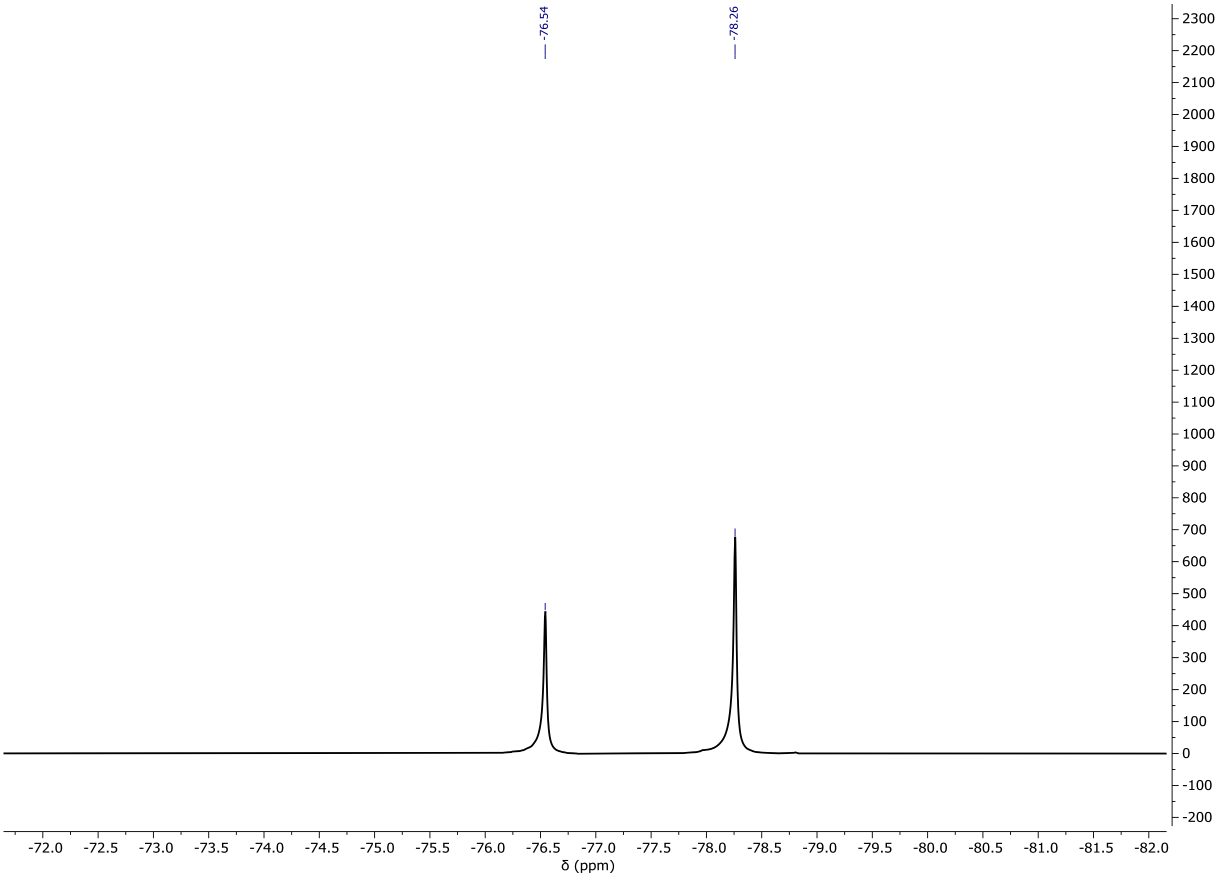

Figure S4. ^{19}F-NMR of $W(NNH_2)(dppe)_2(OTf)_2$ in C_6D_6.

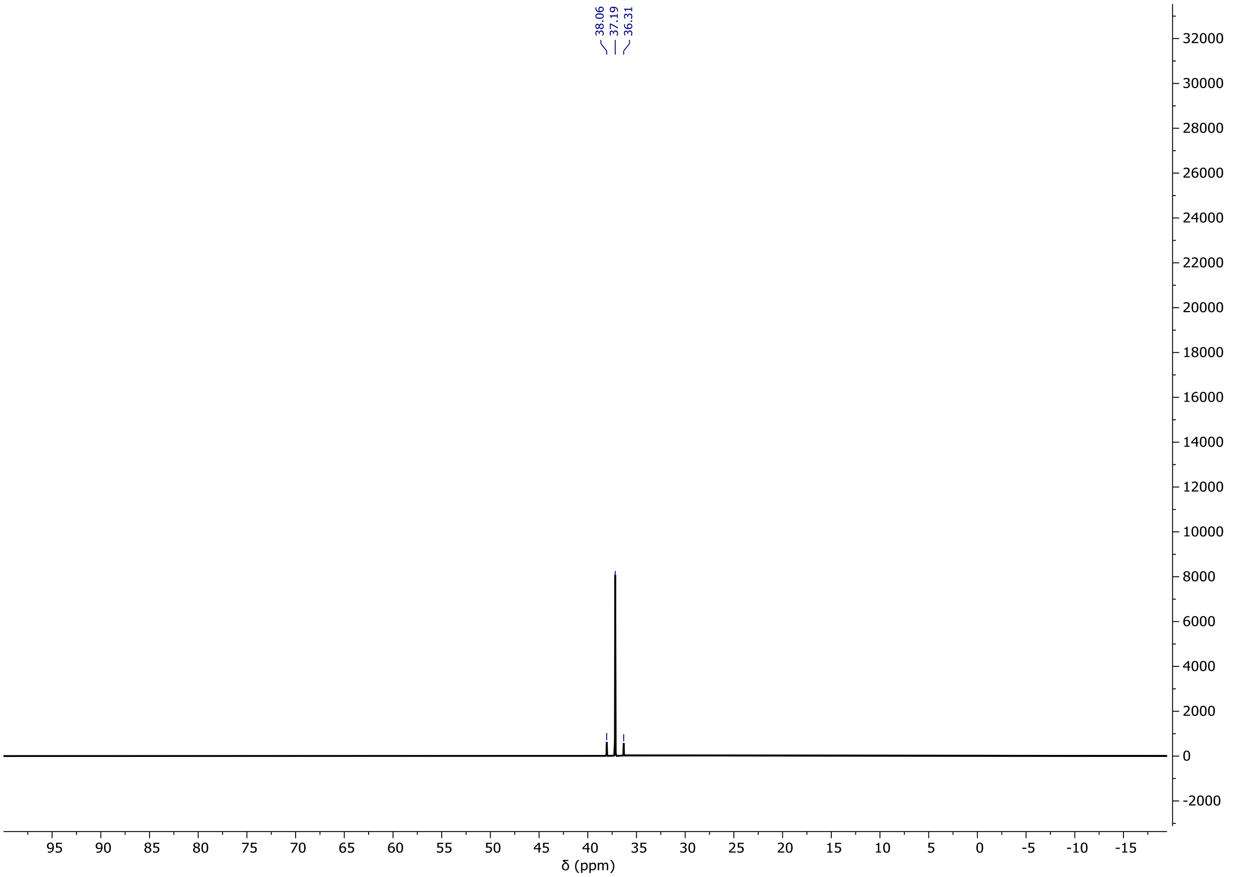

Figure S5. ^{31}P-NMR of $W(NNH_2)(dppe)_2(OTf)_2$ in C_6D_6.

S3. Electrochemical data

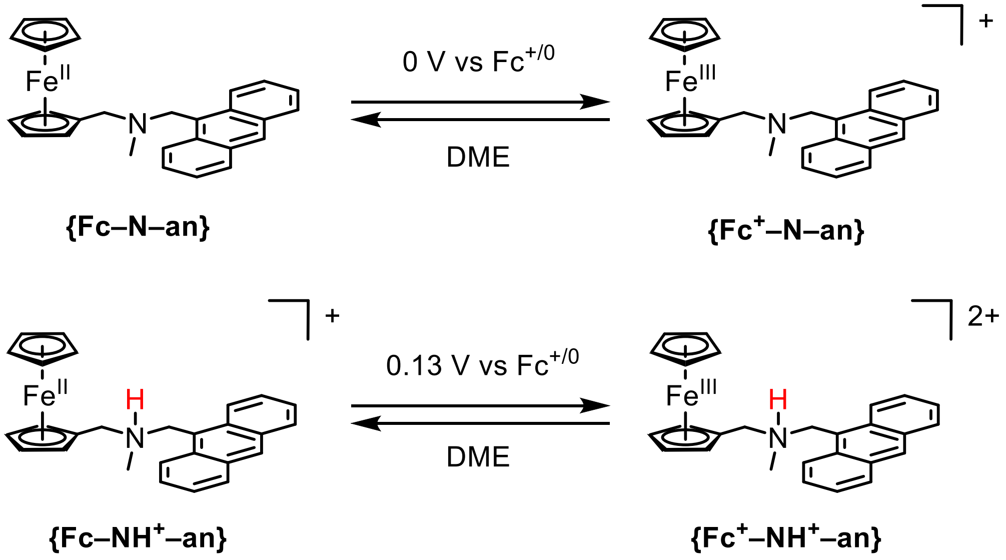

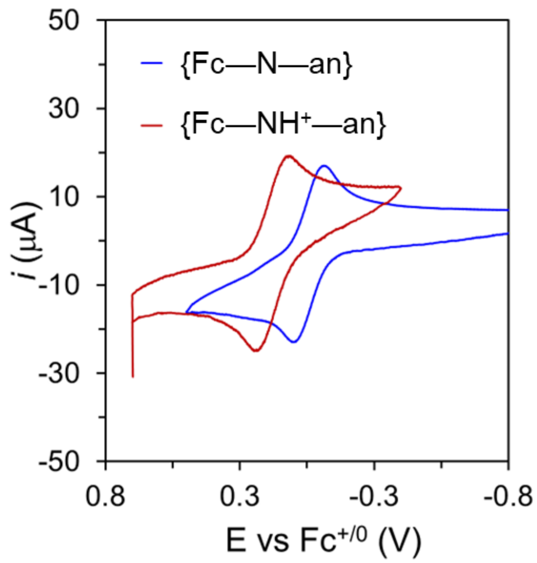

Fig. S6. Schematic representation of the anodic redox processes observed for **{Fc–N–an}** and **{Fc–NH^{+}–an}** and the corresponding cyclic voltammetry of these compounds in a 1 mM solution in DME containing 0.1 M [TBA][PF_6] using a glassy carbon disk working electrode, a Ag/AgOTf reference electrode and a Pt disk counter electrode at 100 mV·s^{-1}.

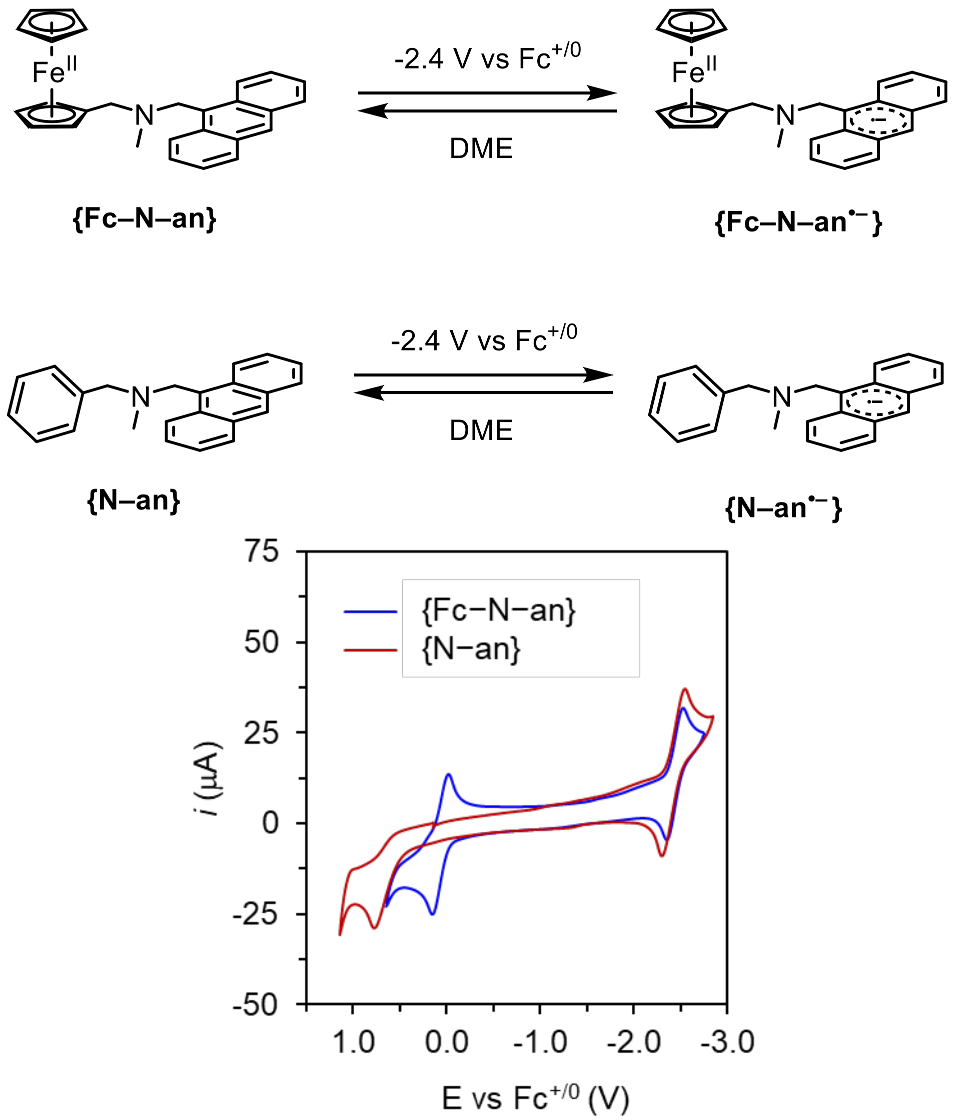

Fig. S7. Schematic representation of the anodic and cathodic redox processes observed for **{Fc–N–an}** and **{N–an}** and the corresponding cyclic voltammetry of these compounds in a 1 mM solution in DME containing 0.1 M [TBA][PF_6] using a glassy carbon disk working electrode, a Ag/AgOTf reference electrode and a Pt disk counter electrode at 100 mV·s^{-1}.

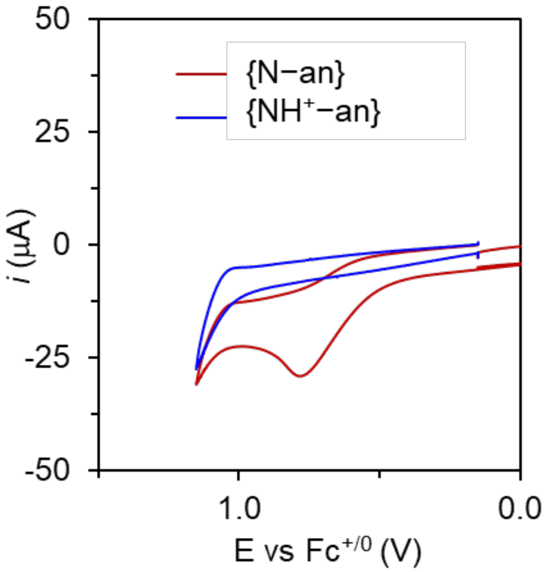

Fig. S8. Cyclic voltammetry of a 1 mM **{N–an}** (red) and **{NH$^+$–an}** (blue) solution in DME containing 0.1 M [TBA][PF_6] using a glassy carbon disk working electrode, a Ag/AgOTf reference electrode and a Pt disk counter electrode at 100 mV·s^{-1}.

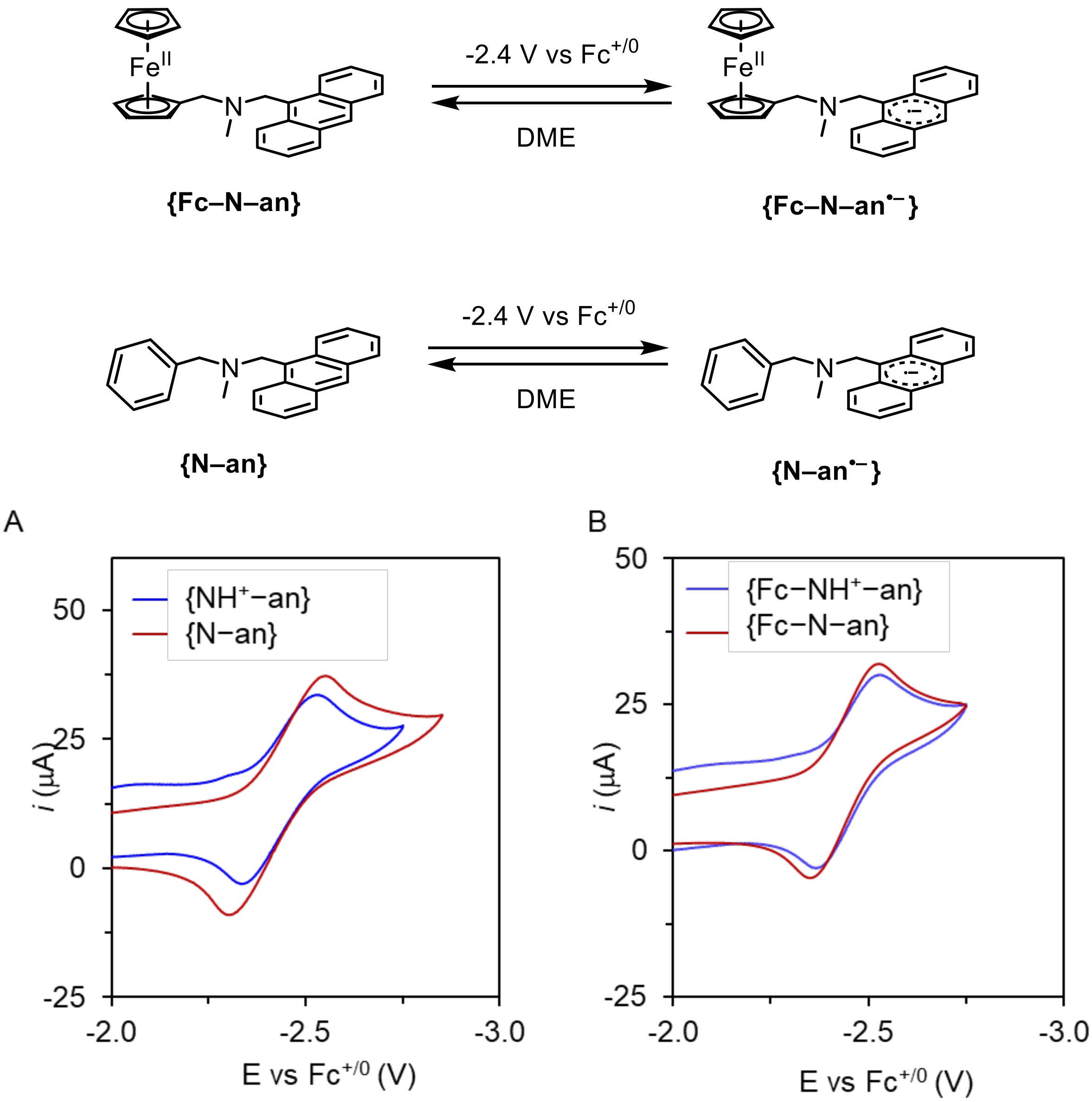

Fig. S9. (A) Cyclic voltammetry of a 1 mM **{NH⁺–an}** (blue trace) and **{N–an}** (red trace) solution in DME containing 0.1 M [TBA][PF_6]. (B) Cyclic voltammetry of a 1 mM **{Fc–NH⁺–an}** (blue trace) and **{Fc–N–an}** (red trace) solution in DME containing 0.1 M [TBA][PF_6]. For both sets of CVs, we employed a glassy carbon disk working electrode, a Ag/AgOTf reference electrode and a Pt disk counter electrode at 100 $mV\cdot s^{-1}$. The schematic representation of the redox processes is shown above the CV figures.

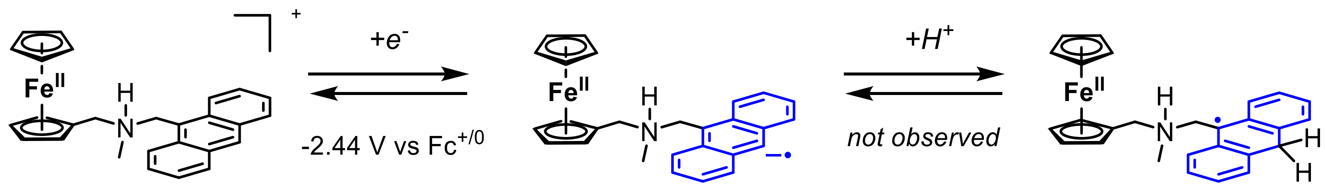

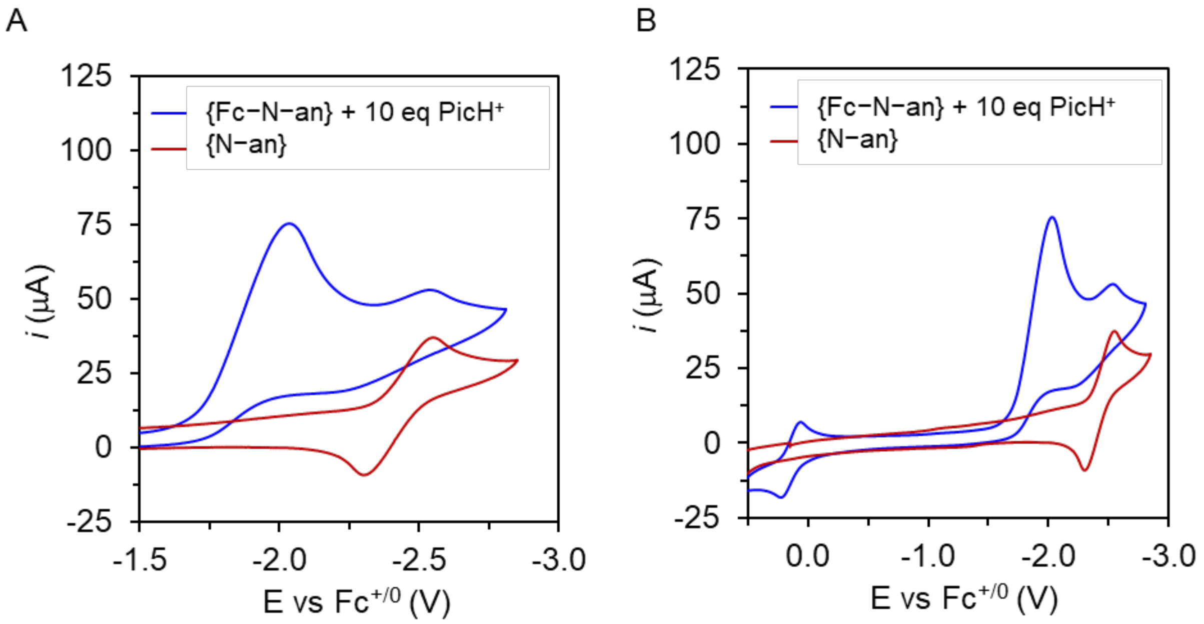

Fig. S10. Cyclic voltammetry of a 1 mM **{N–an}** (red trace) and 1 mM **{Fc–N–an}** with 10 equivalents of [PicH][OTf] (red trace) solution in DME containing 0.1 M [TBA][PF_6] using a glassy carbon disk working electrode, a Ag/AgOTf reference electrode and a Pt disk counter electrode at 100 mV·s^{-1}. Figure A shows an enlargement of the more cathodic region while Figure B displays the anodic processes associated to **{Fc–NH$^+$–an}**. The larger peak at around -1.8 is associated to electrode mediated hydrogen evolution by the [PicH][OTf]. These CVs show the absence of any shift in the redox potential of the most anodic process associated to an → an$^{\cdot-}$ and thus the lack of chemical reaction with protons upon reduction. The availability of protons in the double layer is evidenced by the larger current obtained at potentials < –2 V in the presence of [PicH][OTf] and the potential of the anodic wave associated to the protonated form **{Fc–NH$^+$–an}**.

S4. UV-vis data

In situ preparation of the protonated ***{Fc–NH⁺–an}*** *complex with different acids*

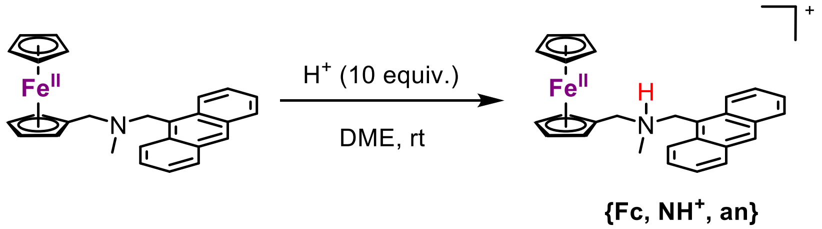

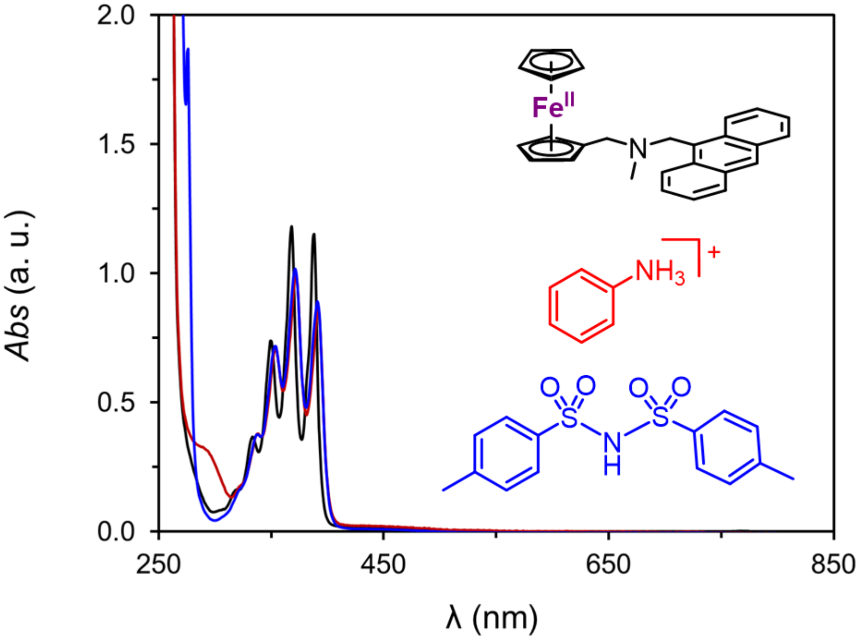

Fig. S11. UV-vis spectra of a 0.5 mM solution of **{Fc–N–an}** in DME (black trace) and with the addition of 10 equivalents of either $[PhNH_3][OTf]$ (red trace) or $(Tos)_2NH$ (blue trace) acids. The small redshift observed upon addition of both acids evidences the protonation at the amine group in **{Fc–N–an}** to form in situ **{Fc–NH$^+$–an}**.

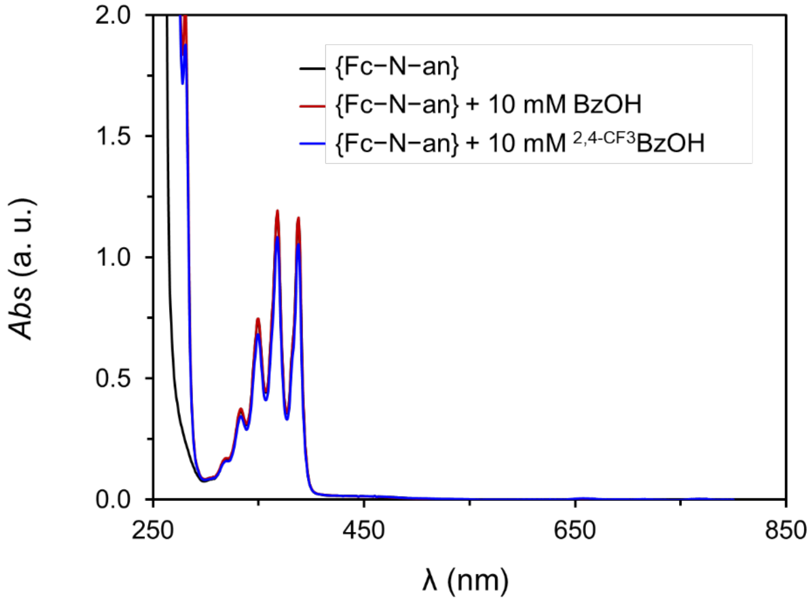

Fig. S12. UV-vis spectra of a 0.5 mM solution of **{Fc–N–an}** in DME (black trace) and with the addition of 10 equivalents of either BzOH (benzoic acid, red trace) or $^{2,4\text{-}CF_3}$BzOH (2,4-bis(trifluoromethyl)benzoic acid, blue trace) acids. The absence of any shift in the maximum absorption bands upon addition of both acids reveals the lack of protonation of **{Fc–N–an}**.

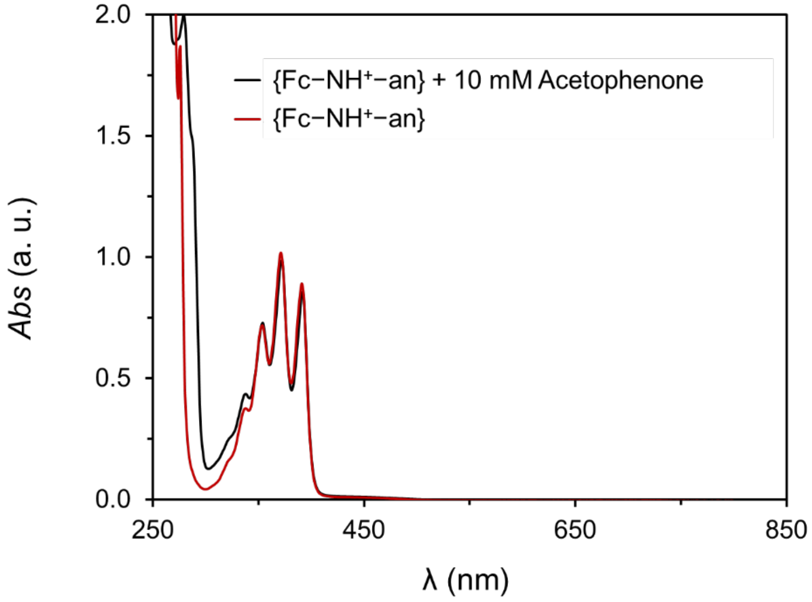

Fig. S13. UV-vis spectra of a 0.5 mM solution of **{Fc–NH$^+$–an}** in DME (red trace) and with the addition of 20 equivalents of acetophenone showing the absence of any relevant change.

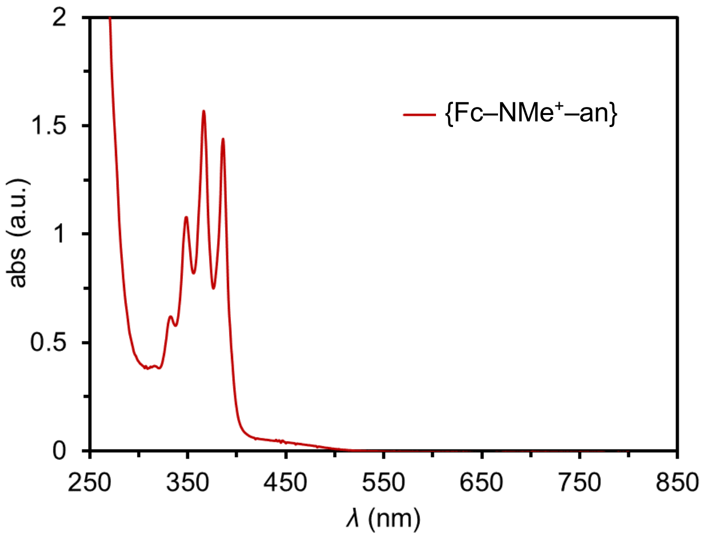

Fig. S14. UV-vis spectra of a 0.05 mM solution of **{Fc–NMe^{+}–an}** in DME.

S5. pK_a calculation

The pK_a of the **{Fc–N–an}** complex to form **{Fc–NH$^+$–an}** was calculated based on ^{1}H NMR titration using anilinium triflate [$PhNH_3$][OTf] acid in CD_3CN. **{Fc–N–an}** is dissolved in CD_3CN and an initial NMR is taken. Subsequently, aliquots of a CD_3CN stock solution containing [$PhNH_3$][OTf] (20 mM) are added to monitor changes in the chemical shifts upon protonation. The relative concentration of the protonated and deprotonated species is determined from the chemical shift of the representative peaks relative to the chemical shifts of the protonated and deprotonated species analyzed independently, using equations S1 and S2:

$$f(\{Fc\text{–}N\text{–}an\}) = \frac{\delta - \delta_{\{Fc,NH^+,an\}}}{\delta_{\{Fc,N,an\}} - \delta_{\{Fc,NH^+,an\}}} \quad Eq.S1$$

$$f(PhNH_3{}^+) = \frac{\delta - \delta_{PhNH_2}}{\delta_{PhNH_3{}^+} - \delta_{PhNH_2}} \quad Eq.S2$$

Having the fraction of each component for the different NMR samples, the concentration of each component was calculated assuming a normalized concentration for [**{Fc–N–an}** + [**{Fc–NH$^+$–an}**] of 1 and using the relative integral of the peaks for [$PhNH_3^+$ + $PhNH_2$] to calculate the relative concentration of the latter. Using this total normalized concentration of [$PhNH_3^+$ + $PhNH_2$] and [**{Fc–N–an}** + [**{Fc–NH$^+$–an}**] and the fractions calculated previously, the concentration for each species and thus the equilibrium constant for the protonation process can be calculated according to the following equations:

$$[\{Fc,N,an\}] = f(\{Fc,N,an\})$$

$$[\{Fc\text{–}NH^+\text{–}an\}] = 1 - [\{Fc\text{–}N\text{–}an\}]$$

$$[PhNH_3{}^+] = f(PhNH_3{}^+) \cdot [PhNH_3{}^+ + PhNH_2{}^+]$$

$$[PhNH_2] = [PhNH_3{}^+ + PhNH_2{}^+] - [PhNH_3{}^+]$$

$$K_{eq} = \frac{[PhNH_2][\{Fc\text{–}NH^+\text{–}an\}]}{[PhNH_3{}^+][\{Fc\text{–}N\text{–}an\}]} \quad Eq.S3$$

A pK_a of 10.62 for $PhNH_3^+$ was used,[15] resulting in a pK_a of 14.3 for **{Fc–NH$^+$–an}**.

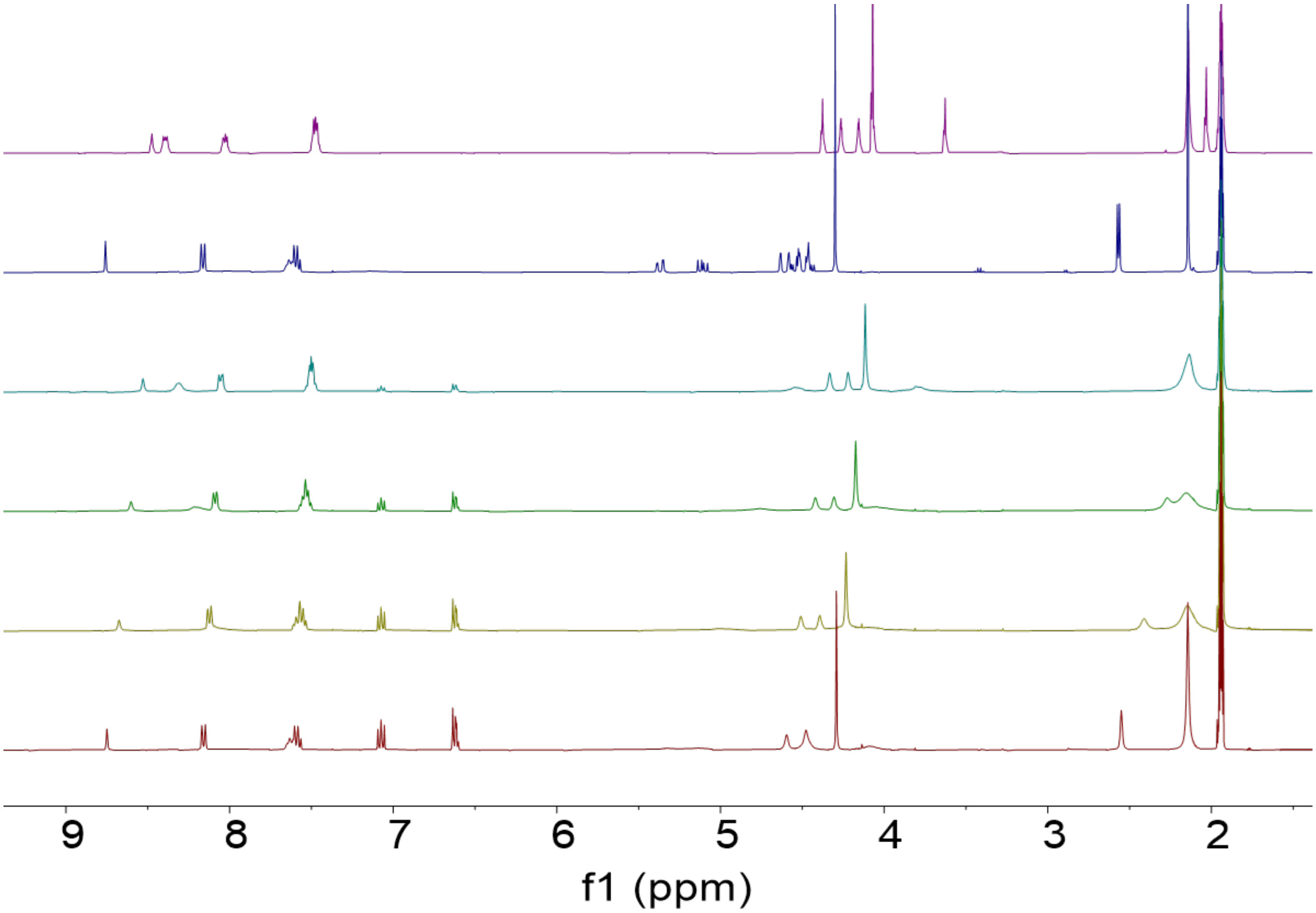

Fig. S15. ^{1}H NMR spectra (400 MHz) in CD_3CN for the titration experiment used in the pK_a determination of **{Fc–NH$^+$–an}**. The purple trace is the spectrum of **{Fc–N–an}**, the blue trace the spectrum of **{Fc–NH$^+$–an}**, and the cyan, green, yellow and red spectra correspond to subsequent additions of $[PhNH_3][OTf]$ to a CD_3CN solution of **{Fc–N–an}**.

S6. Stoichiometric photochemical reactions

General set up for a typical photochemical experiment: in a N_2 filled glove box, the corresponding mass of the **{Fc–N–an}** mediator, the [PicH][OTf] acid and the substrate was weighed and placed in a vial with a magnetic stir bar. Subsequently, 1 ml of dried and degassed THF was added and the vial was closed with a septum to ensure inert atmosphere. The vial was brought out of the glove box and irradiated with a 390 nm LED lamp during 1 h. Stirring and refrigeration with a fan was maintained throughout the experiment.

General work up methodology: after the reaction, the reaction vessel was open to air and 1 ml of HCl in diethyl ether was delivered. After stirring for 5 min, the mixture was evaporated to dryness. The remaining solids were extracted with 5 ml of diethyl ether, which was subsequently filtered, washed with concentrated solution of saturated NH_4Cl and water, dried over sodium sulfate and evaporated to dryness. Subsequently, 1 ml of $CDCl_3$ and 5 μL of CH_2Br_2 as internal standard were added and ^1H NMR was performed to analyze for product formation.

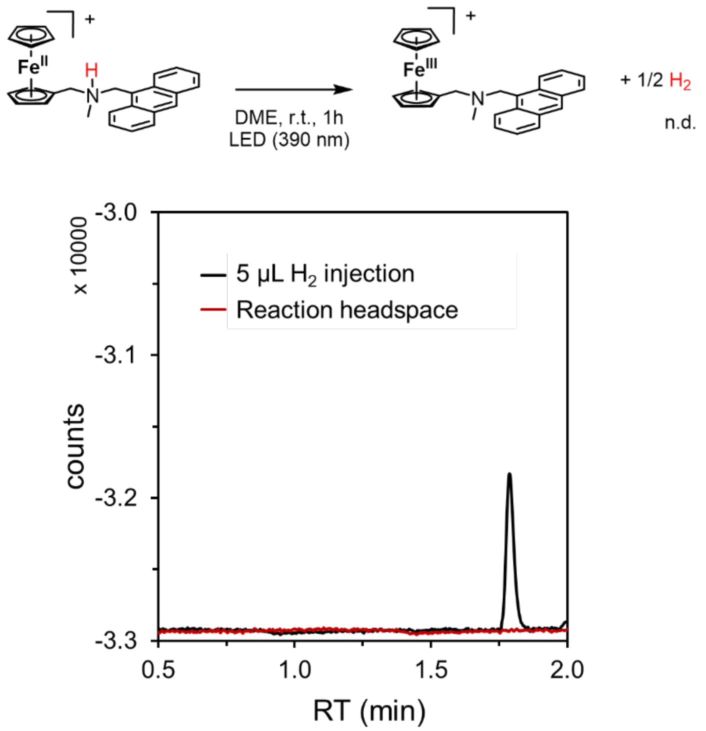

Fig. S16. Schematic representation of the photochemical reaction of 2 mM **{Fc–NH$^+$–an}** in DME upon irradiation with a 390 nm LED lamp for 1 h at room temperature to test the potential formation of H_2 under photochemical conditions. The graph represents the GC-TCD trace of the headspace analyzed after the reaction (red trace) and the injection of 5μL of H_2 as a standard (corresponding to approximately the maximum amount of H_2 from the headspace detected in a 100 μL injection).

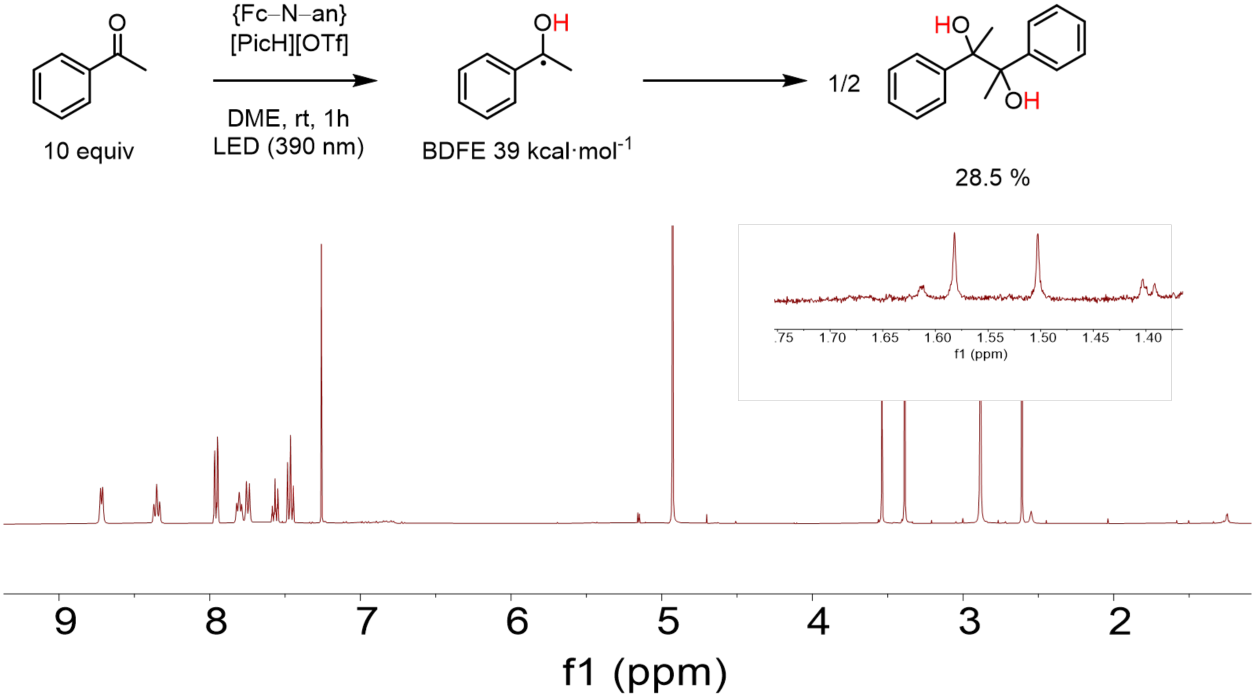

Fig. S17. Schematic representation of the photochemical reaction of 1 mM **{Fc–N–an}** in DME containing 10 mM acetophenone and 10 mM [PicH][OTf] upon irradiation with a 390 nm LED lamp for 1 h at room temperature, and the associated ^{1}H NMR spectra of the reaction after work up. This reaction was further confirmed but GC-FID and GC-MS.

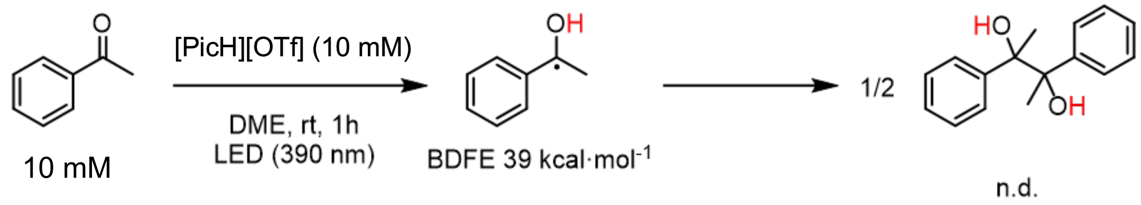

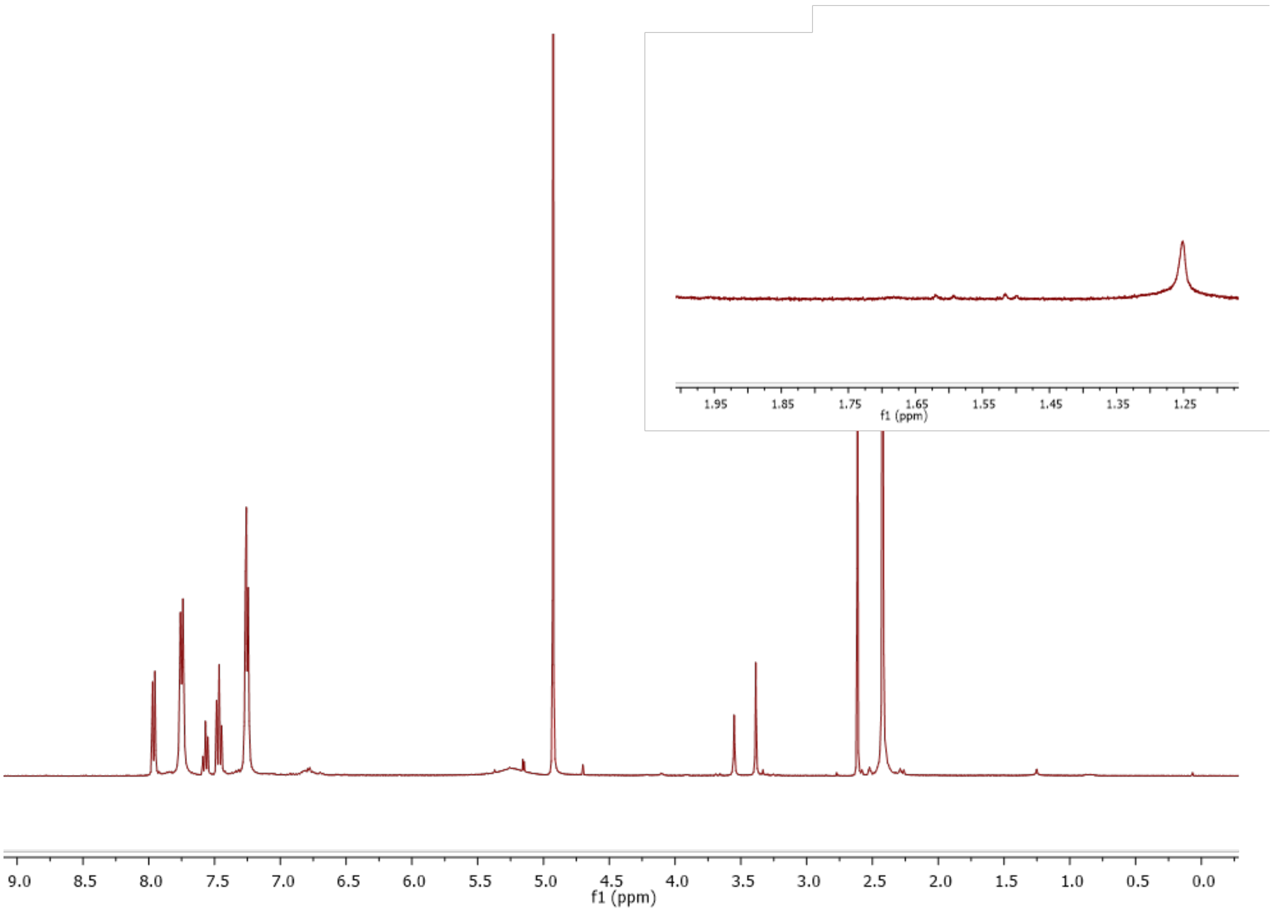

Fig. S18. Schematic representation of the photochemical control reaction without mediator in DME containing 10 mM [PicH][OTf] and 10 mM acetophenone upon irradiation with a 390 nm LED lamp for 1 h at room temperature, and the associated ^{1}H NMR spectra of the reaction after work up.

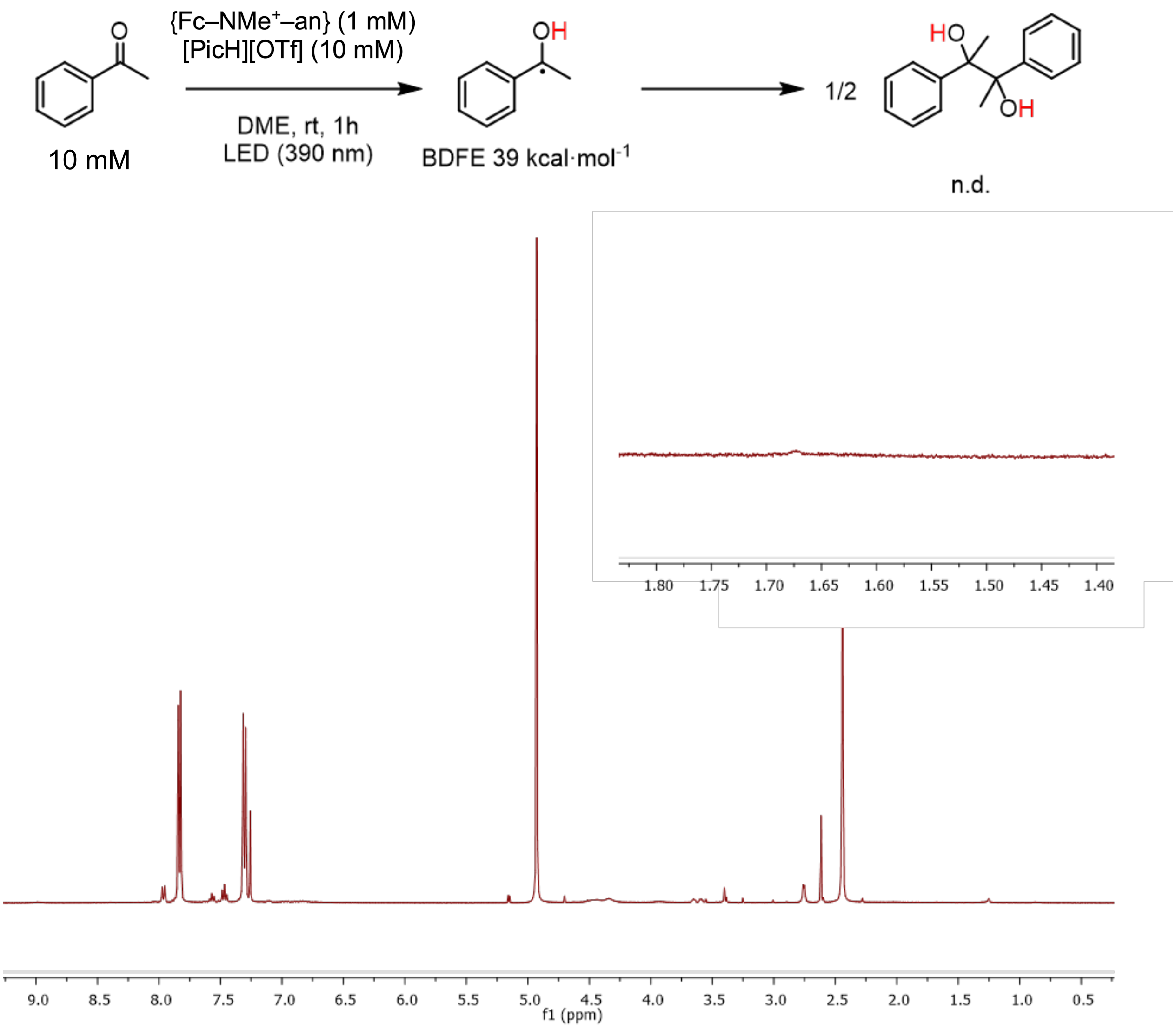

Fig. S19. Schematic representation of the photochemical control reaction of 1 mM **{Fc–NMe$^+$–an}** in DME containing 10 mM acetophenone and 10 mM [PicH][OTf] upon irradiation with a 390 nm LED lamp for 1 h at room temperature, and the associated ^{1}H NMR spectra of the reaction after work up. **{Fc–NMe$^+$–an}** was generated in situ by addition of 1 equivalent of MeOTf and stirred during 10 mins. Immediate change in color to a more intense yellow indicated the formation of the targeted species. This process was also independently monitored by UV-vis to confirm quantitative formation of {Fc–NMe$^+$–an}. This experiment supports that protonation at the N atom in the amine group is required for the photochemical PCET reaction, disfavoring a multicomponent process.

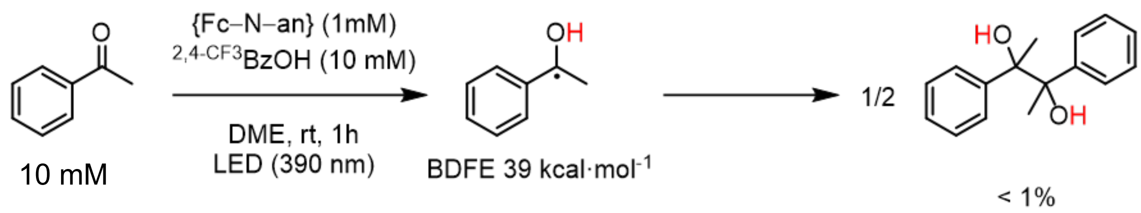

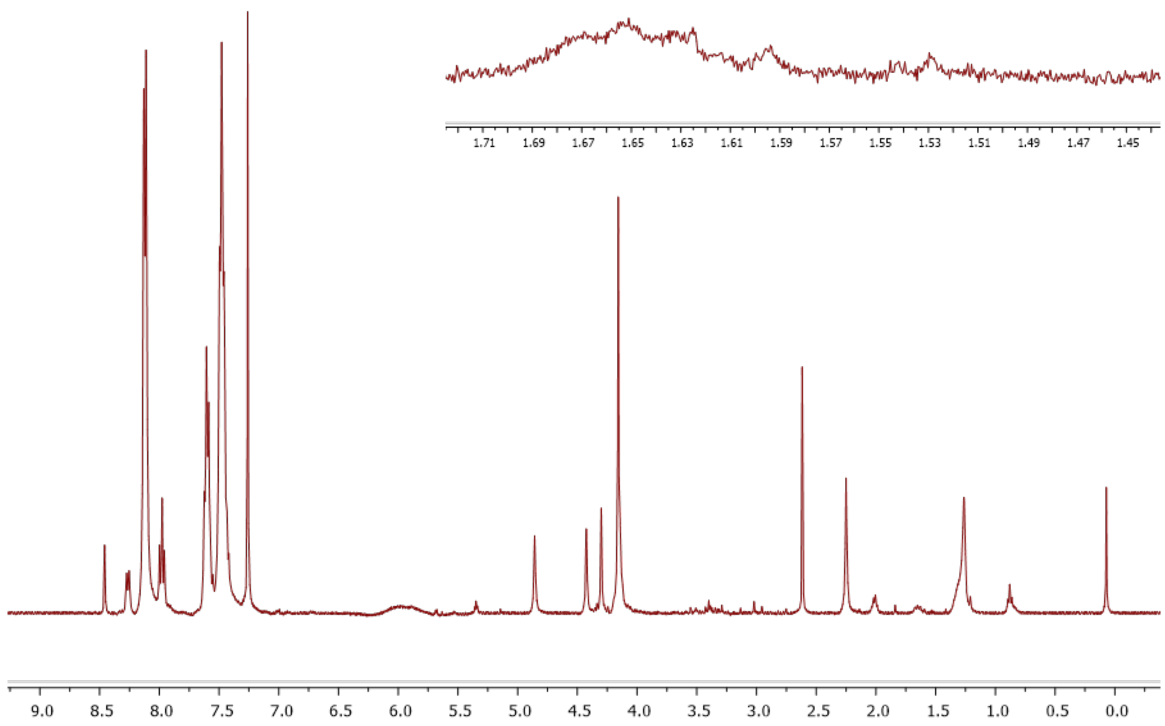

Fig. S20. Schematic representation of the photochemical control reaction of 1 mM **{Fc–N–an}** in DME containing 10 mM Acetophenone and 10 mM $^{2,4\text{-}CF_3}$BzOH upon irradiation with a 390 nm LED lamp for 1 h at room temperature, and the associated ^{1}H NMR spectra of the reaction after work up. This experiment supports that protonation at the N atom in the amine group is required for the photochemical PCET reaction, disfavoring a multicomponent process.

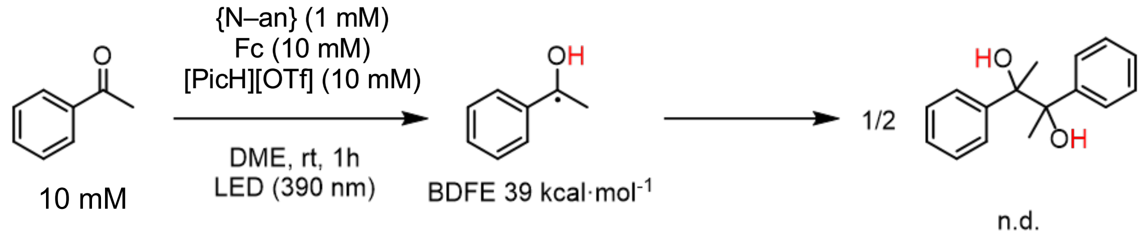

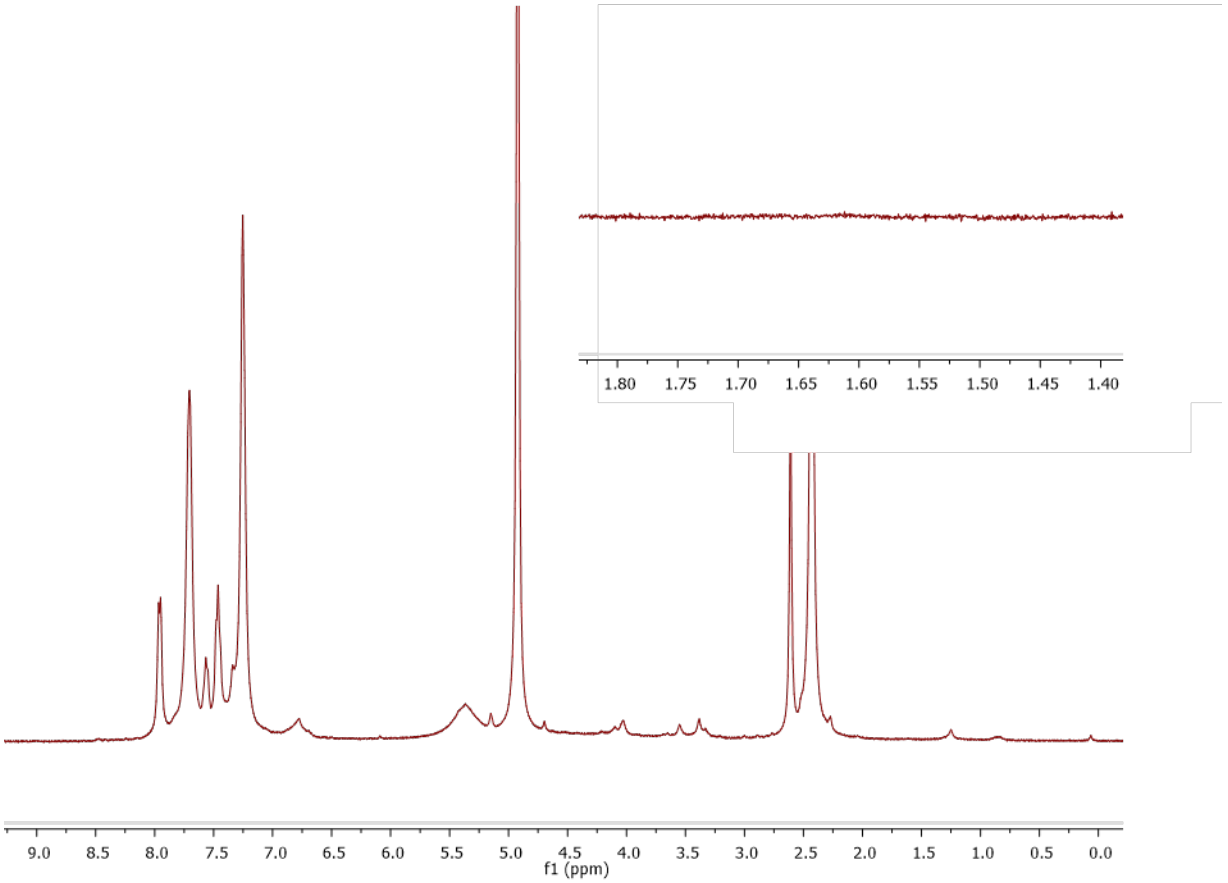

Fig. S21. Schematic representation of the photochemical control reaction of 1 mM **{N–an}** in DME containing 10 mM Fc, 10 mM Acetophenone and 10 mM [PicH][OTf] upon irradiation with a 390 nm LED lamp for 1 h at room temperature, and the associated ^{1}H NMR spectra of the reaction after work up. This experiment supports the need for the synthetic integration of the electron donor (Fc), the Brønsted base (amine group) and the dye (anthracene) for effective PCET reaction.

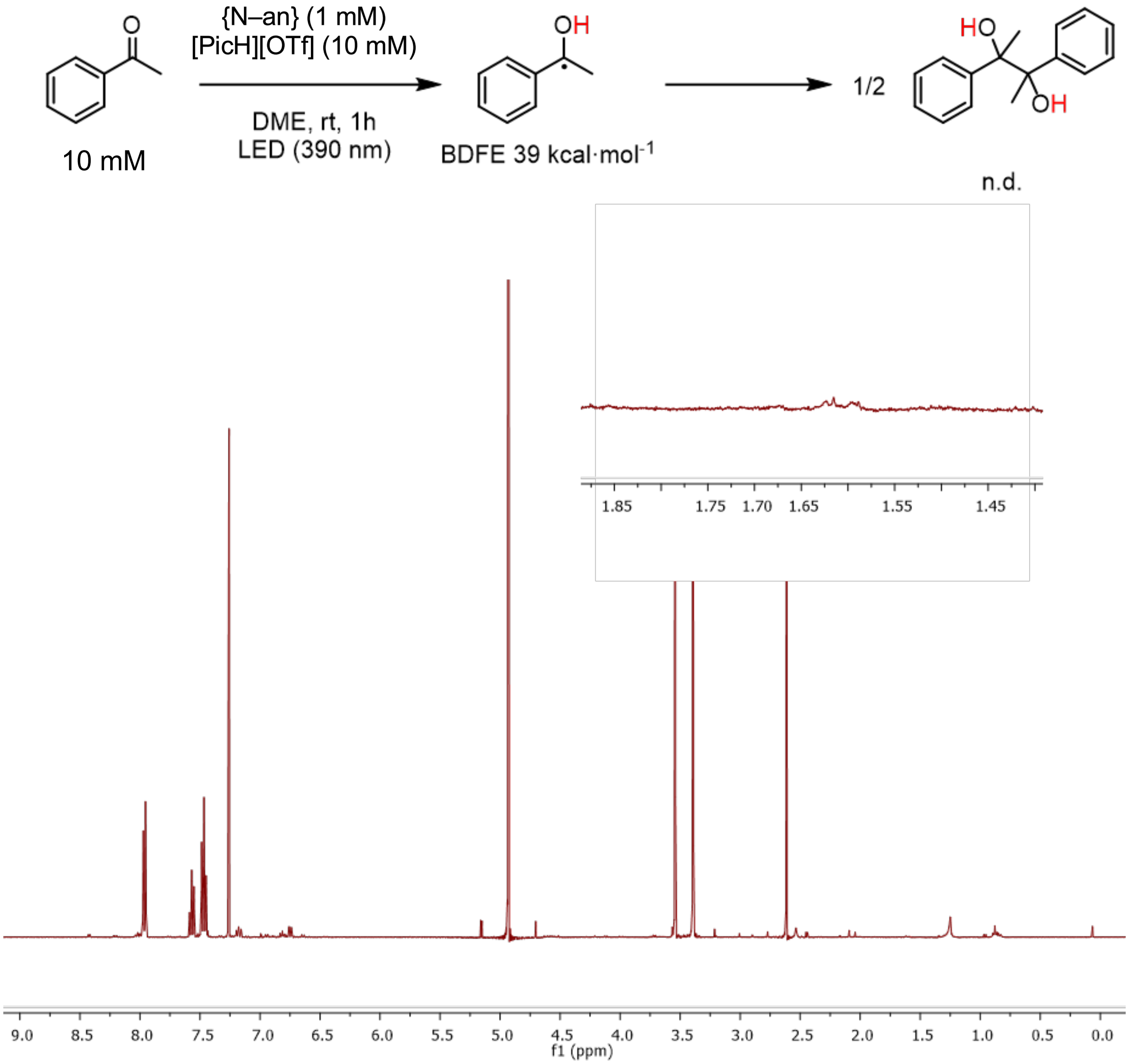

Fig. S22. Schematic representation of the photochemical control reaction of 1 mM AntN in DME containing 10 mM Acetophenone and 10 mM [PicH][OTf] upon irradiation with a 390 nm LED lamp for 1 h at room temperature, and the associated ^{1}H NMR spectra of the reaction after work up. This experiment supports the need for the synthetic integration of the electron donor (Fc), the Brønsted base (amine group) and the dye (anthracene) for effective PCET reaction.

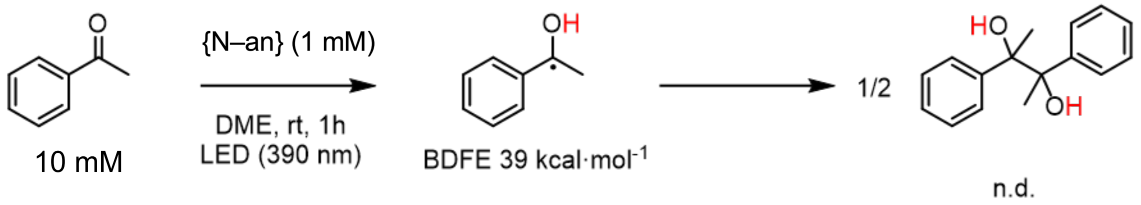

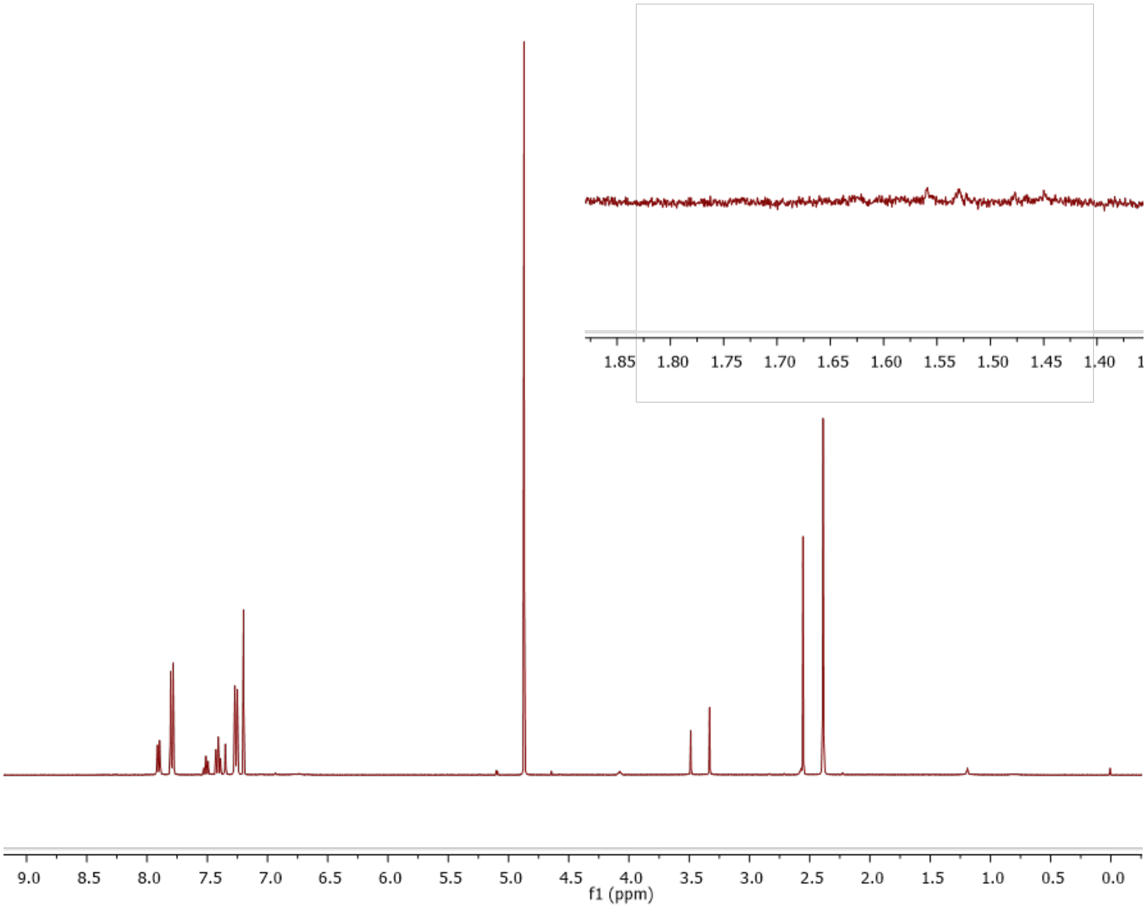

Fig. S23. Schematic representation of the photochemical control reaction of 1 mM [AntNH][OTf] in DME containing 10 mM Acetophenone and 10 mM [PicH][OTf] upon irradiation with a 390 nm LED lamp for 1 h at room temperature, and the associated ^{1}H NMR spectra of the reaction after work up. This experiment supports the need for the synthetic integration of the electron donor (Fc), the Brønsted base (amine group) and the dye (anthracene) for effective PCET reaction.

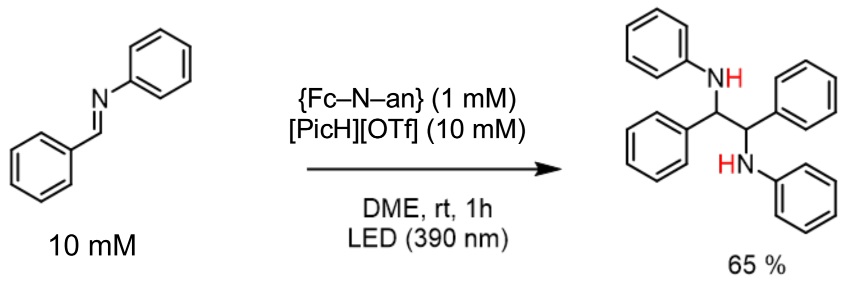

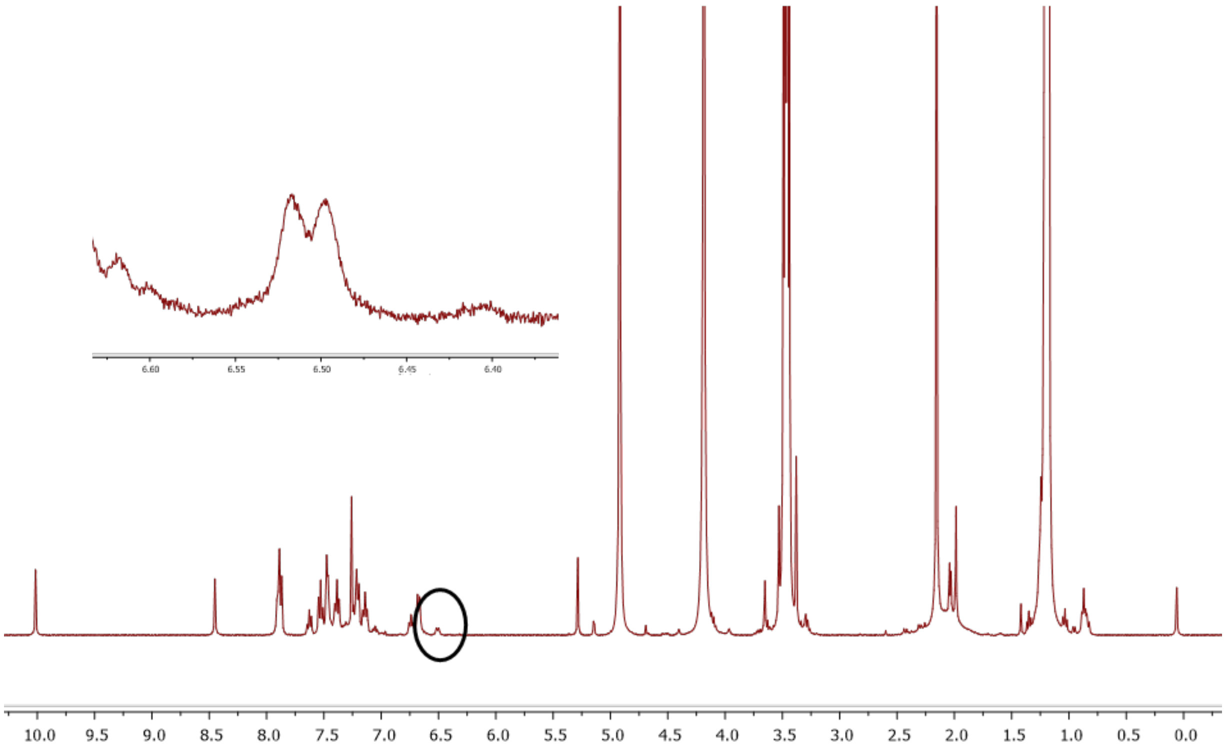

Fig. S24. Schematic representation of the photochemical reaction of 1 mM **{Fc–N–an}** in DME containing 10 mM phenylbenzilimine and 10 mM [PicH][OTf] upon irradiation with a 390 nm LED lamp for 1 h at room temperature, and the associated ^{1}H NMR spectra of the reaction after work up. The circled peak associated to the product were used for integration.

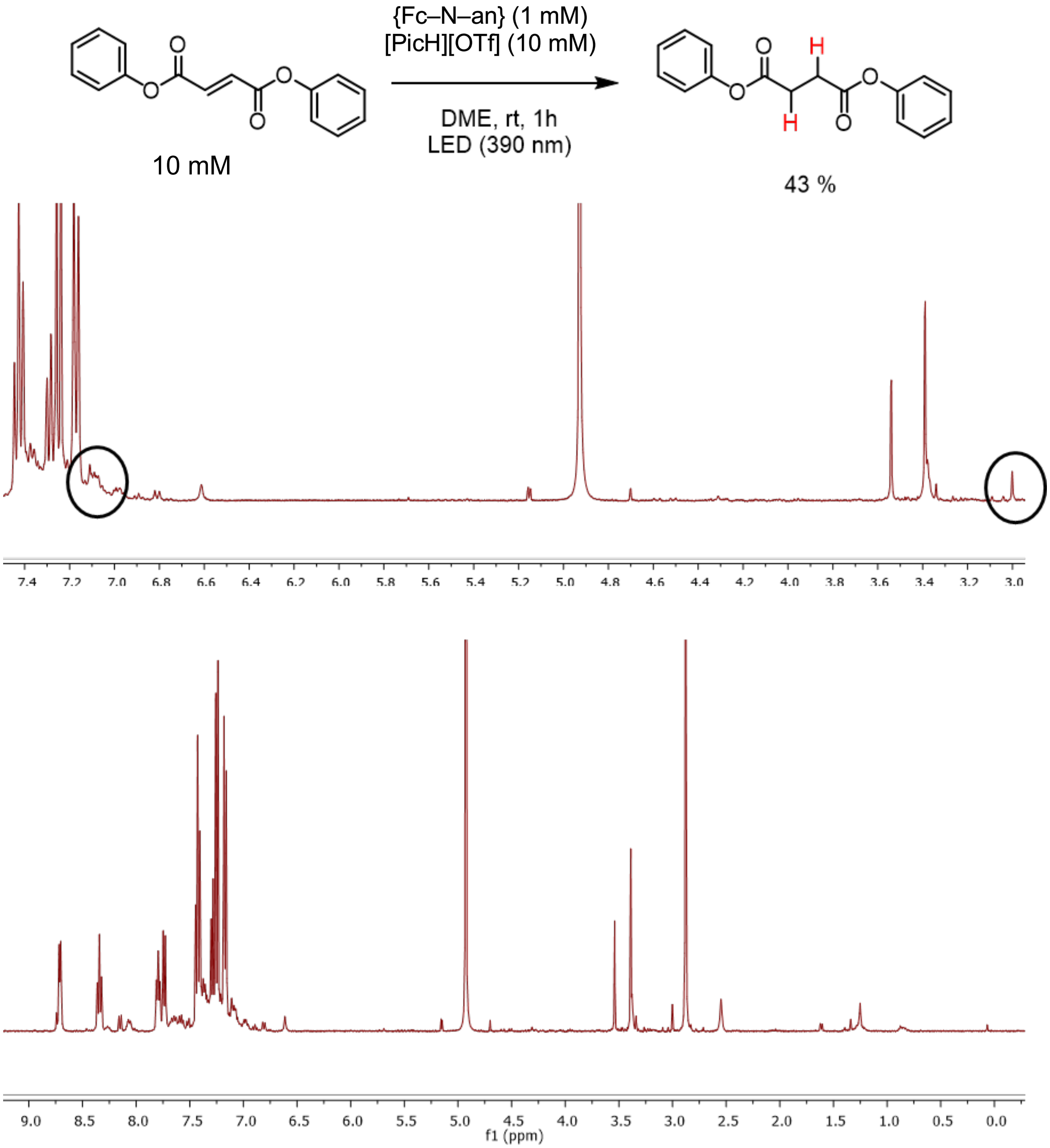

Fig. S25. Schematic representation of the photochemical reaction of 1 mM **{Fc–N–an}** in DME containing 10 mM diphenylfumarate and 10 mM [PicH][OTf] upon irradiation with a 390 nm LED lamp for 1 h at room temperature, and the associated ^{1}H NMR spectra of the reaction after work up. The circled peaks associated to the product were used for integration.

S7. Fluorescence data

I. Fluorescence Spectra

Steady-state fluorescence spectra were measured for several of the relevant compounds to determine properties of the singlet excited **^{1}an***. All spectra were recorded after excitation at 355 nm, with sample concentrations of 0.16 mM in anhydrous DME, prepared in an N_2 filled glovebox.

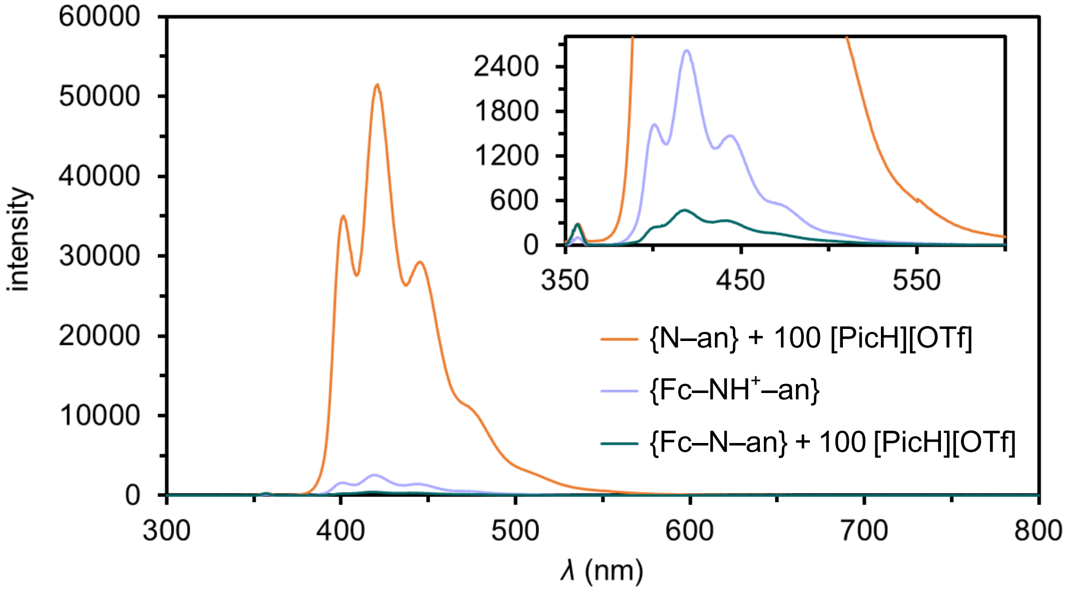

Fig. S26. Steady-state fluorescence spectra of the protonated organic model complex **{NH^{+}–an}** and the complex **{Fc–NH^{+}–an}** with and without excess [PicH][OTf]. A quantum yield analysis (comparing the areas under each curve) reveals that **{Fc–NH^{+}–an}** is effectively quenched 95 % without excess [PicH][OTf] and 99 % with 100 equivalents [PicH][OTf] relative to the unquenched **{NH^{+}–an}**.

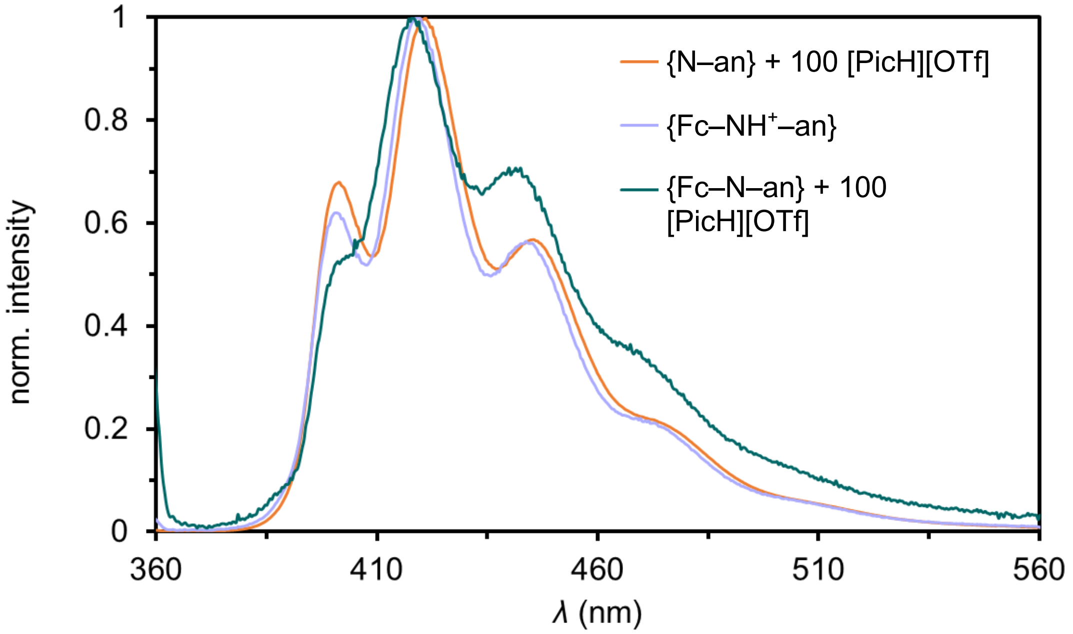

Fig. S27. Normalized steady-state fluorescence spectra of the protonated organic model complex **{NH$^+$–an}** to the complex **{Fc–NH$^+$–an}** with and without excess [PicH][OTf].

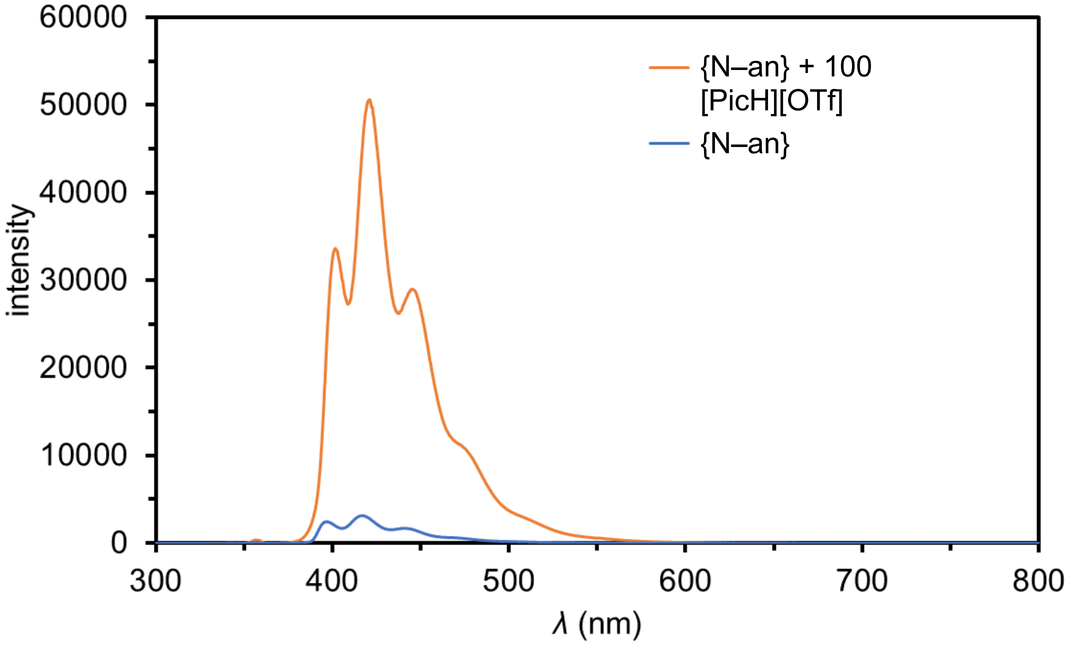

Fig. S28. Steady-state fluorescence spectra of the protonated **{NH$^+$–an}** vs unprotonated **{N–an}** organic model complex.

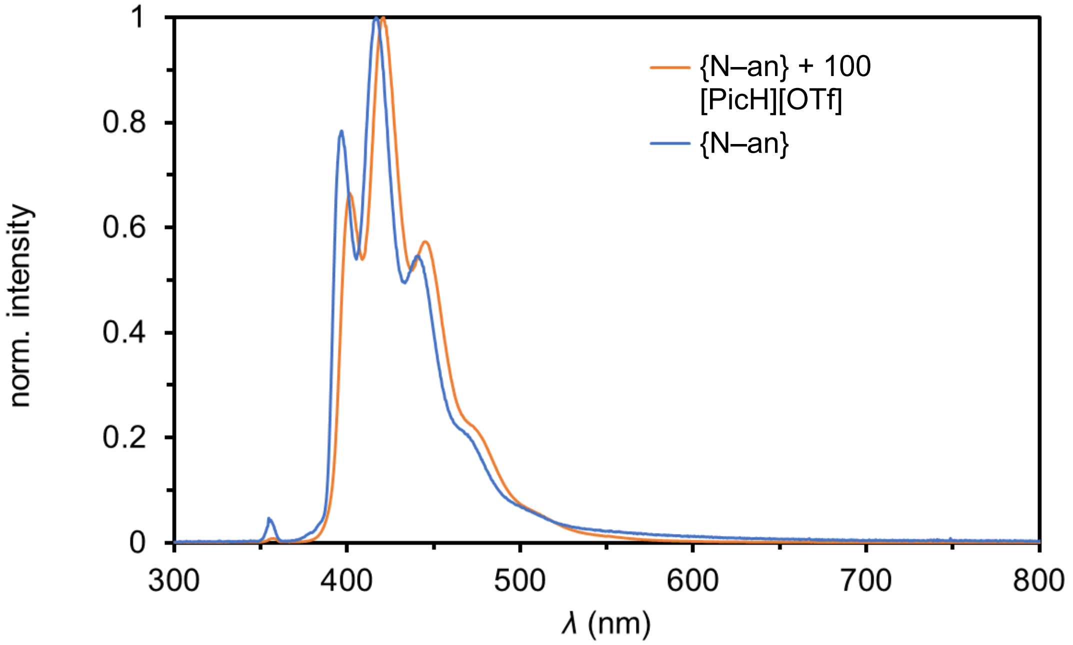

Fig. S29. Normalized steady-state fluorescence spectra of **{N–an}** and **{NH⁺–an}**. The 238 cm^{-1} red shift in the spectrum of **{NH⁺–an}** is consistent with a decrease of 0.5 in the **NH⁺** pK_a for **{NH⁺–^{1*}an}** relative to **{NH⁺–an}**.

II. Time-Resolved Fluorescence Data

Time-resolved fluorescence measurements were carried out under an N_2 atmosphere in sealed, air-tight cuvettes. All samples had a concentration of 0.16 mM photoactive species (**{Fc–N–an}** or **{N–an}**) with 100 equivalents [PicH][OTf] in DME.

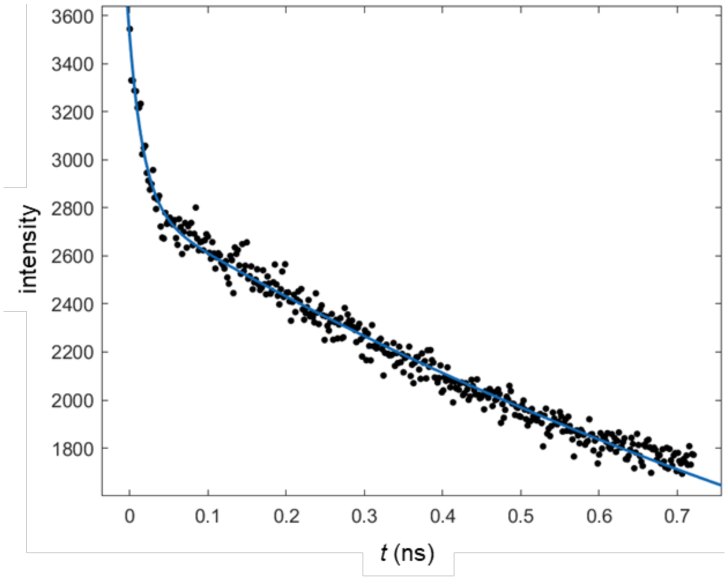

Fig. S30. Fluorescence decay kinetics of **{Fc–NH$^+$–an}** in DME in the presence of 100 equivalents [PicH][OTf]. Fitted to a bi-exponential, the first decay has a lifetime of 18 ± 2 ps, which we assign to the quenching of the singlet **^{1}an*** by the tethered Fc unit. The slower decay can be attributed to the decay of the unquenched singlet **^{1}an*** ($y = 710.3\ e^{-53.9x} + 2797\ e^{-0.701x}$; $R^2 = 0.98$).

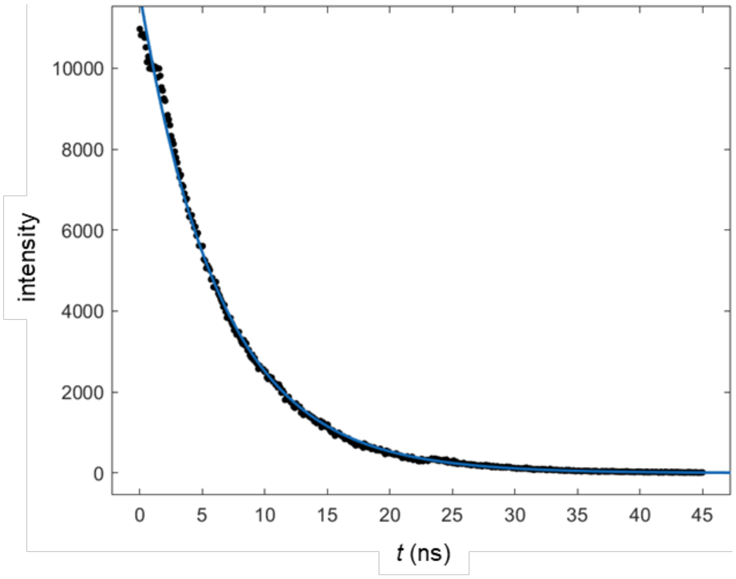

Fig. S31. Exponential fit for transient fluorescence data of **{NH$^+$–an}** in DME in the presence of 100 equivalents [PicH][OTf]. Fitted to a single exponential, it decays with a lifetime of 6.45 ± 0.05 ns as the unquenched singlet lifetime of **^{1}an***. ($y = 11830\ e^{-0.155x}$; $R^2 = 0.99$)

S8. Transient absorption data

Transient absorption measurements were carried out under an N_2 atmosphere in sealed, air-tight cuvettes. All samples had a concentration of 0.16 mM analyte (**{Fc–N–an}**, **{Fc–NH$^+$–an}**, or {N–an}) with 100 equivalents [PicH][OTf] and varying concentrations of acetophenone (quencher) in DME at room temperature. For all kinetic data, rates were extracted from exponentially fitting the raw data, and error in this fit is expressed through the standard deviation associated with the rate constant.

I. Lifetime of **{Fc$^+$–NH$^+$–an$^{•–}$}** in the presence of excess acid

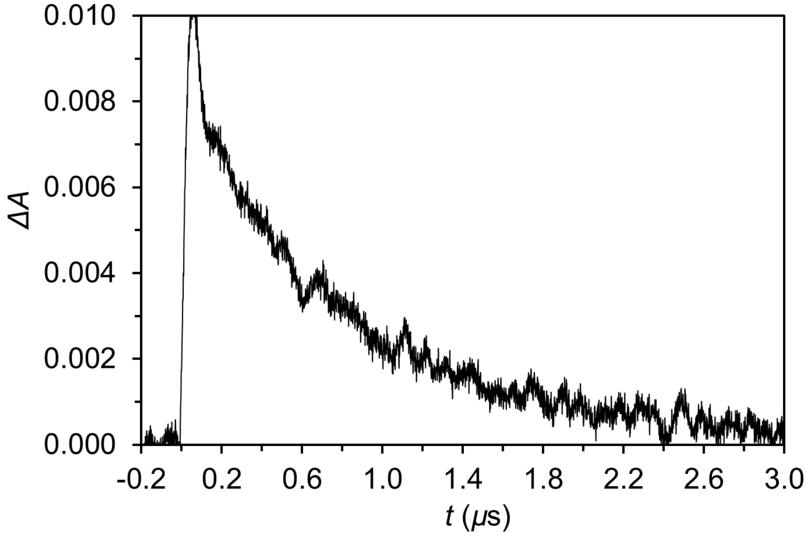

Fig. S32. Time-resolved transient absorption measurement of sample with **{Fc–N–an}** and 100 equivalents [PicH][OTf] in DME monitoring the decay of a signal at 700 nm, assigned to the anthracene radical anion formed from the reductive quenching of the **^{1}an*** excited state by Fc. Exponential fit of the data gives a lifetime of 0.87 ± 0.2 μs from duplicate measurements.

We observed that the transient species at 700 nm had a more intense signal and a longer lifetime in the presence of excess [PicH][OTf] compared with **{Fc–NH$^+$–an}** generated by protonation with 1 equivalent of HOTf. We theorize that this is a charge stabilization effect, and the excess acid acts to increase the polarity of the medium, which stabilizes the charge separated state.

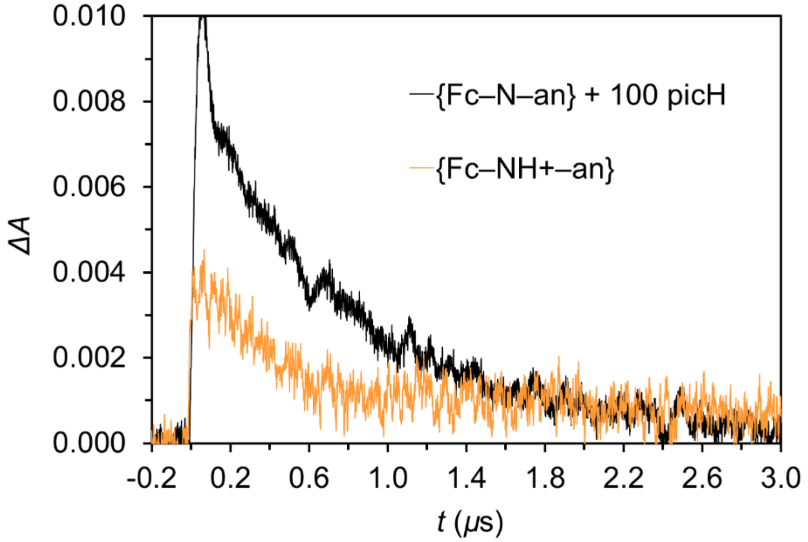

Fig. S33. Time-resolved transient absorption measurement of sample with **{Fc–NH$^+$–an}** in DME monitoring the decay of a signal at 700 nm. Under these conditions, this species has a lifetime of 0.66 ± 0.01 μs from duplicate measurements.

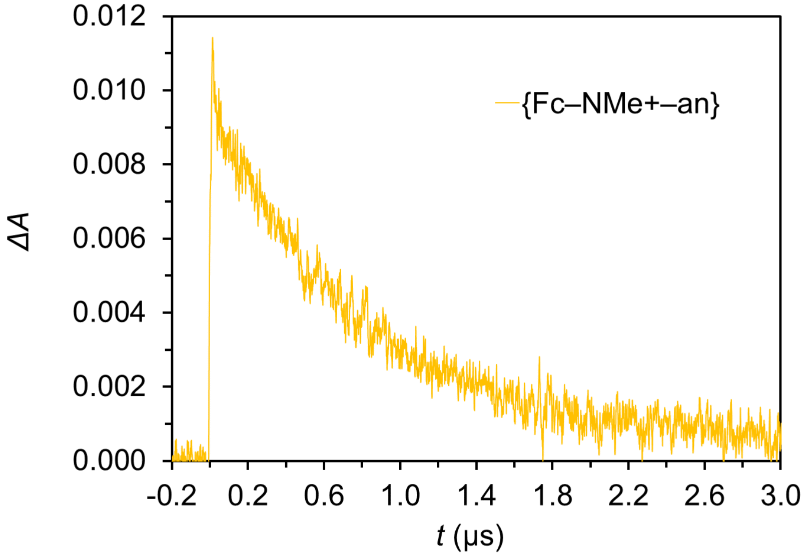

Fig. S34. Time-resolved transient absorption measurement of sample with **{Fc–NMe$^+$–an}** and 100 equivalents [PicH][OTf] in DME monitoring the decay of a signal at 700 nm. Under these conditions, this species has a lifetime of 0.887 ± 0.003 μs, indicating the formation of **{Fc$^+$–NMe$^+$–an$^{•-}$}**.

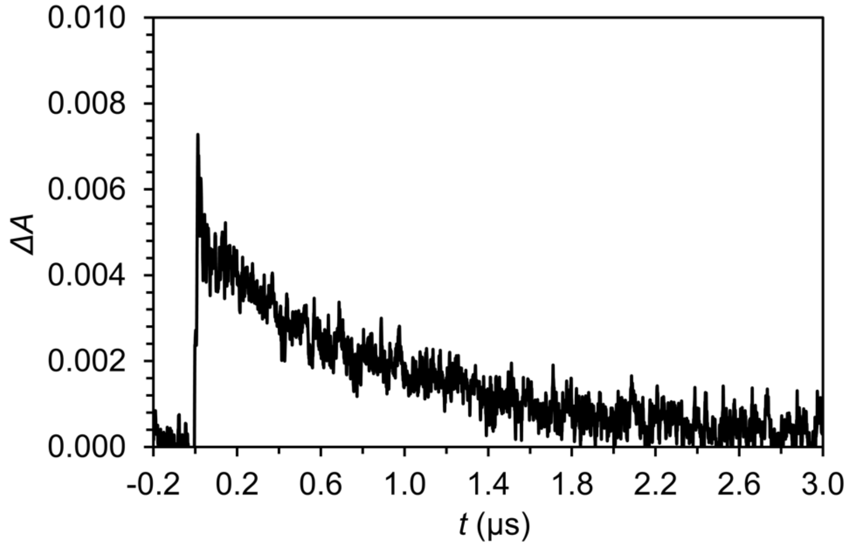

Figure S35. Time-resolved transient absorption measurement of sample with **{Fc–NH$^+$–an}** and 32 mM [PicH][OTf] in DME monitoring the decay of a signal at 700 nm. Under these conditions, this species has a lifetime of 1.00 ± 0.01 μs.

We hypothesized that electrolytes other than [PicH][OTf] could act to stabilize the CSS. We therefore interrogated **{Fc–NH$^+$–an}** by time-resolved TA spectroscopy in the presence of [TBA][OTf]. Under these conditions, the 700 nm transient has an increased lifetime of 1.33 μs, which we attribute to better charge stabilization by [TBA][OTf].

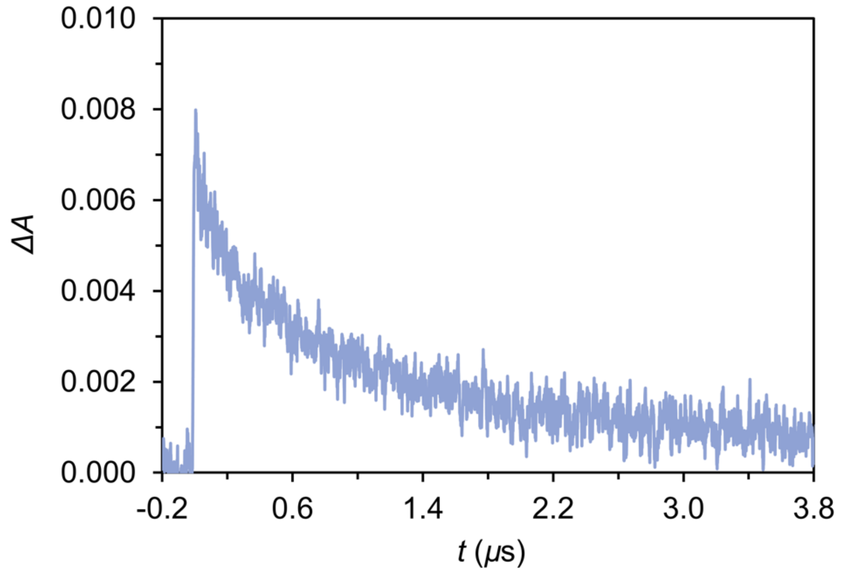

Figure S36. Time-resolved transient absorption measurement of sample with **{Fc–NH$^+$–an}** and 15 mM [TBA][OTf] in DME monitoring the decay of a signal at 700 nm. Under these conditions, this species has a lifetime of 1.33 ± 0.01 μs.

We also wondered if a neutral acid of similar pK_a to [PicH][OTf] would stabilize the CSS in the same way, which would point to a role of [PicH][OTf] also as a proton source upon charge separation instead of as an electrolyte. To test this, we employed 15 mM $HNTs_2$ (pK_a (MeCN) = 12) and saw a significantly decreased lifetime of the 700 nm transient as compared to the same concentration of [PicH][OTf].

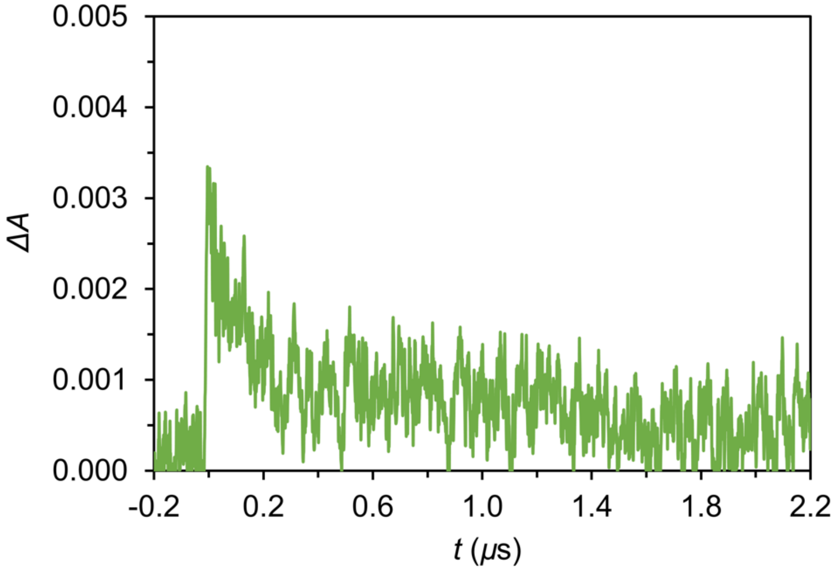

Figure S37. Time-resolved transient absorption measurement of sample with **{Fc–NH$^+$–an}** and 100 equivalents $HNTs_2$ in DME monitoring the decay of a signal at 700 nm. Under these conditions, this species has a lifetime of 0.15 ± 0.01 μs.

II. Stern-Volmer quenching experiment with acetophenone (logarithmically compressed data overlays full dataset)

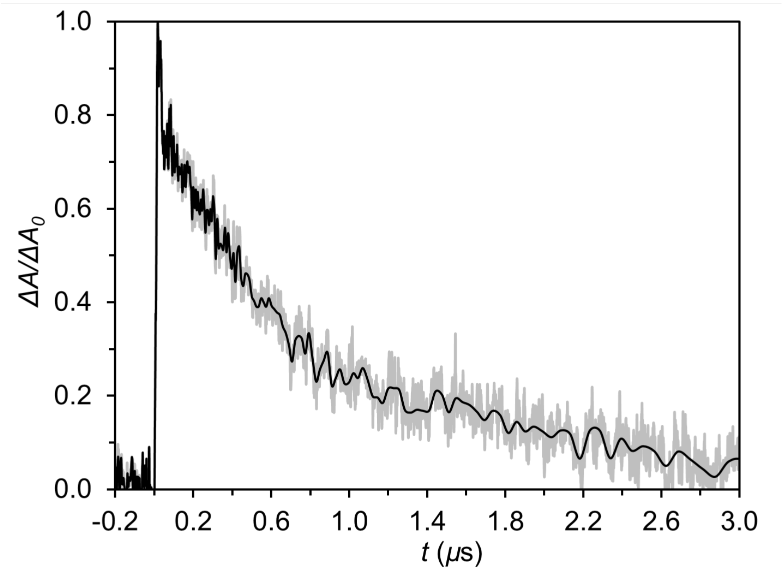

Fig. S38. Time-resolved transient absorption measurement of sample with **{Fc–N–an}** and 100 equivalents [PicH][OTf] in DME monitoring the decay of a signal at 700 nm. An exponential fit of the data reveals an observed rate constant of $1.13 \pm 0.02 \times 10^6$ s^{-1} from duplicate measurements.

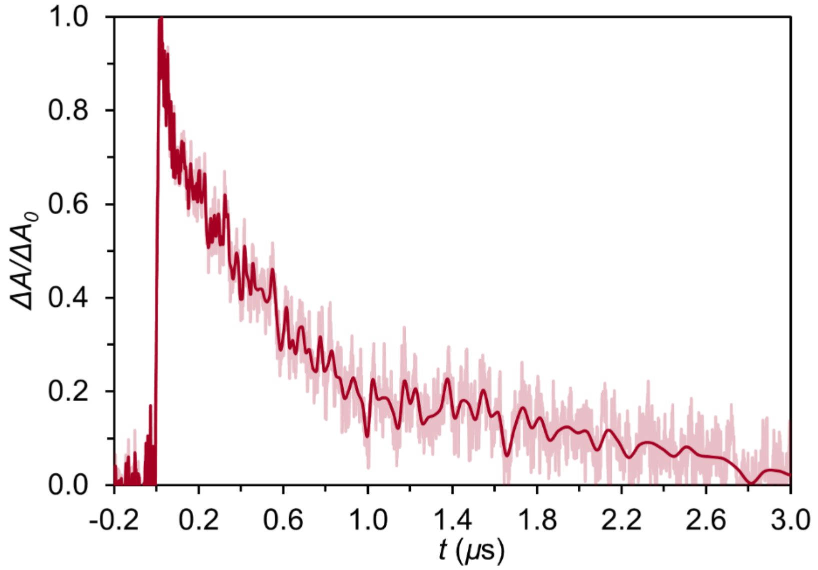

Fig. S39. Time-resolved transient absorption measurement of sample with **{Fc–N–an}**, 100 equivalents [PicH][OTf], and 3.13 mM acetophenone in DME monitoring the decay of a signal at 700 nm. An exponential fit of the data reveals an observed rate constant of $1.29 \pm 0.01 \times 10^6$ s^{-1}.

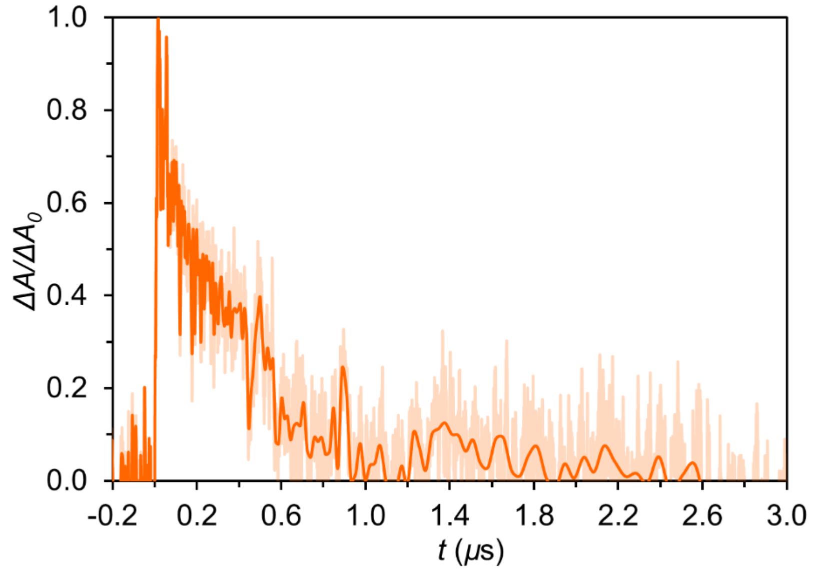

Fig. S40. Time-resolved transient absorption measurement of sample with **{Fc–N–an}**, 100 equivalents [PicH][OTf], and 6.25 mM acetophenone in DME monitoring the decay of a signal at 700 nm. An exponential fit of the data reveals an observed rate constant of $1.58 \pm 0.04 \times 10^6$ s^{-1}.

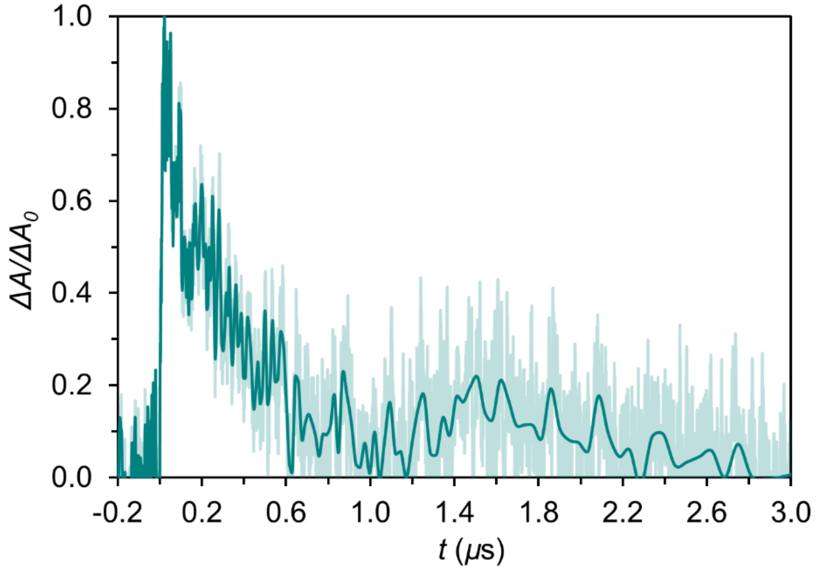

Fig. S41. Time-resolved transient absorption measurement of sample with **{Fc–N–an}**, 100 equivalents [PicH][OTf], and 12.5 mM acetophenone in DME monitoring the decay of a signal at 700 nm. An exponential fit of the data reveals an observed rate constant of $1.89 \pm 0.06 \times 10^6$ s^{-1}.

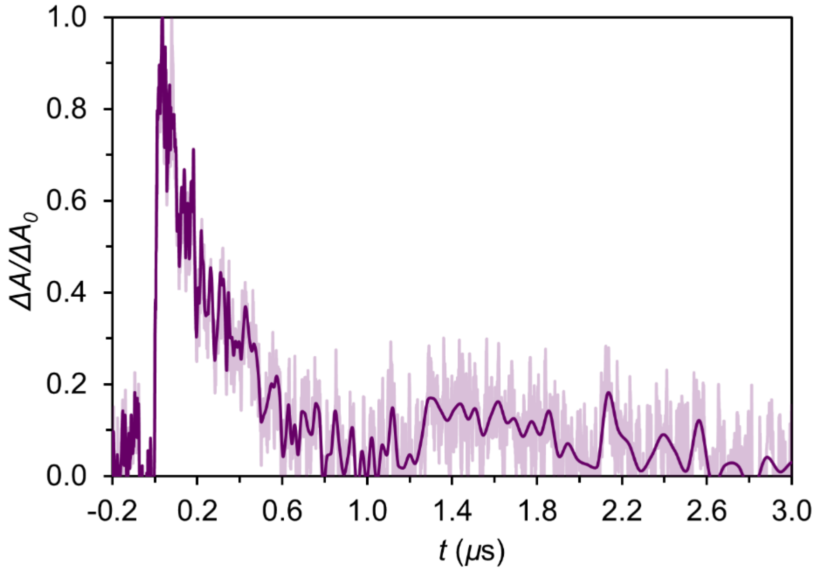

Fig. S42. Time-resolved transient absorption measurement of sample with **{Fc–N–an}**, 100 equivalents [PicH][OTf], and 18.8 mM acetophenone in DME monitoring the decay of a signal at 700 nm. An exponential fit of the data reveals an observed rate constant of $2.90 \pm 0.08 \times 10\ s^{-1}$.

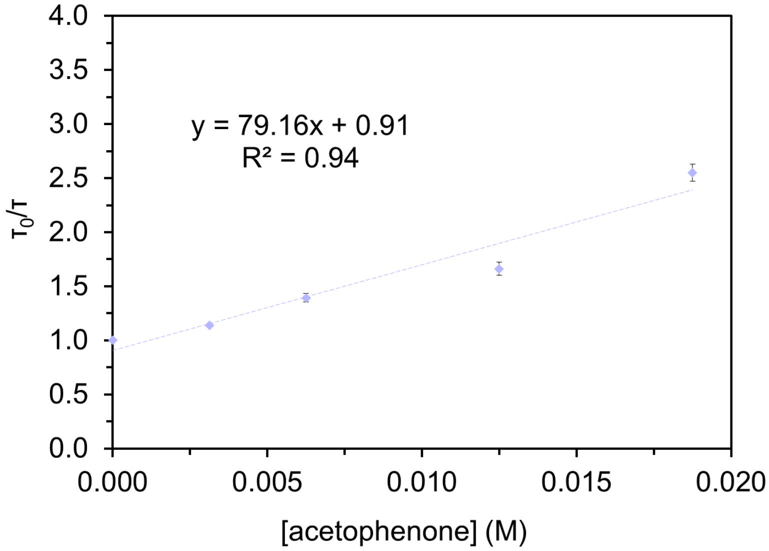

Fig. S43. Stern-Volmer quenching analysis of with relative lifetimes of **{Fc^+–NH^+–$an^{\bullet-}$}** in the presence of varying concentrations of acetophenone. Lifetimes were obtained from exponential fits of the data in section S8.V.

a. Determination of a kinetic isotope effect *via* transient absorption

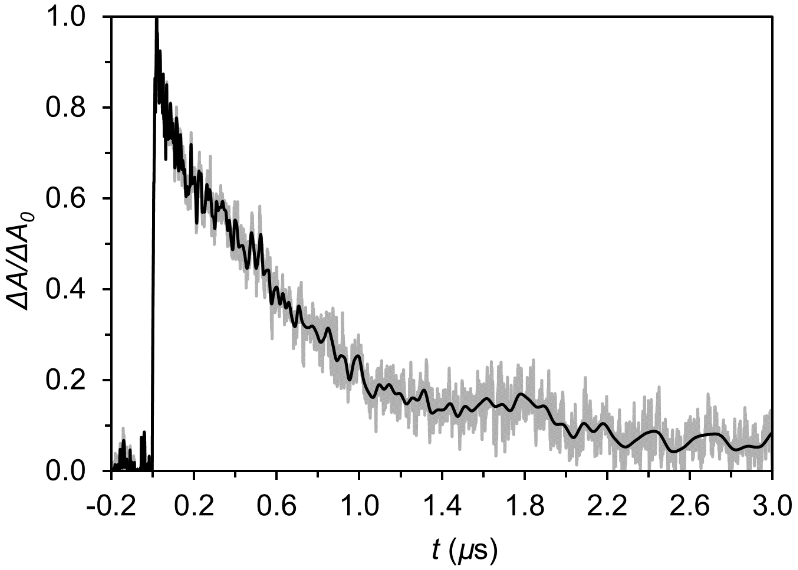

Fig. S44. Time-resolved transient absorption measurement of sample with **{Fc–N–an}** and 100 equivalents PicDOTf in DME monitoring the decay of a signal at 700 nm. An exponential fit of the data reveals an observed rate constant of 1.212 ± 0.009 × 10^6 s^{-1}.

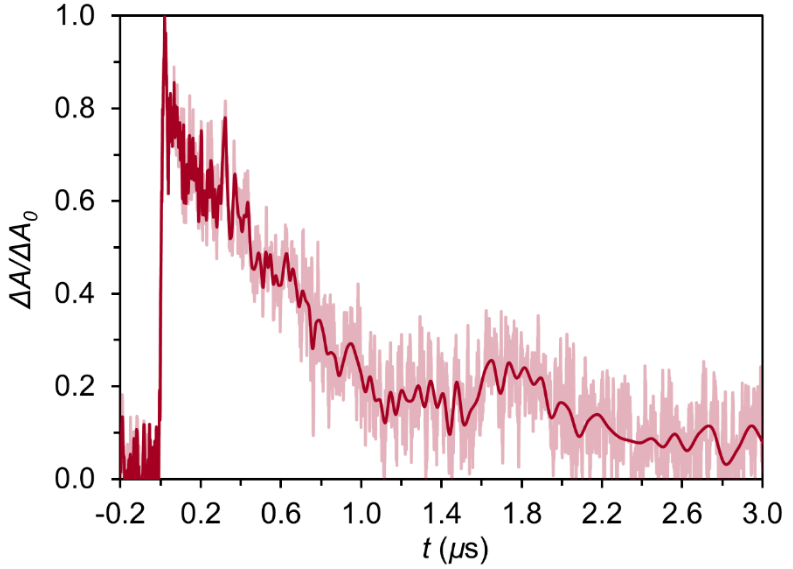

Fig. S45. Time-resolved transient absorption measurement of sample with **{Fc–N–an}**, 100 equivalents PicDOTf, and 3.13 mM acetophenone in DME monitoring the decay of a signal at 700 nm. An exponential fit of the data reveals an observed rate constant of 1.30 ± 0.02 × 10^6 s^{-1}.

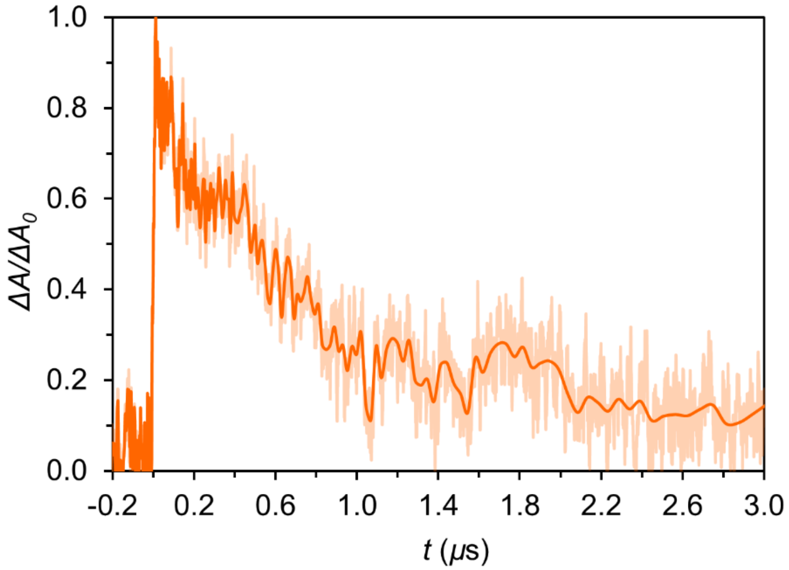

Fig. S46. Time-resolved transient absorption measurement of sample with **{Fc–N–an}**, 100 equivalents PicDOTf, and 6.25 mM acetophenone in DME monitoring the decay of a signal at 700 nm. An exponential fit of the data reveals an observed rate constant of $1.39 \pm 0.01 \times 10^6$ s^{-1}.

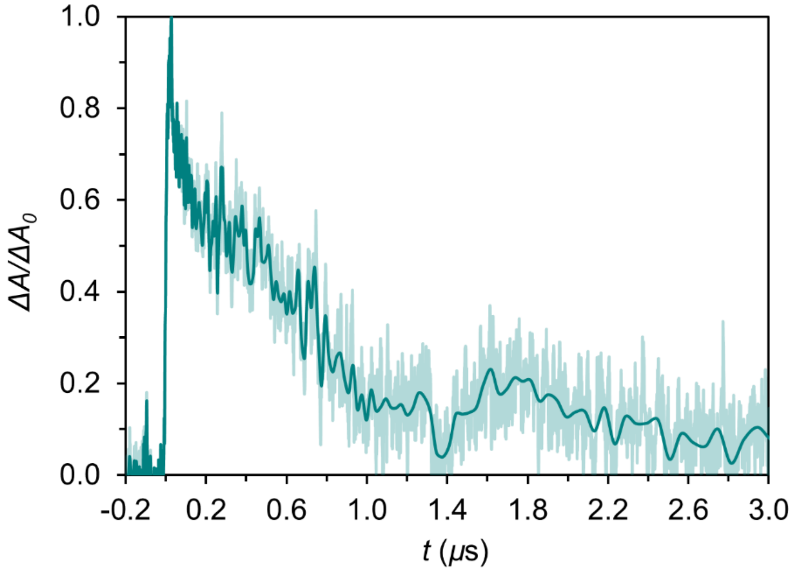

Fig. S47. Time-resolved transient absorption measurement of sample with **{Fc–N–an}**, 100 equivalents PicDOTf, and 12.5 mM acetophenone in DME monitoring the decay of a signal at 700 nm. An exponential fit of the data reveals an observed rate constant of $1.49 \pm 0.02 \times 10^6$ s^{-1}.

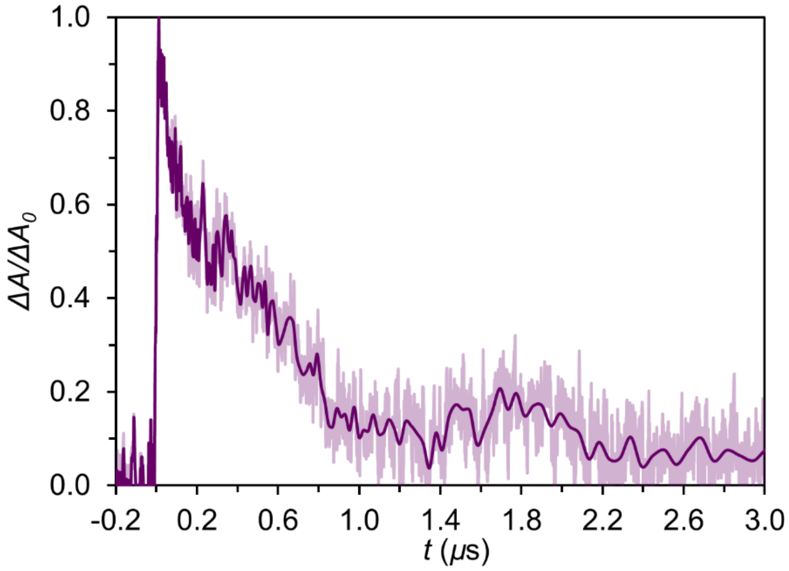

Fig. S48. Time-resolved transient absorption measurement of sample with **{Fc–N–an}**, 100 equivalents PicDOTf, and 18.8 mM acetophenone in DME monitoring the decay of a signal at 700 nm. An exponential fit of the data reveals an observed rate constant of $1.86 \pm 0.03 \times 10^{6}$ s^{-1}.

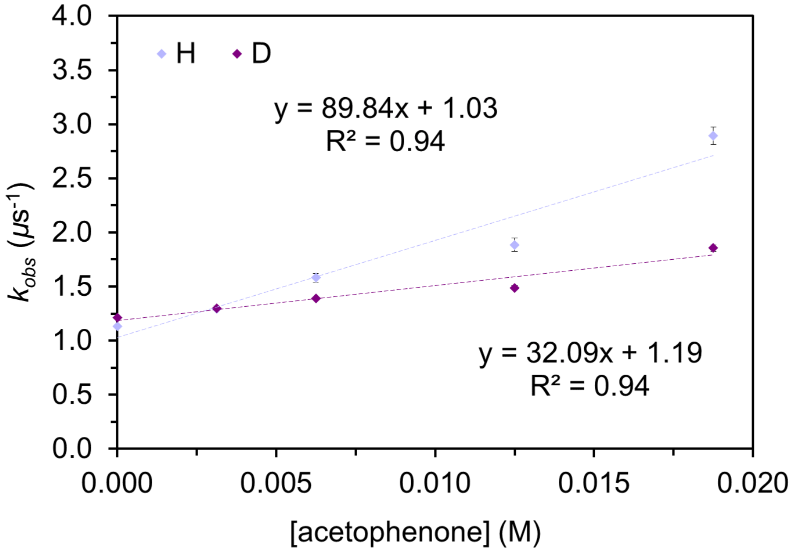

Fig. S49. Comparison of rates derived *via* a Stern-Volmer quenching analysis for excited **{Fc–N–an}** in the presence of varying concentrations of acetophenone and [PicH][OTf] (blue) or PicDOTf (purple). This gives a rate of ${}^{H}k_{PCET} = 9.0 \pm 1.3 \times 10^{7}$ M^{-1} s^{-1} and ${}^{D}k_{PCET} = 3.2 \pm 0.5 \times 10^{7}$ M^{-1} s^{-1}

$$KIE = \frac{k_H}{k_D} \quad \text{Eq. S3}$$

With these data, a KIE of 2.8 ± 0.4 is obtained for PCET from **{Fc^{+}–NH^{+}–an$^{\bullet-}$}** to acetophenone.

III. Acid dependence

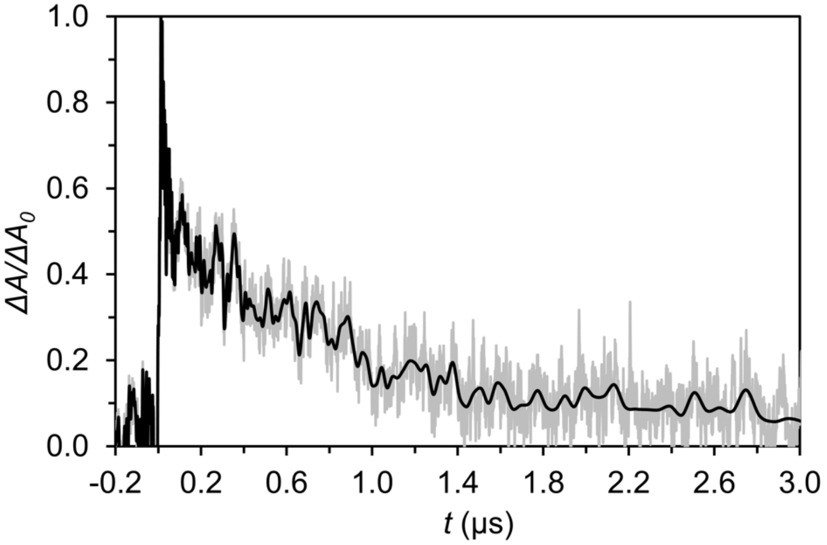

Fig. S50. Time-resolved transient absorption measurement of sample with **{Fc–N–an}**, 120 equivalents [PicH][OTf] and 80 equivalents [PicMe][OTf] in DME monitoring the decay of a signal at 700 nm. An exponential fit of the data reveals an observed rate constant of 0.94 ± 0.01 × 10^6 s^{-1}.

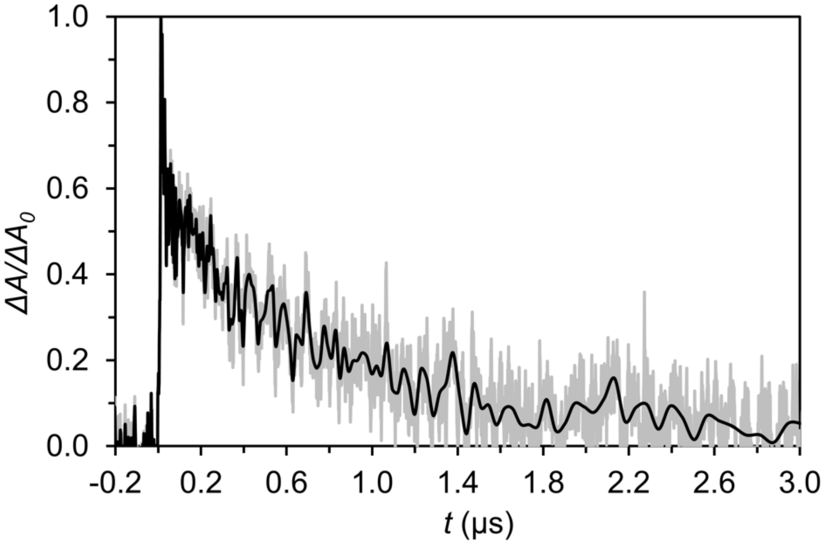

Fig. S51. Time-resolved transient absorption measurement of sample with **{Fc–N–an}**, 140 equivalents [PicH][OTf] and 60 equivalents [PicMe][OTf] in DME monitoring the decay of a signal at 700 nm. An exponential fit of the data reveals an observed rate constant of 1.10 ± 0.01 × 10^6 s^{-1}.

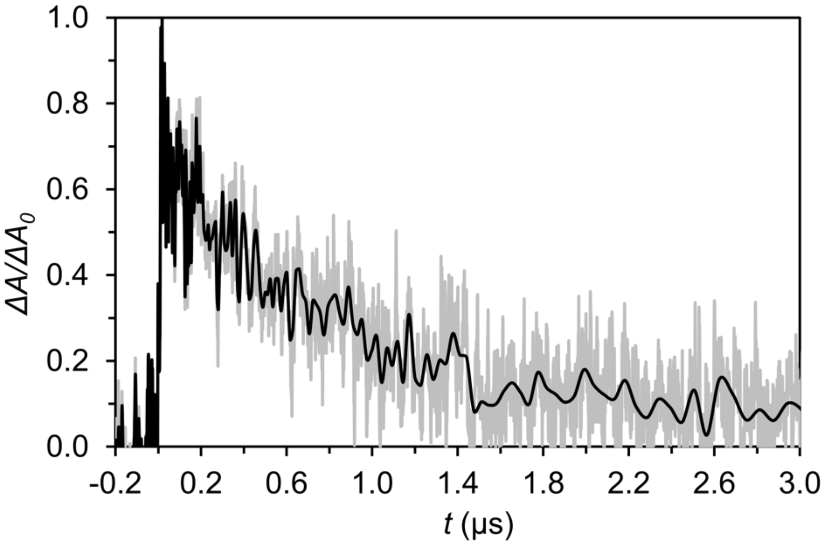

Fig. S52. Time-resolved transient absorption measurement of sample with **{Fc–N–an}**, 160 equivalents [PicH][OTf] and 40 equivalents [PicMe][OTf] in DME monitoring the decay of a signal at 700 nm. An exponential fit of the data reveals an observed rate constant of 1.11 ± 0.01 × 10^6 s^{-1}.

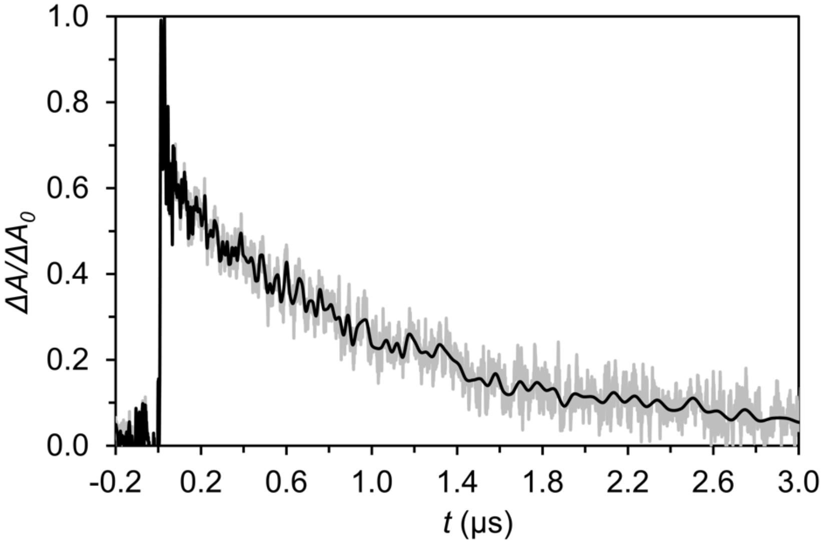

Fig. S53. Time-resolved transient absorption measurement of sample with **{Fc–N–an}**, 180 equivalents [PicH][OTf] and 20 equivalents [PicMe][OTf] in DME monitoring the decay of a signal at 700 nm. An exponential fit of the data reveals an observed rate constant of 1.15 ± 0.01 × 10^6 s^{-1}.

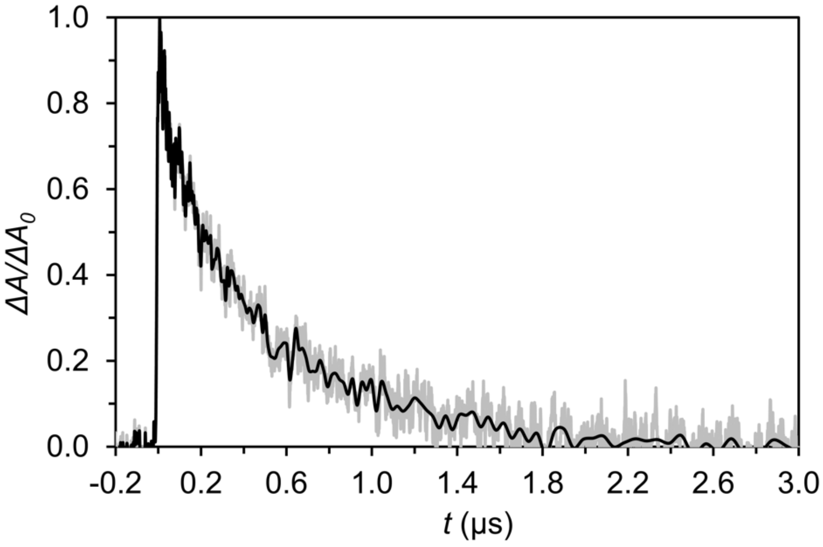

Fig. S54. Time-resolved transient absorption measurement of sample with **{Fc–N–an}**, 120 equivalents [PicH][OTf] and 80 equivalents [PicMe][OTf] with 12.5 mM acetophenone in DME monitoring the decay of a signal at 700 nm. An exponential fit of the data reveals an observed rate constant of $1.67 \pm 0.01 \times 10^6$ s^{-1}.

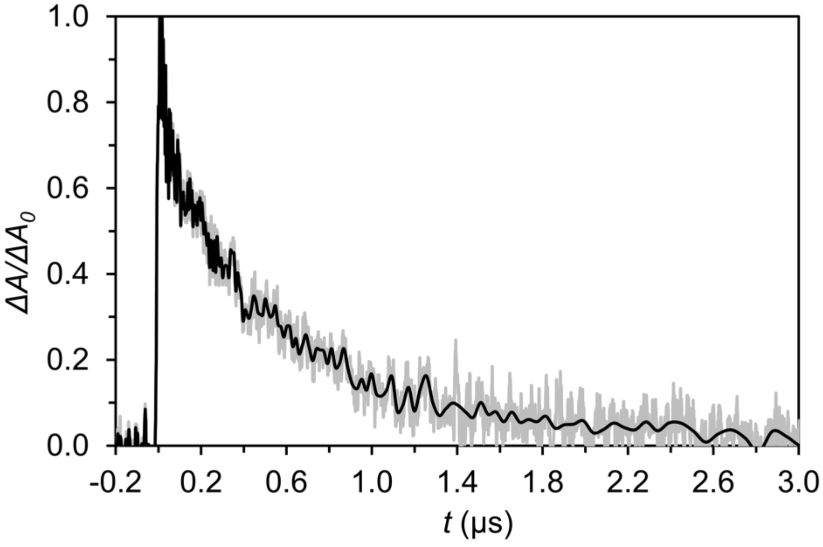

Fig. S55. Time-resolved transient absorption measurement of sample with **{Fc–N–an}**, 140 equivalents [PicH][OTf] and 60 equivalents [PicMe][OTf] with 12.5 mM acetophenone in DME monitoring the decay of a signal at 700 nm. An exponential fit of the data reveals an observed rate constant of $1.99 \pm 0.01 \times 10^6$ s^{-1}.

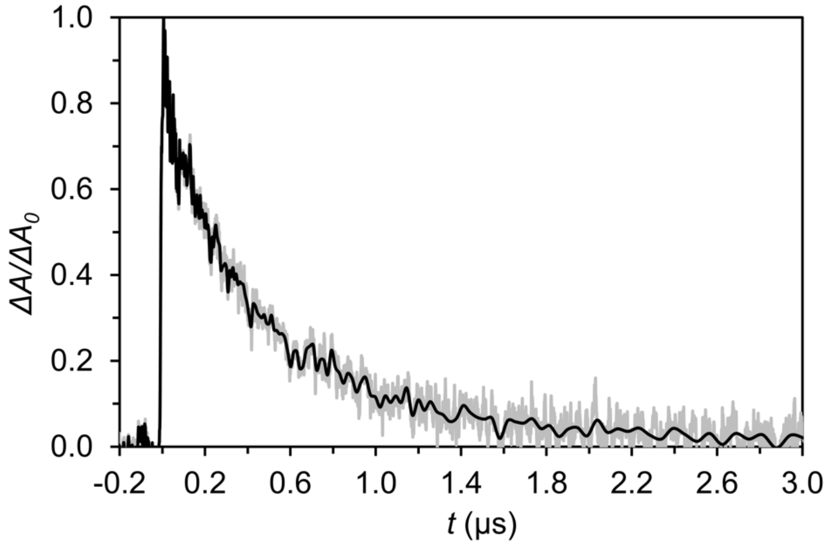

Fig. S56. Time-resolved transient absorption measurement of sample with **{Fc–N–an}**, 160 equivalents [PicH][OTf] and 40 equivalents [PicMe][OTf] with 12.5 mM acetophenone in DME monitoring the decay of a signal at 700 nm. An exponential fit of the data reveals an observed rate constant of $1.74 \pm 0.01 \times 10^6$ s^{-1}.

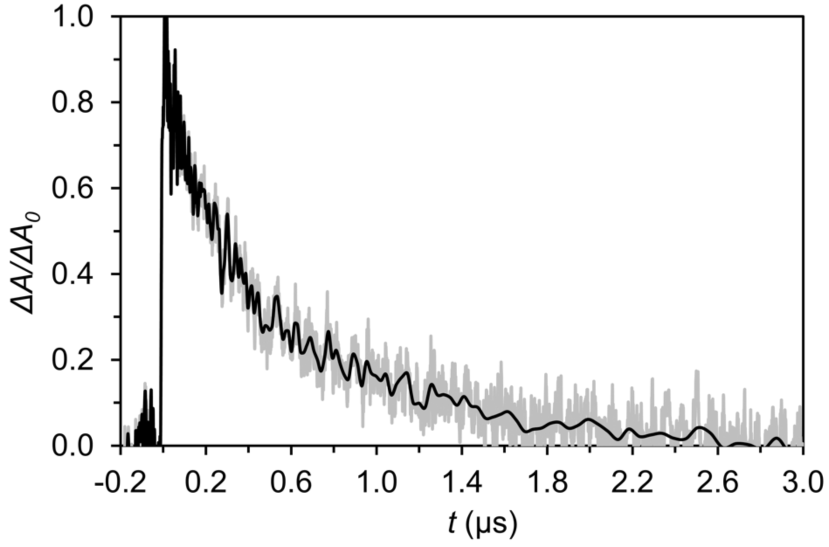

Fig. S57. Time-resolved transient absorption measurement of sample with **{Fc–N–an}**, 180 equivalents [PicH][OTf] and 20 equivalents [PicMe][OTf] with 12.5 mM acetophenone in DME monitoring the decay of a signal at 700 nm. An exponential fit of the data reveals an observed rate constant of $1.91 \pm 0.01 \times 10^6$ s^{-1}.

IV. Transient absorption spectra

Due to strong fluorescence from the sample overloading the detector, a filter (λ < 650 nm) was used to isolate the region of interest for collecting the transient absorption spectrum as overlapping fluorescence made these shorter-wavelength features difficult to observe. The sample was excited with a 355 nm laser pulse, and spectra were collected after various time delays from 50 to 1000 ns. These spectra show an exponential decay in intensity at 700 nm which is consistent with the data obtained in the time-resolved transient absorption experiments and with the spectroelectrochemistry data (see section S10).

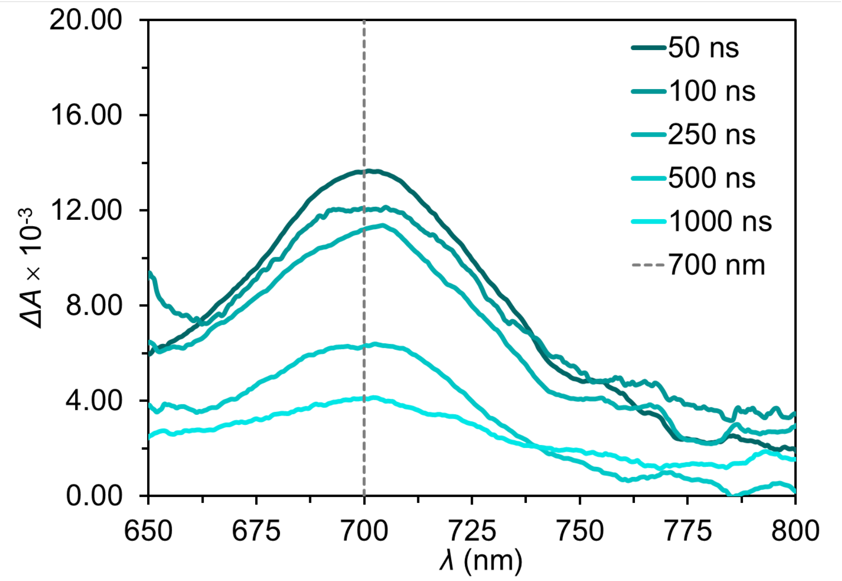

Fig. S58. Transient absorption spectra of **{Fc^{+}–NH^{+}–an$^{\bullet-}$}** in the presence of excess acid following time delays from 50 to 1000 ns.

V. Transient absorption at 420 nm

The triplet excited state, **{Fc–NH$^+$–^{3}an*},** is a potential PCET donor, albeit with a BDFE$_{N-H}$ that is about 16 kcal mol^{-1} greater than that of **{Fc$^+$–NH$^+$–an$^{•-}$}**. However, following excitation of **{Fc–NH$^+$–an},** decay of the 420-nm transient absorption characteristic of **^{3}an*** is substantially slower (τ = 2.1 μs) than that of the 700-nm transient absorption (τ = 0.9 μs) attributed to **{Fc$^+$–NH$^+$–an$^{•-}$}**. Moreover, in contrast to the 700-nm kinetics, the 420-nm kinetics are unchanged upon addition of acetophenone. The transient absorption data indicate that both **{Fc–NH$^+$–^{3}an*}** and **{Fc$^+$–NH$^+$–an$^{•-}$}** are formed upon 355 nm excitation, but the PCET reactivity with acetophenone occurs solely from **{Fc$^+$–NH$^+$–an$^{•-}$}.** For reference spectra of **^{3}an***, see ref. [16].

The 420-nm transient absorption signal of **{Fc–NH$^+$–^{3}an*}** is complicated by overlapping **{Fc–NH$^+$–^{1}an*}** fluorescence. The detection electronics do not recover from the strong fluorescence signal until ~0.1 μs after excitation so it is not possible to detect earlier events. For decay plots where the signal does not decay to zero, we postulate that **{Fc$^+$–NH$^+$–an$^{•-}$}** can reduce both [PicH][OTf] and acetophenone via PCET, and the oxidized **{Fc$^+$–NH$^+$–an}** has a positive absorbance at 420 nm. See section S10 for UV-vis spectra of **{Fc$^+$–N–an}**.

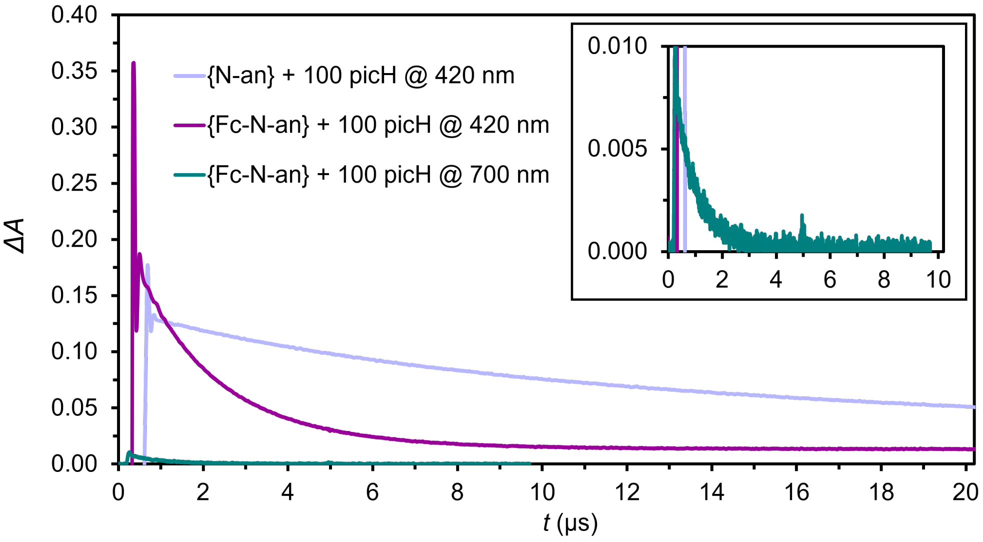

Fig. S59. Time-resolved transient absorption measurement of sample with **{Fc–N–an}** and **{N–an}** with 100 equivalents [PicH][OTf], in DME following the decay of a signal at 420 nm vs. 700 nm for **{Fc–N–an}**. A double-exponential fit of the **{N–an}** data reveals an observed rate constant of and 8.61 ± 0.01 × 10^4 s^{-1} for the first decay, and 1.595 ± 0.002 × 10^4 s^{-1} for the second rate constant (light purple trace).

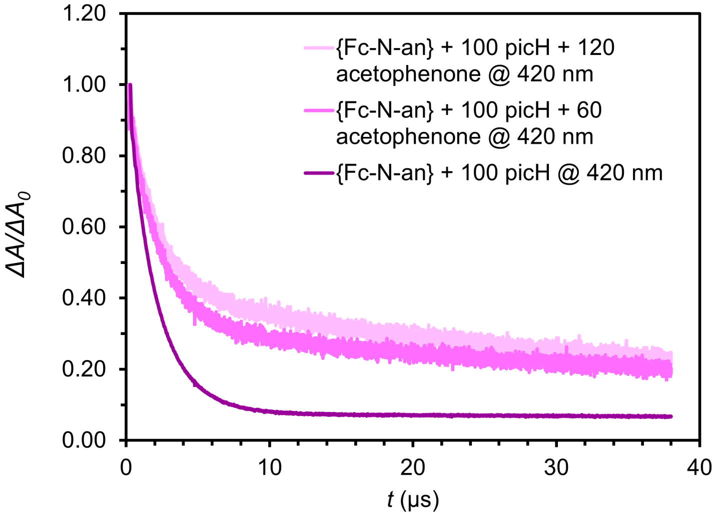

Fig. S60. Time-resolved transient absorption measurement of sample with **{Fc–N–an}** with 100 equivalents [PicH][OTf], in DME following the decay of a signal at 420 nm in the presence of varying concentrations of acetophenone. An exponential fit of the data reveals an observed rate constant of $5.03 \pm 0.02 \times 10^5$ s^{-1} for 9.4 mM acetophenone, and $5.21 \pm 0.02 \times 10^5$ s^{-1} for 120 equivalents of acetophenone. The traces for the acetophenone-containing measurements were fit to a bi-exponential and the first decay is presumed to be that of **{Fc–NH$^+$–^{3}an*}**.

V. Exponential fits

The data were fit to single and double exponential functions using a program in MATLAB to obtain the kinetic information from the time-resolved transient spectroscopy. Data were fit to the single exponential function $y = A(1)e^{(-k(1)t)} + A(2)$, or the double exponential function $y = A(1)e^{(-k(1)t)} + A(2)e^{(-k(2)t)} + A(3)$.

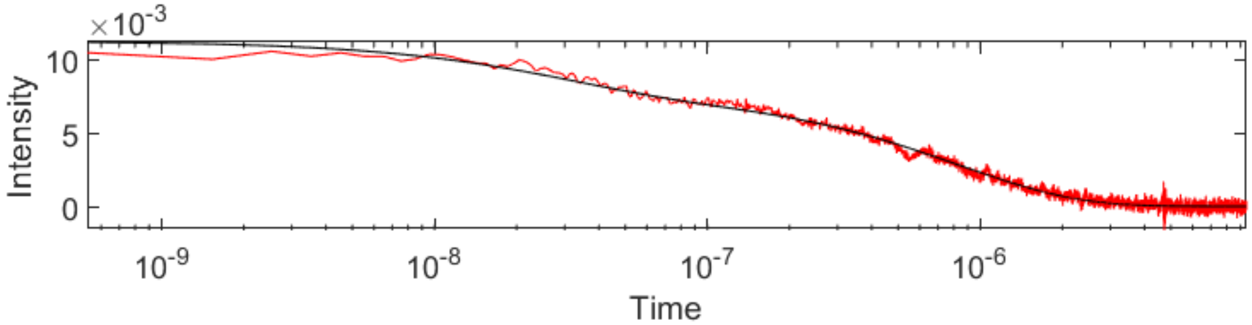

Rate Constant			Uncertainty (confidence limit)		
k(1) =	3.4866e+07	+/-	9.30e+05 (68%)	1.86e+06 (95%)	2.79e+06 (99%)
k(2) =	1.1349e+06	+/-	2.78e+03 (68%)	5.56e+03 (95%)	8.34e+03 (99%)

Coefficients:

A(1) =	3.5845e-03	+/-	6.77e-05 (68%)	1.35e-04 (95%)	2.03e-04 (99%)
A(2) =	7.6851e-03	+/-	1.26e-05 (68%)	2.51e-05 (95%)	3.77e-05 (99%)

Fig. S61. Exponential fit and associated errors for the decay of the transient signal generated at 700 nm from excitation of **{Fc–N–an}** with 100 equivalents [PicH][OTf].

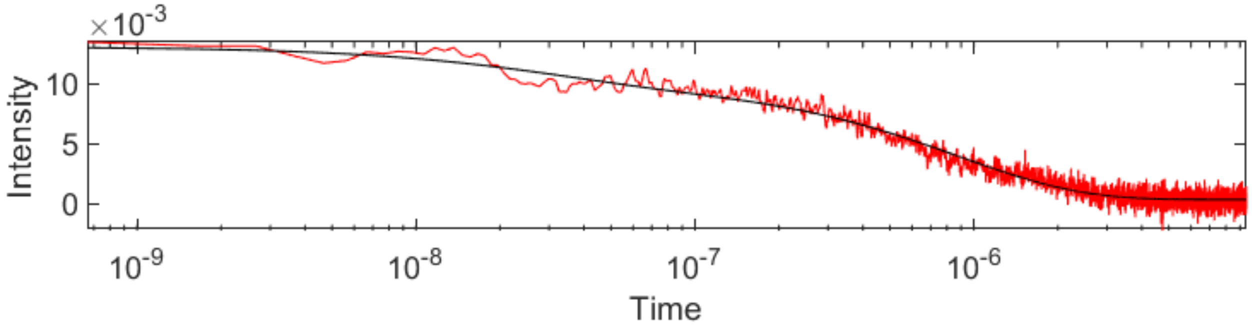

Rate Constant			Uncertainty (confidence limit)		
k(1) =	3.4820e+07	+/-	2.39e+06 (68%)	4.78e+06 (95%)	7.17e+06 (99%)
k(2) =	1.1727e+06	+/-	4.27e+03 (68%)	8.55e+03 (95%)	1.28e+04 (99%)

Coefficients:

A(1) =	2.9826e-03	+/-	1.46e-04 (68%)	2.91e-04 (95%)	4.37e-04 (99%)
A(2) =	9.7296e-03	+/-	2.60e-05 (68%)	5.21e-05 (95%)	7.81e-05 (99%)

Fig. S62. Exponential fit and associated errors for the decay of the transient signal generated at 700 nm from excitation of **{Fc–N–an}** with 100 equivalents [PicH][OTf] (replicate).

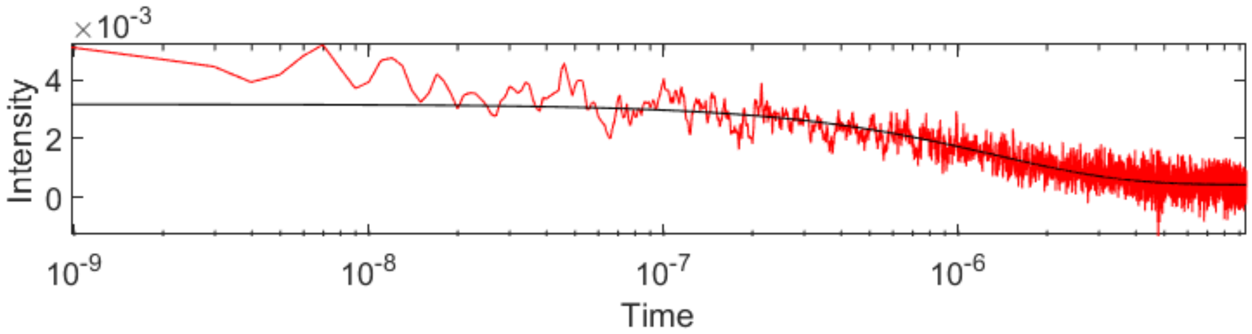

Rate Constant Uncertainty (confidence limit)

k(1) =	7.4776e+05	+/-	5.31e+03 (68%)	1.06e+04 (95%)	1.59e+04 (99%)

Coefficients:

A(1) =	2.7379e-03	+/-	1.38e-05 (68%)	2.75e-05 (95%)	4.13e-05 (99%)
A(2) =	4.2182e-04	+/-	3.65e-06 (68%)	7.30e-06 (95%)	1.10e-05 (99%)

Figure S63. Exponential fit and associated errors for the decay of the transient signal generated at 700 nm from excitation of **{Fc–NH$^+$–an}** with 15 mM [TBA][OTf].

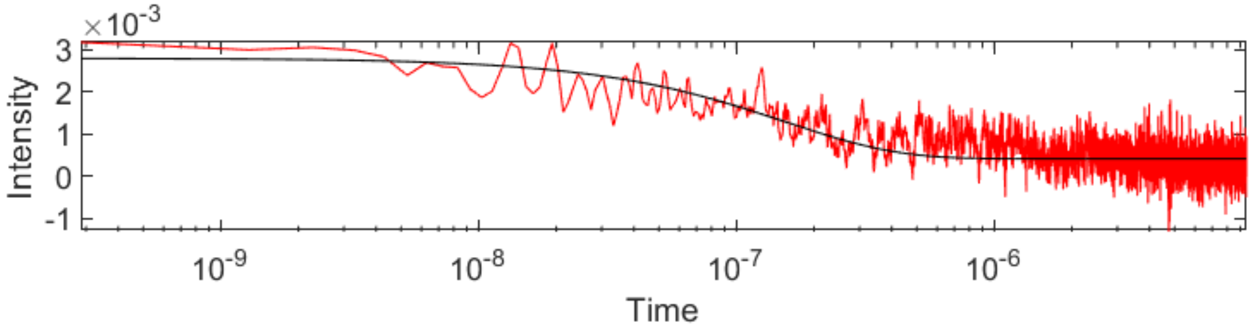

Rate Constant Uncertainty (confidence limit)

k(1) =	6.4664e+06	+/-	1.62e+05 (68%)	3.24e+05 (95%)	4.86e+05 (99%)

Coefficients:

A(1) =	2.3821e-03	+/-	4.21e-05 (68%)	8.43e-05 (95%)	1.26e-04 (99%)
A(2) =	4.1843e-04	+/-	3.81e-06 (68%)	7.62e-06 (95%)	1.14e-05 (99%)

Figure S64. Exponential fit and associated errors for the decay of the transient signal generated at 700 nm from excitation of **{Fc–NH$^+$–an}** with 15 mM $HNTs_2$.

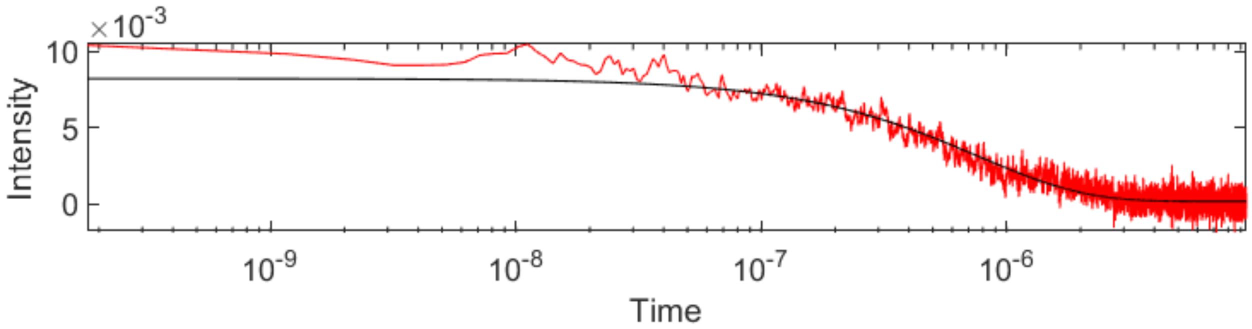

Rate Constant Uncertainty (confidence limit)

k(1) =	1.2939e+06	+/-	6.55e+03 (68%)	1.31e+04 (95%)	1.96e+04 (99%)

Coefficients:

A(1) =	8.0910e-03	+/-	2.87e-05 (68%)	5.73e-05 (95%)	8.60e-05 (99%)
A(2) =	1.4741e-04	+/-	5.76e-06 (68%)	1.15e-05 (95%)	1.73e-05 (99%)

Fig. S65. Exponential fit and associated errors for the decay of the transient signal generated at 700 nm from excitation of **{Fc–N–an}** with 100 equivalents [PicH][OTf] and 20 equivalents acetophenone (3.2 mM).

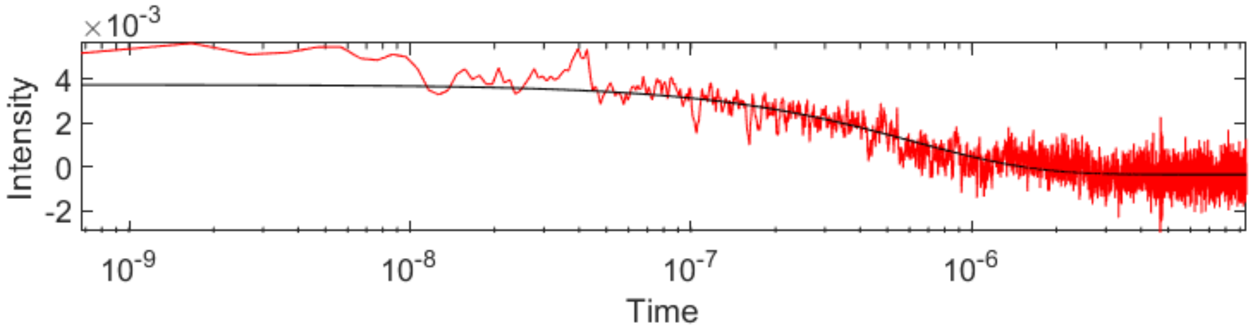

Rate Constant Uncertainty (confidence limit)

k(1) =	1.5817e+06	+/-	1.75e+04 (68%)	3.51e+04 (95%)	5.26e+04 (99%)

Coefficients:

A(1) =	4.0687e-03	+/-	3.13e-05 (68%)	6.25e-05 (95%)	9.38e-05 (99%)
A(2) =	-3.4086e-04	+/-	5.64e-06 (68%)	1.13e-05 (95%)	1.69e-05 (99%)

Fig. S66. Exponential fit and associated errors for the decay of the transient signal generated at 700 nm from excitation of **{Fc–N–an}** with 100 equivalents [PicH][OTf] and 40 equivalents acetophenone (6.4 mM).

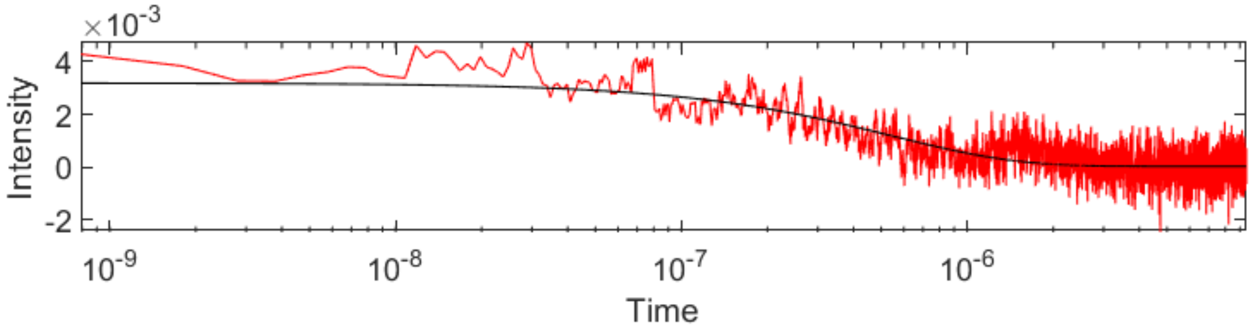

Rate Constant		Uncertainty (confidence limit)		
k(1) = 1.8860e+06	+/-	2.77e+04 (68%)	5.55e+04 (95%)	8.32e+04 (99%)

Coefficients:

A(1) = 3.1527e-03	+/-	3.36e-05 (68%)	6.71e-05 (95%)	1.01e-04 (99%)
A(2) = 3.0558e-05	+/-	5.68e-06 (68%)	1.14e-05 (95%)	1.70e-05 (99%)

Fig. S67. Exponential fit and associated errors for the decay of the transient signal generated at 700 nm from excitation of **{Fc–N–an}** with 100 equivalents [PicH][OTf] and 80 equivalents acetophenone (12.8 mM).

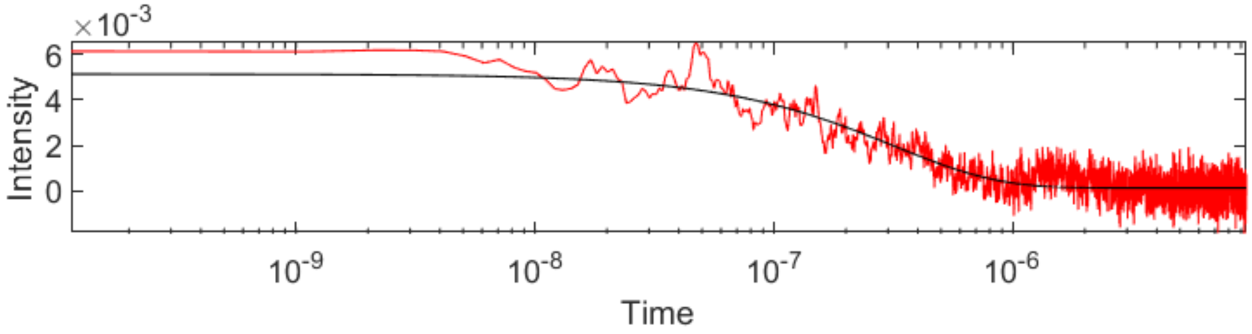

Rate Constant		Uncertainty (confidence limit)		
k(1) = 2.8947e+06	+/-	3.83e+04 (68%)	7.66e+04 (95%)	1.15e+05 (99%)

Coefficients:

A(1) = 5.0030e-03	+/-	4.31e-05 (68%)	8.62e-05 (95%)	1.29e-04 (99%)
A(2) = 1.3247e-04	+/-	5.59e-06 (68%)	1.12e-05 (95%)	1.68e-05 (99%)

Fig. S68. Exponential fit and associated errors for the decay of the transient signal generated at 700 nm from excitation of **{Fc–N–an}** with 100 equivalents [PicH][OTf] and 120 equivalents acetophenone (19.2 mM).

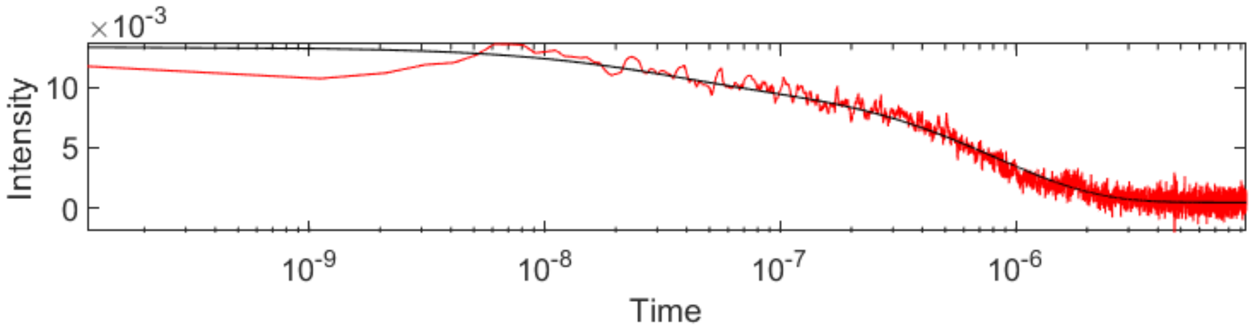

Rate Constant Uncertainty (confidence limit)

k(1) =	3.4749e+07	+/-	2.52e+06 (68%)	5.03e+06 (95%)	7.55e+06 (99%)
k(2) =	1.2116e+06	+/-	4.64e+03 (68%)	9.28e+03 (95%)	1.39e+04 (99%)

Coefficients:

A(1) =	2.8402e-03	+/-	1.47e-04 (68%)	2.95e-04 (95%)	4.42e-04 (99%)
A(2) =	1.0021e-02	+/-	2.72e-05 (68%)	5.43e-05 (95%)	8.15e-05 (99%)
A(3) =	4.7692e-04	+/-	5.66e-06 (68%)	1.13e-05 (95%)	1.70e-05 (99%)

Fig. S69. Exponential fit and associated errors for the decay of the transient signal generated at 700 nm from excitation of **{Fc–N–an}** with 100 equivalents [PicD][OTf].

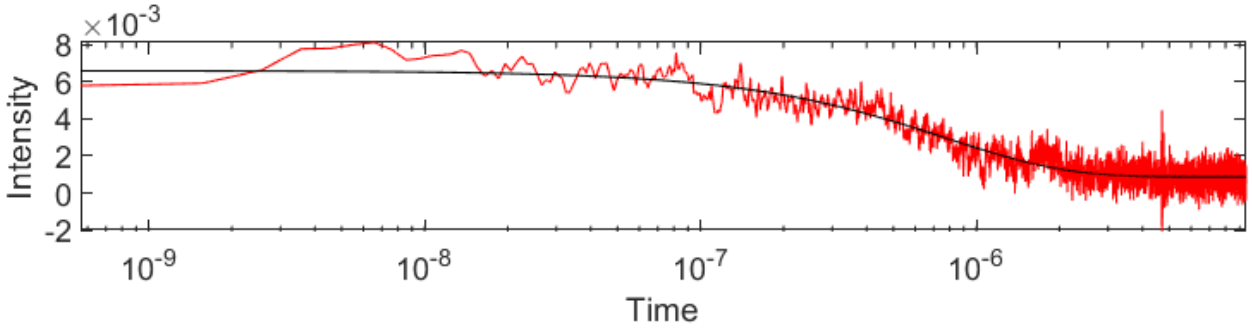

Rate Constant Uncertainty (confidence limit)

k(1) =	1.2987e+06	+/-	9.21e+03 (68%)	1.84e+04 (95%)	2.76e+04 (99%)

Coefficients:

A(1) =	5.7052e-03	+/-	2.86e-05 (68%)	5.72e-05 (95%)	8.58e-05 (99%)
A(2) =	8.7556e-04	+/-	5.76e-06 (68%)	1.15e-05 (95%)	1.73e-05 (99%)

Fig. S70. Exponential fit and associated errors for the decay of the transient signal generated at 700 nm from excitation of **{Fc–N–an}** with 100 equivalents [PicD][OTf] and 20 equivalents acetophenone (3.2 mM).

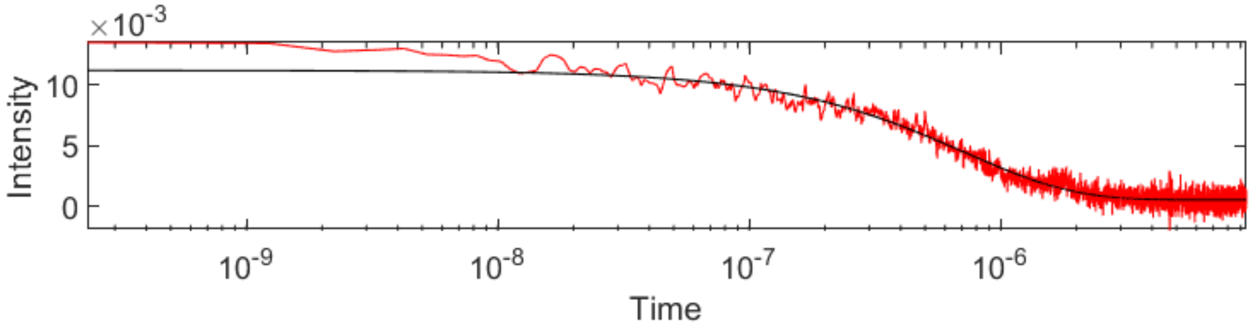

Rate Constant Uncertainty (confidence limit)
k(1) = 1.3943e+06 +/- 5.30e+03 (68%) 1.06e+04 (95%) 1.59e+04 (99%)

Coefficients:
A(1) = 1.0691e-02 +/- 2.87e-05 (68%) 5.75e-05 (95%) 8.62e-05 (99%)
A(2) = 5.3759e-04 +/- 5.59e-06 (68%) 1.12e-05 (95%) 1.68e-05 (99%)

Fig. S71. Exponential fit and associated errors for the decay of the transient signal generated at 700 nm from excitation of **{Fc–N–an}** with 100 equivalents [PicD][OTf] and 40 equivalents acetophenone (6.4 mM).

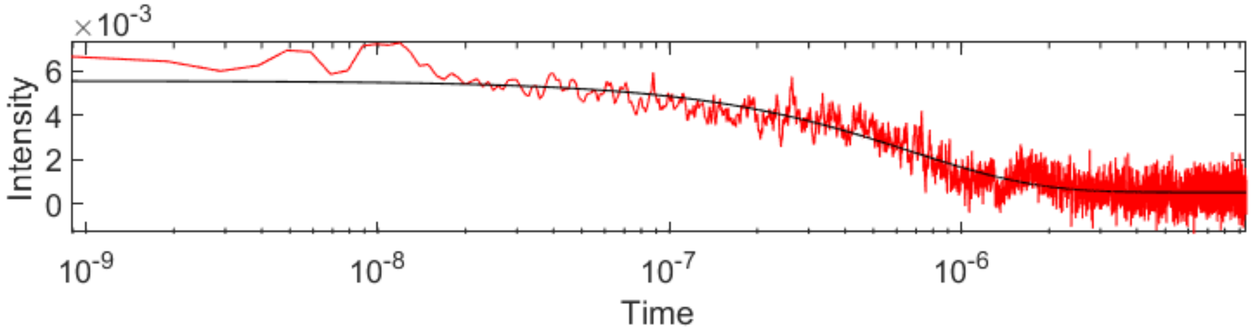

Rate Constant Uncertainty (confidence limit)
k(1) = 1.4890e+06 +/- 1.24e+04 (68%) 2.49e+04 (95%) 3.73e+04 (99%)

Coefficients:
A(1) = 5.0450e-03 +/- 2.98e-05 (68%) 5.97e-05 (95%) 8.95e-05 (99%)
A(2) = 5.1050e-04 +/- 5.61e-06 (68%) 1.12e-05 (95%) 1.68e-05 (99%)

Fig. S72. Exponential fit and associated errors for the decay of the transient signal generated at 700 nm from excitation of **{Fc–N–an}** with 100 equivalents [PicD][OTf] and 80 equivalents acetophenone (12.8 mM).

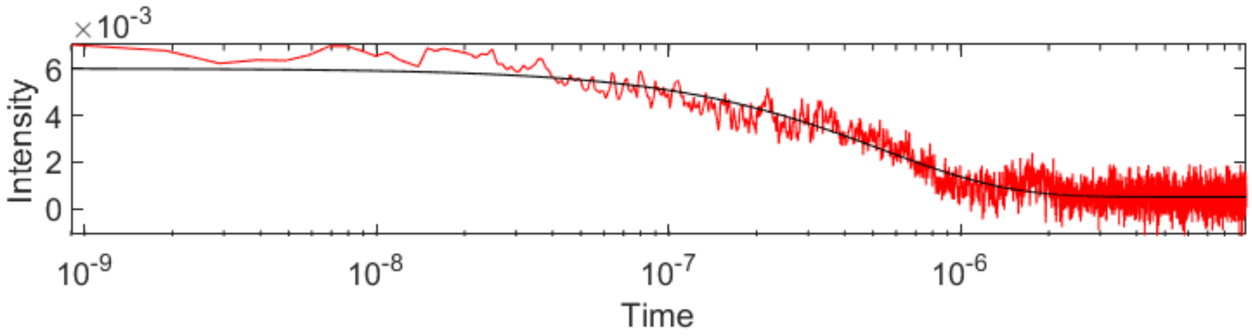

Rate Constant Uncertainty (confidence limit)
k(1) = 1.8563e+06 +/- 1.41e+04 (68%) 2.81e+04 (95%) 4.22e+04 (99%)

Coefficients:
A(1) = 5.5054e-03 +/- 2.95e-05 (68%) 5.90e-05 (95%) 8.85e-05 (99%)
A(2) = 5.0490e-04 +/- 4.97e-06 (68%) 9.93e-06 (95%) 1.49e-05 (99%)

Fig. S73. Exponential fit and associated errors for the decay of the transient signal generated at 700 nm from excitation of **{Fc–N–an}** with 100 equivalents [PicD][OTf] and 120 equivalents acetophenone (19.2 mM).

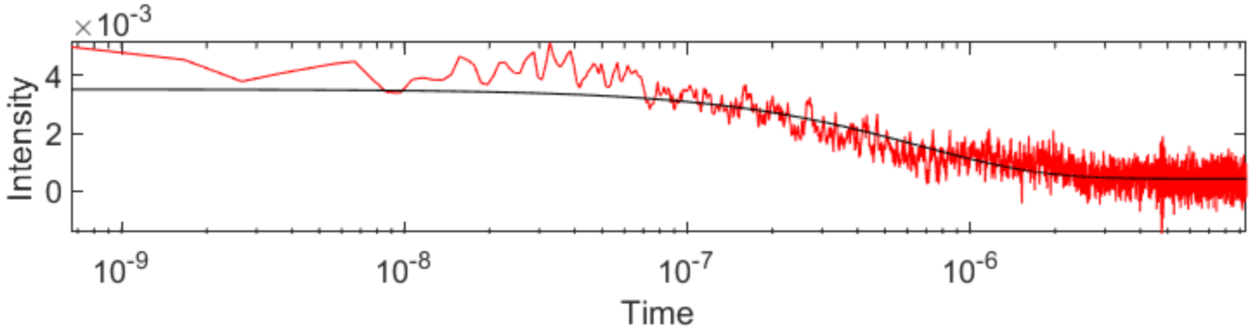

Rate Constant Uncertainty (confidence limit)
k(1) = 1.5057e+06 +/- 1.45e+04 (68%) 2.90e+04 (95%) 4.36e+04 (99%)

Coefficients:
A(1) = 3.1041e-03 +/- 2.12e-05 (68%) 4.23e-05 (95%) 6.35e-05 (99%)
A(2) = 4.2069e-04 +/- 3.96e-06 (68%) 7.92e-06 (95%) 1.19e-05 (99%)

Fig. S74. Exponential fit and associated errors for the decay of the transient signal generated at 700 nm from excitation of **{Fc–NH$^+$-an}**.

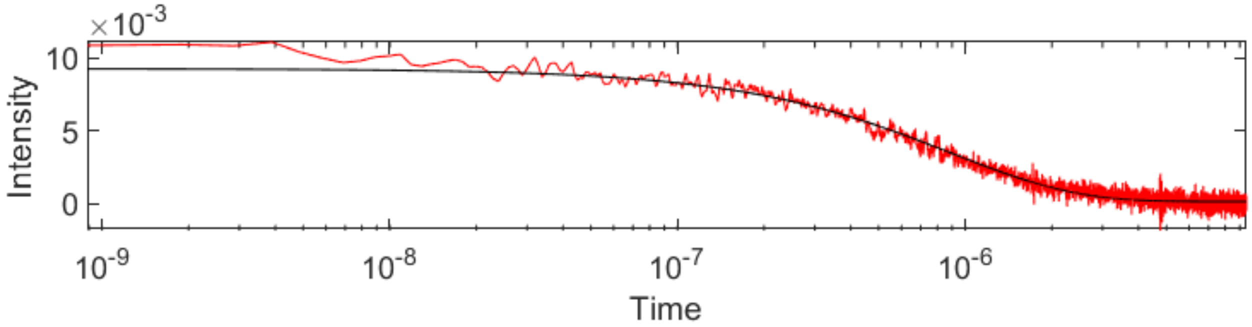

Rate Constant		Uncertainty (confidence limit)		
k(1) = 1.1277e+06	+/-	3.15e+03 (68%)	6.31e+03 (95%)	9.46e+03 (99%)

Coefficients:				
A(1) = 9.0687e-03	+/-	1.79e-05 (68%)	3.59e-05 (95%)	5.38e-05 (99%)
A(2) = 1.7464e-04	+/-	3.88e-06 (68%)	7.76e-06 (95%)	1.16e-05 (99%)

Fig. S75. Exponential fit and associated errors for the decay of the transient signal generated at 700 nm from excitation of **{Fc–NMe$^+$-an}** with 100 equivalents [PicH][OTf].

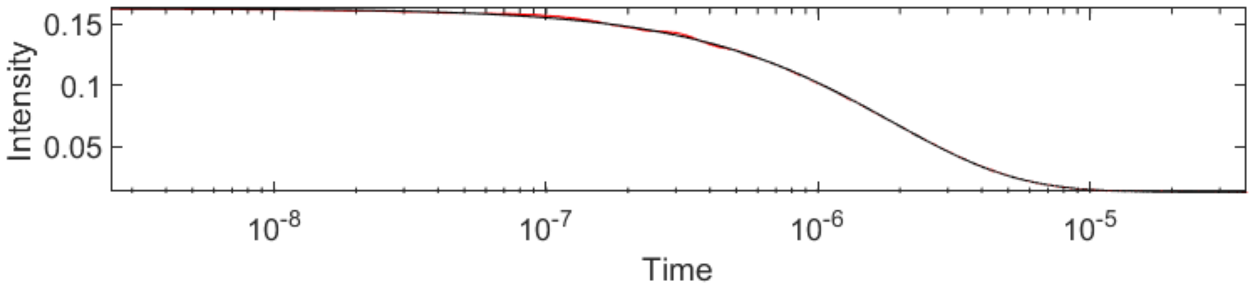

Rate Constant		Uncertainty (confidence limit)		
k(1) = 5.7622e+05	+/-	1.24e+02 (68%)	2.49e+02 (95%)	3.73e+02 (99%)
k(2) = 2.6223e+05	+/-	2.11e+02 (68%)	4.21e+02 (95%)	6.32e+02 (99%)

Coefficients:				
A(1) = 1.2673e-01	+/-	1.94e-05 (68%)	3.87e-05 (95%)	5.81e-05 (99%)
A(2) = 2.2997e-02	+/-	1.31e-05 (68%)	2.61e-05 (95%)	3.92e-05 (99%)
A(3) = 1.2937e-02	+/-	2.94e-06 (68%)	5.88e-06 (95%)	8.82e-06 (99%)

Fig. S76. Double-exponential fit and associated errors for the decay of the transient signal generated at 420 nm from excitation of **{Fc–N–an}** with 100 equivalents [PicH][OTf].

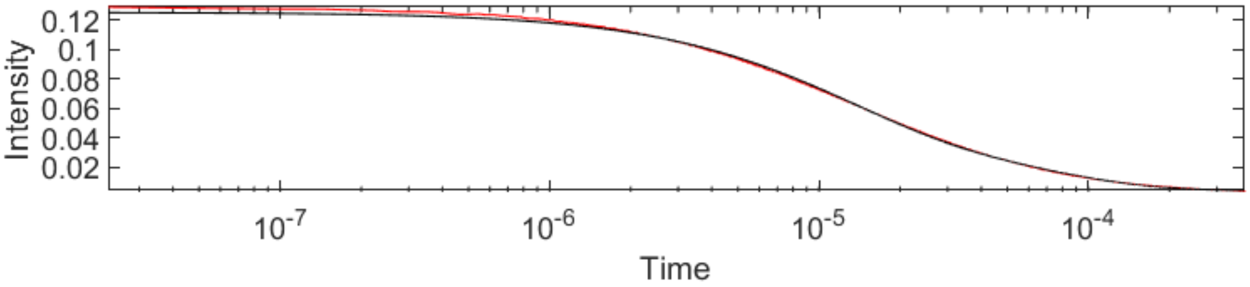

Rate Constant			Uncertainty (confidence limit)		
k(1) =	8.6196e+04	+/-	5.78e+01 (68%)	1.16e+02 (95%)	1.74e+02 (99%)
k(2) =	1.5946e+04	+/-	8.55e+00 (68%)	1.71e+01 (95%)	2.56e+01 (99%)

Coefficients:

A(1) =	7.8640e-02	+/-	3.73e-05 (68%)	7.47e-05 (95%)	1.12e-04 (99%)
A(2) =	4.2349e-02	+/-	1.61e-05 (68%)	3.21e-05 (95%)	4.82e-05 (99%)
A(3) =	4.3561e-03	+/-	4.61e-06 (68%)	9.23e-06 (95%)	1.38e-05 (99%)

Fig. S77. Double-exponential fit and associated errors for the decay of the transient signal generated at 420 nm from excitation of **{N–an}** with 100 equivalents [PicH][OTf].

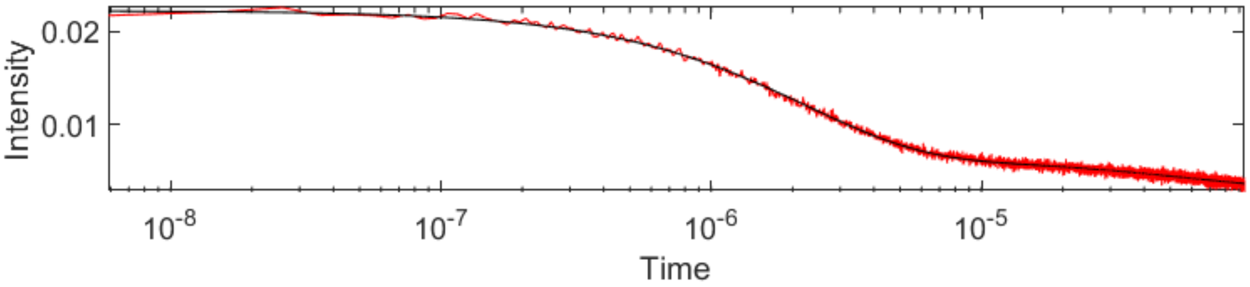

Rate Constant			Uncertainty (confidence limit)		
k(1) =	4.5181e+05	+/-	1.13e+03 (68%)	2.27e+03 (95%)	3.40e+03 (99%)
k(2) =	1.6337e+04	+/-	4.51e+01 (68%)	9.01e+01 (95%)	1.35e+02 (99%)

Coefficients:

A(1) =	1.5845e-02	+/-	2.81e-05 (68%)	5.62e-05 (95%)	8.43e-05 (99%)
A(2) =	3.5589e-03	+/-	5.47e-06 (68%)	1.09e-05 (95%)	1.64e-05 (99%)
A(3) =	2.8644e-03	+/-	3.05e-06 (68%)	6.10e-06 (95%)	9.15e-06 (99%)

Fig. S78. Double-exponential fit and associated errors for the decay of the transient signal generated at 420 nm from excitation of **{Fc–N–an}** with 100 equivalents [PicH][OTf] and 20 equivalents acetophenone (3.2 mM).

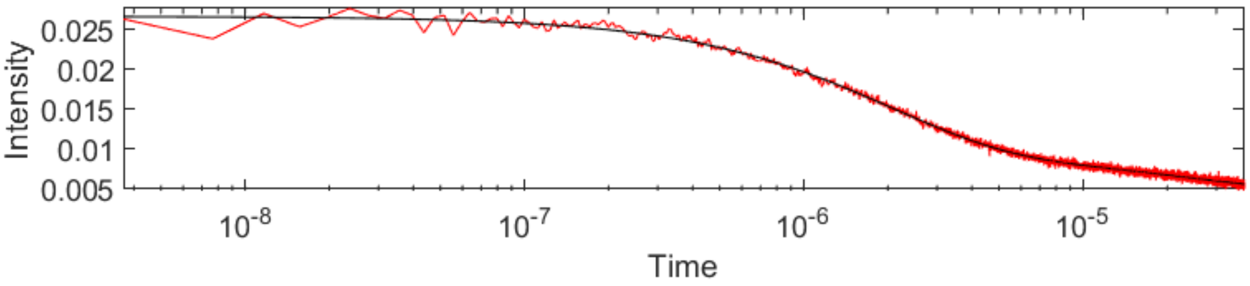

Rate Constant			Uncertainty (confidence limit)		
k(1) =	5.0306e+05	+/-	7.65e+02 (68%)	1.53e+03 (95%)	2.29e+03 (99%)
k(2) =	4.0775e+04	+/-	7.87e+01 (68%)	1.57e+02 (95%)	2.36e+02 (99%)

Coefficients:

A(1) =	1.7151e-02	+/-	1.84e-05 (68%)	3.69e-05 (95%)	5.53e-05 (99%)
A(2) =	4.9804e-03	+/-	5.37e-06 (68%)	1.07e-05 (95%)	1.61e-05 (99%)
A(3) =	4.5427e-03	+/-	2.99e-06 (68%)	5.97e-06 (95%)	8.96e-06 (99%)

Fig. S79. Double-exponential fit and associated errors for the decay of the transient signal generated at 420 nm from excitation of **{Fc–N–an}** with 100 equivalents [PicH][OTf] and 60 equivalents acetophenone (7.2 mM).

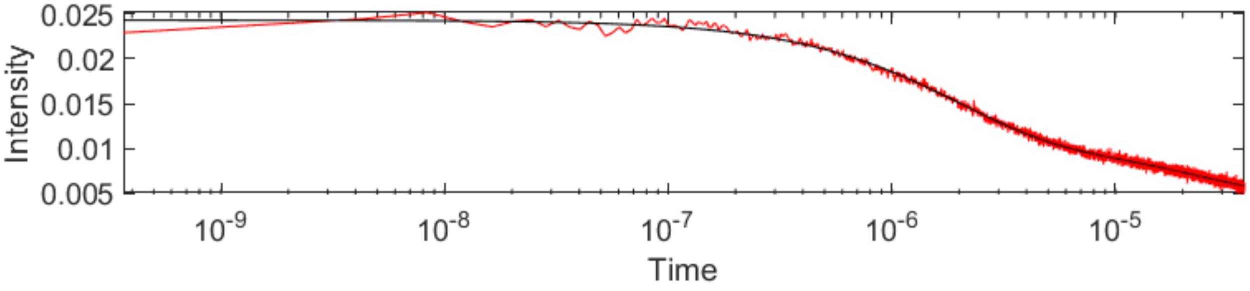

Rate Constant			Uncertainty (confidence limit)		
k(1) =	5.2071e+05	+/-	1.12e+03 (68%)	2.25e+03 (95%)	3.37e+03 (99%)
k(2) =	3.8669e+04	+/-	6.34e+01 (68%)	1.27e+02 (95%)	1.90e+02 (99%)

Coefficients:

A(1) =	1.4026e-02	+/-	2.14e-05 (68%)	4.28e-05 (95%)	6.42e-05 (99%)
A(2) =	6.7128e-03	+/-	5.99e-06 (68%)	1.20e-05 (95%)	1.80e-05 (99%)
A(3) =	4.2952e-03	+/-	3.40e-06 (68%)	6.81e-06 (95%)	1.02e-05 (99%)

Fig. S80. Double-exponential fit and associated errors for the decay of the transient signal generated at 420 nm from excitation of **{Fc–N–an}** with 100 equivalents [PicH][OTf] and 120 equivalents acetophenone (19.2 mM).

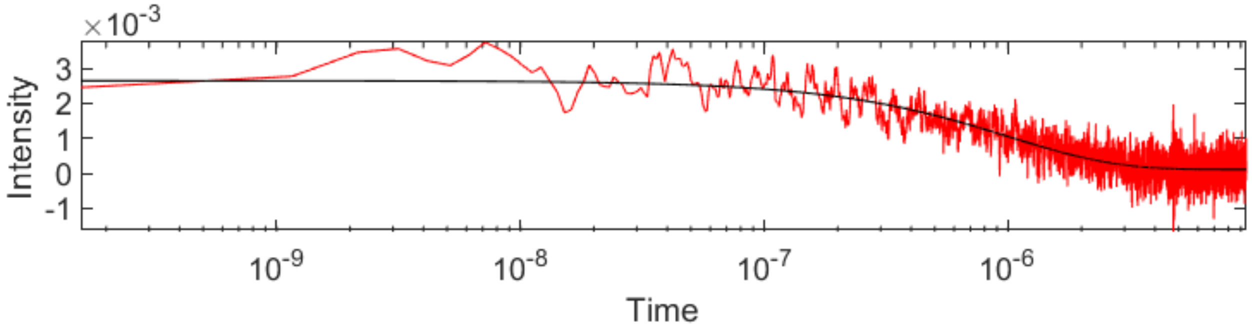

Rate Constant			Uncertainty (confidence limit)		
k(1) =	9.9095e+05	+/-	8.89e+03 (68%)	1.78e+04 (95%)	2.67e+04 (99%)

Coefficients:

A(1) =	2.5373e-03	+/-	1.61e-05 (68%)	3.22e-05 (95%)	4.83e-05 (99%)
A(2) =	1.1212e-04	+/-	3.71e-06 (68%)	7.42e-06 (95%)	1.11e-05 (99%)

Fig. S81. Exponential fit and associated errors for the decay of the transient signal generated at 700 nm from excitation of **{Fc–N–an}** with 31.7 mM [PicH][OTf].

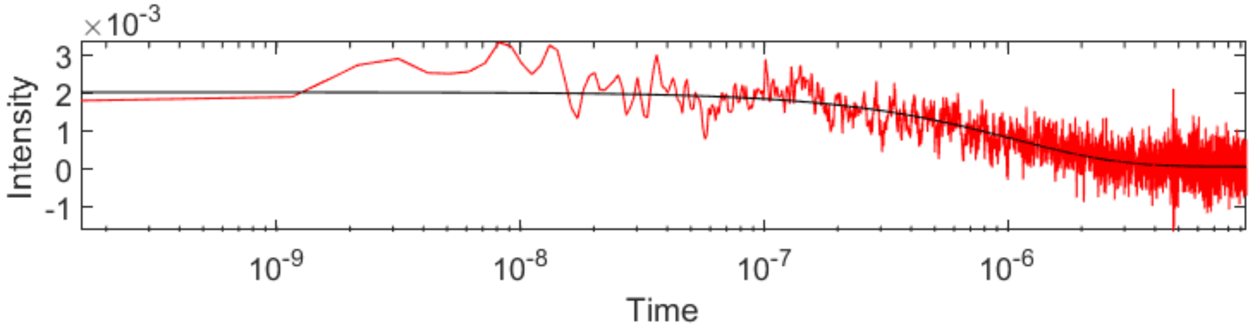

Rate Constant			Uncertainty (confidence limit)		
k(1) =	9.4377e+05	+/-	1.06e+04 (68%)	2.11e+04 (95%)	3.17e+04 (99%)

Coefficients:

A(1) =	1.9546e-03	+/-	1.55e-05 (68%)	3.10e-05 (95%)	4.64e-05 (99%)
A(2) =	7.4171e-05	+/-	3.66e-06 (68%)	7.32e-06 (95%)	1.10e-05 (99%)

Fig. S82. Exponential fit and associated errors for the decay of the transient signal generated at 700 nm from excitation of **{Fc–N–an}** with 28.6 mM [PicH][OTf] + 3.2 mM [PicMe][OTf].

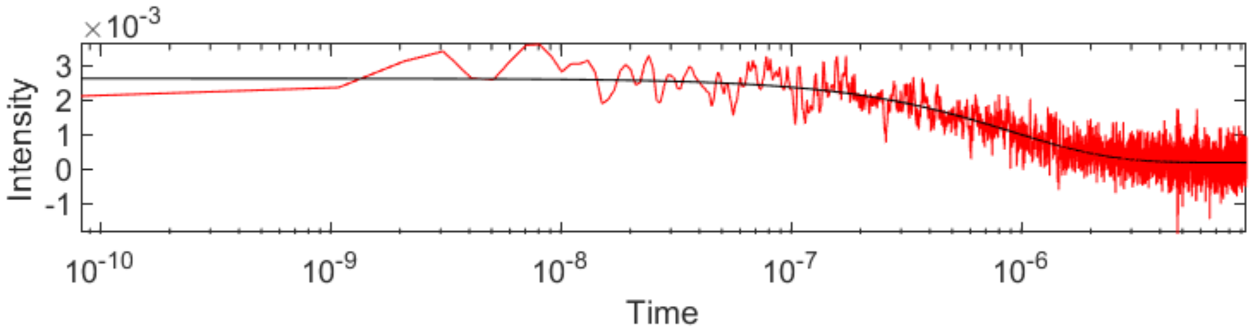

Rate Constant Uncertainty (confidence limit)

k(1) =	1.1026e+06	+/-	1.09e+04 (68%)	2.17e+04 (95%)	3.26e+04 (99%)

Coefficients:

A(1) =	2.4342e-03	+/-	1.69e-05 (68%)	3.39e-05 (95%)	5.08e-05 (99%)
A(2) =	1.9011e-04	+/-	3.71e-06 (68%)	7.42e-06 (95%)	1.11e-05 (99%)

Fig. S83. Exponential fit and associated errors for the decay of the transient signal generated at 700 nm from excitation of **{Fc–N–an}** with 25.4 mM [PicH][OTf] + 6.3 mM [PicMe][OTf].

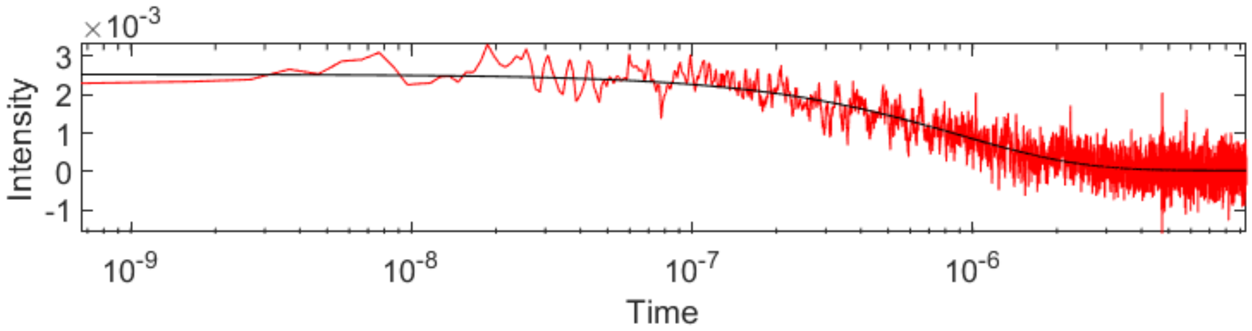

Rate Constant Uncertainty (confidence limit)

k(1) =	1.1089e+06	+/-	1.02e+04 (68%)	2.04e+04 (95%)	3.06e+04 (99%)

Coefficients:

A(1) =	2.4988e-03	+/-	1.62e-05 (68%)	3.25e-05 (95%)	4.87e-05 (99%)
A(2) =	3.1006e-05	+/-	3.55e-06 (68%)	7.09e-06 (95%)	1.06e-05 (99%)

Fig. S84. Exponential fit and associated errors for the decay of the transient signal generated at 700 nm from excitation of **{Fc–N–an}** with 22.2 mM [PicH][OTf] + 9.5 mM [PicMe][OTf].

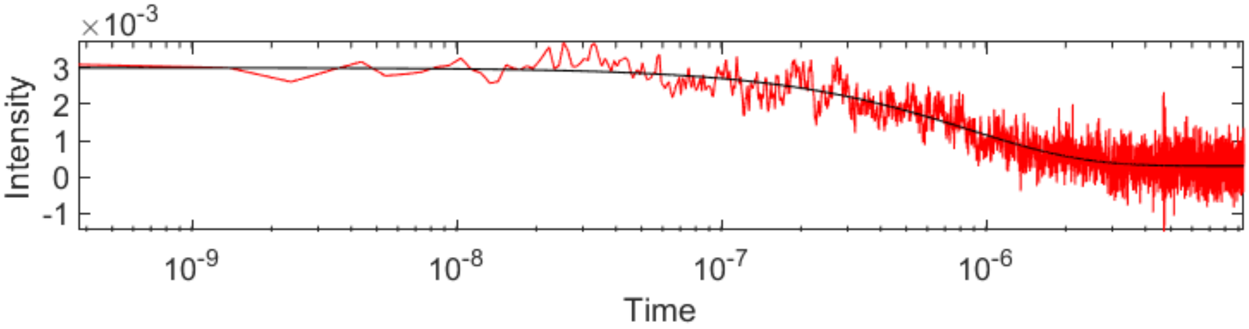

Rate Constant		Uncertainty (confidence limit)		
k(1) = 1.1521e+06	+/-	1.05e+04 (68%)	2.10e+04 (95%)	3.15e+04 (99%)

Coefficients:				
A(1) = 2.7067e-03	+/-	1.75e-05 (68%)	3.49e-05 (95%)	5.24e-05 (99%)
A(2) = 2.9915e-04	+/-	3.75e-06 (68%)	7.49e-06 (95%)	1.12e-05 (99%)

Fig. S85. Exponential fit and associated errors for the decay of the transient signal generated at 700 nm from excitation of **{Fc–N–an}** with 19.0 mM [PicH][OTf] + 12.7 mM [PicMe][OTf].

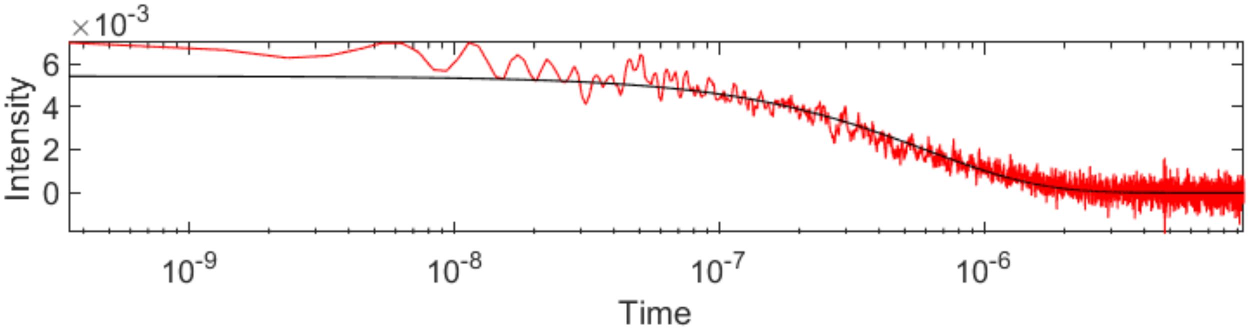

Rate Constant		Uncertainty (confidence limit)		
k(1) = 1.6682e+06	+/-	8.79e+03 (68%)	1.76e+04 (95%)	2.64e+04 (99%)

Coefficients:				
A(1) = 5.4475e-03	+/-	2.03e-05 (68%)	4.06e-05 (95%)	6.09e-05 (99%)
A(2) = -2.4536e-05	+/-	3.61e-06 (68%)	7.21e-06 (95%)	1.08e-05 (99%)

Fig. S86. Exponential fit and associated errors for the decay of the transient signal generated at 700 nm from excitation of **{Fc–N–an}** with 28.6 mM [PicH][OTf] + 3.2 mM [PicMe][OTf] and 12.7 mM acetophenone.

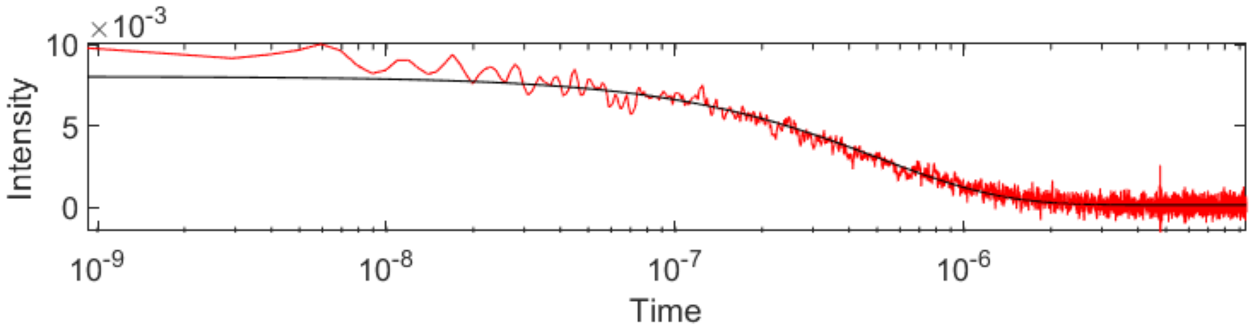

Rate Constant		Uncertainty (confidence limit)		
k(1) =	1.9886e+06 +/-	7.90e+03 (68%)	1.58e+04 (95%)	2.37e+04 (99%)

Coefficients:

A(1) =	7.8942e-03 +/-	2.22e-05 (68%)	4.44e-05 (95%)	6.66e-05 (99%)
A(2) =	1.3572e-04 +/-	3.61e-06 (68%)	7.22e-06 (95%)	1.08e-05 (99%)

Fig. S87. Exponential fit and associated errors for the decay of the transient signal generated at 700 nm from excitation of **{Fc–N–an}** with 25.4 mM [PicH][OTf] + 6.3 mM [PicMe][OTf] and 12.7 mM acetophenone.

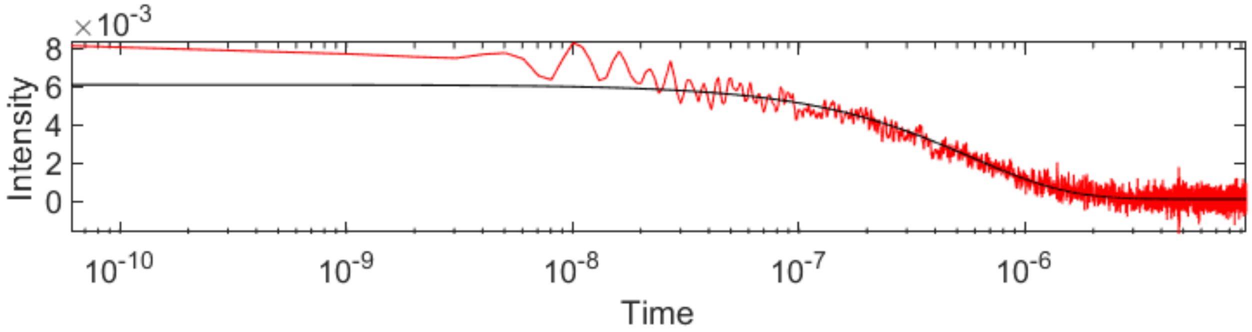

Rate Constant		Uncertainty (confidence limit)		
k(1) =	1.7397e+06 +/-	8.36e+03 (68%)	1.67e+04 (95%)	2.51e+04 (99%)

Coefficients:

A(1) =	5.9914e-03 +/-	2.03e-05 (68%)	4.07e-05 (95%)	6.10e-05 (99%)
A(2) =	1.1563e-04 +/-	3.54e-06 (68%)	7.08e-06 (95%)	1.06e-05 (99%)

Fig. S88. Exponential fit and associated errors for the decay of the transient signal generated at 700 nm from excitation of **{Fc–N–an}** with 22.2 mM [PicH][OTf] + 9.5 mM [PicMe][OTf] and 12.7 mM acetophenone.

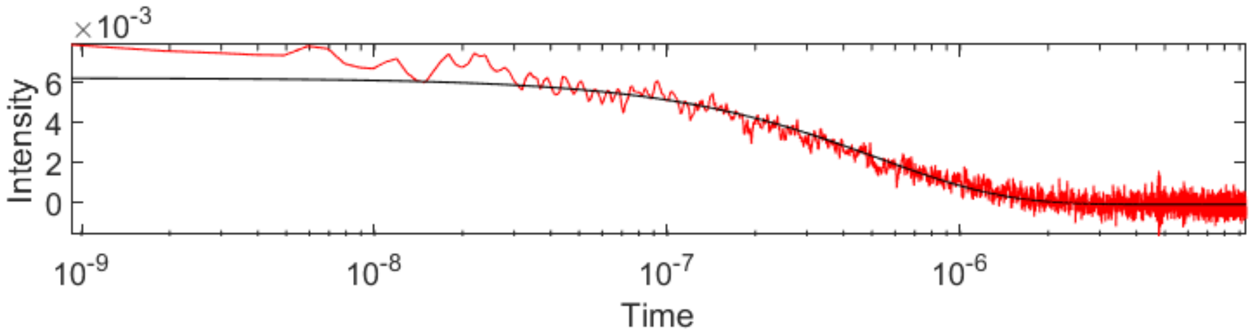

Rate Constant		Uncertainty (confidence limit)		
k(1) = 1.9086e+06	+/-	9.05e+03 (68%)	1.81e+04 (95%)	2.71e+04 (99%)

Coefficients:

A(1) = 6.2806e-03	+/-	2.11e-05 (68%)	4.21e-05 (95%)	6.32e-05 (99%)
A(2) = -6.8391e-05	+/-	3.50e-06 (68%)	7.00e-06 (95%)	1.05e-05 (99%)

Fig. S89. Exponential fit and associated errors for the decay of the transient signal generated at 700 nm from excitation of **{Fc–N–an}** with 19.0 mM [PicH][OTf] + 12.7 mM [PicMe][OTf] and 12.7 mM acetophenone.

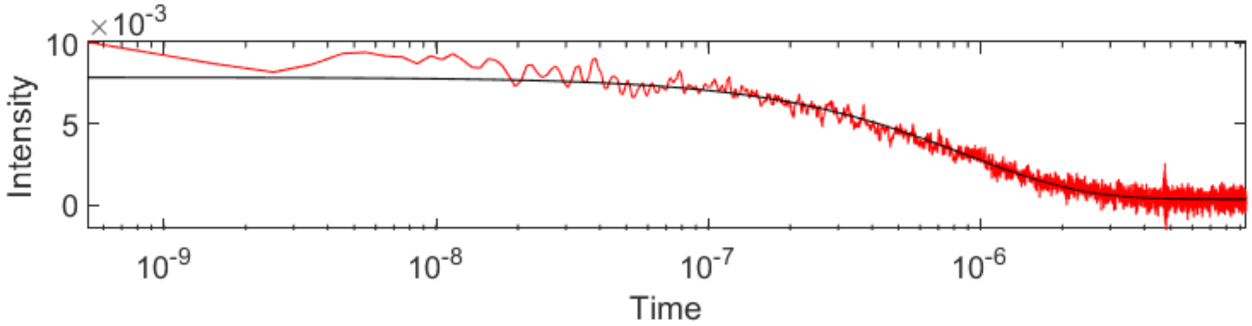

Rate Constant		Uncertainty (confidence limit)		
k(1) = 1.1366e+06	+/-	3.65e+03 (68%)	7.30e+03 (95%)	1.10e+04 (99%)

Coefficients:

A(1) = 7.5409e-03	+/-	1.71e-05 (68%)	3.43e-05 (95%)	5.14e-05 (99%)
A(2) = 3.4226e-04	+/-	3.69e-06 (68%)	7.38e-06 (95%)	1.11e-05 (99%)

Fig. S90. Exponential fit and associated errors for the decay of the transient signal generated at 700 nm from excitation of **{Fc–NH$^+$-an}** with 16 mM [PicMe][OTf].

S9. Reaction quantum yield determination

Relative quantum yield was determined through the potassium ferrioxalate chemical actinometry method.[17] $K_3Fe(C_2O_4)_3$ was prepared and used in solution by making a 10 mM Fe_2SO_4 and 60 mM $K_2C_2O_4$ stock solution in 4 % H_2SO_4 (aq). 3 mL of this solution was irradiated at 390 nm for 5 seconds in the same setup as employed for all stoichiometric reactions with **{Fc–N–an}**. Care was taken to minimize light exposure between irradiation cycles.

A 0.2 % by weight solution of 1,10-phenanthroline in water and a 0.6 M NaOAc buffer in 1 % H_2SO_4 (aq) were prepared separately. A 100 µL aliquot of the irradiated solution was placed into a 10 mL volumetric flask along with 200 µL of the phenanthroline solution and 50 µL of buffer, and the solution was diluted with water. The complexation of Fe^{2+} with 1,10-phenanthroline resulted in a bright red solution that had a characteristic absorption at 510 nm. For the control, these steps were repeated with a 100 µL aliquot of non-irradiated solution.

$$2Fe^{3+} + C_2O_4^{2-} \xrightarrow{hv} 2Fe^{2+} + 2CO_2$$

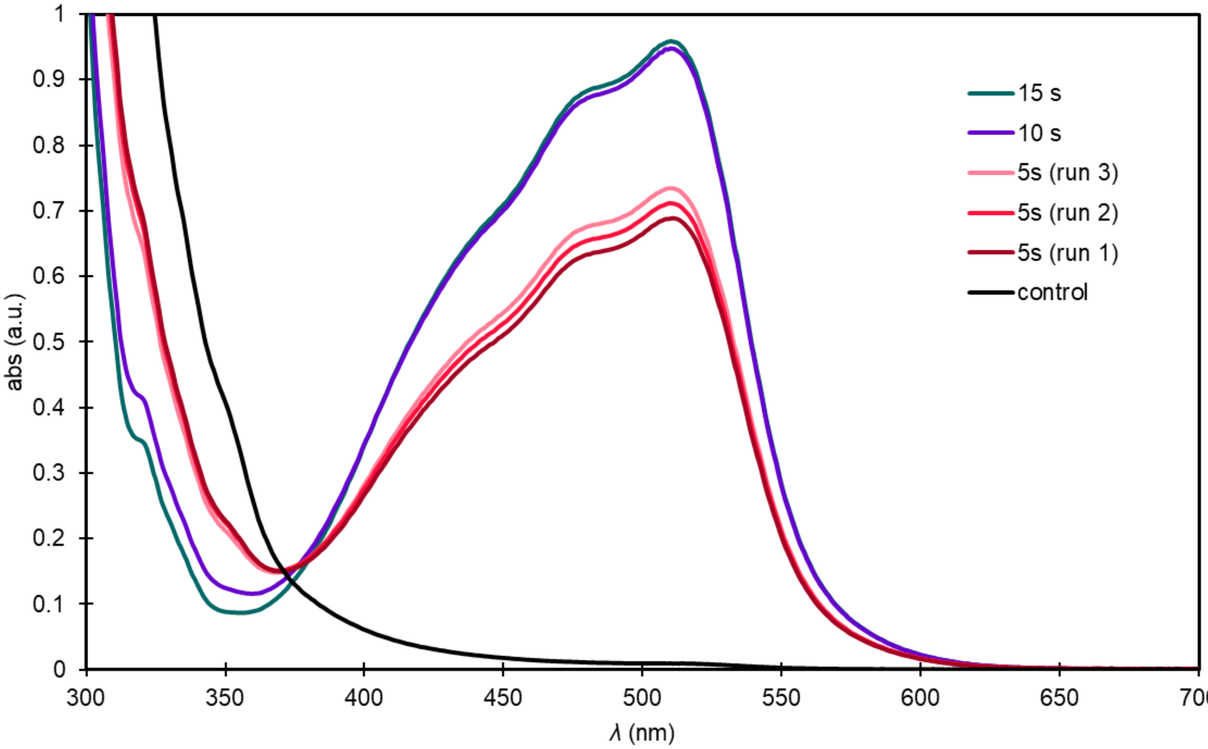

Fig. S91. UV-vis spectra of solutions used for light intensity quantification at different irradiation times.

Because full conversion was not reached after 5 s, but rather sometime between 5 and 10 s, the runs at 5 s were chosen for analysis. These gave an average absorbance of 0.70 ± 0.02 at 510 nm vs. control.

$$I\ (\text{mol} \cdot \text{min}^{-1}) = \frac{AV_2V_3}{\varepsilon b \phi_\lambda t V_1} \quad \text{Eq. S4}$$

where

A = absorbance at 510 nm

V_2 = volume of actinometer irradiated (3 mL)
V_3 = final volume of quantified sample (10 mL)
ε = extinction coefficient of ferrous 1,10-phenanthroline at 510 nm (~1.11 × 10^4 $M^{-1} \cdot cm^{-1}$)
b = path length of cuvette (1 cm)
ϕ_λ = quantum yield of ferrous production at 390 nm (~1.13)
t = time of irradiation (5 s)
V_1 = volume of aliquot of irradiated sample taken (100 μL)

Giving a light intensity in mol of photons per unit time. Using the gathered absorbance data, a light intensity of 2.01 ± 0.01 × 10^{-4} mol · min^{-1} was obtained, and this photon flux was used to calculate relative quantum yield for a given substrate yield over a known irradiation time.

S10. Spectroelectrochemistry of {Fc–N–an}

To confirm our assignment of the feature at 700 nm as belonging to the anthracene radical anion of **{Fc$^+$–NH$^+$–an$^{•-}$}**, we sought to independently generate **{Fc–N–an$^{•-}$}** through spectroelectrochemistry.

A 0.37 mM solution of **{Fc–N–an}** was prepared in THF with 0.7 M [TBA][PF_6] electrolyte, which was chosen for conductivity purposes as DME has a lower solubility for [TBA][PF_6] and lower conductivity.

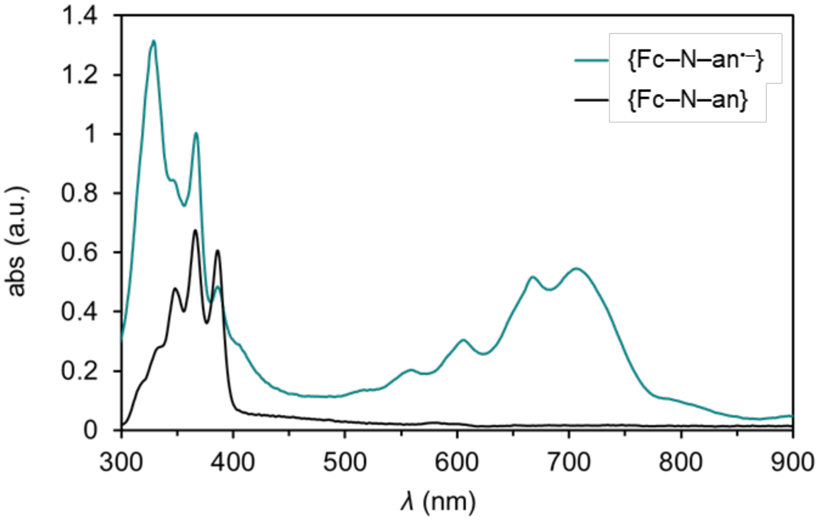

Fig. S92. UV-vis absorption spectra for the electrochemical generation of **{Fc–N–an$^{•-}$}** at -2.4 V vs. $Fc^{+/0}$. The extinction coefficient at 707 nm extracted from these data is 10,000 M^{-1} cm^{-1}. Spectra were taken before (black trace) and after (teal trace) a period of 10 minutes at constant applied potential.

We compared this data with the transient spectrum obtained in section S8. When absorbance-corrected for concentration, the transient spectrum of **{Fc$^+$–NH$^+$–an$^{•-}$}** is blue-shifted by 7 nm from that of **{Fc–N–an$^{•-}$}**.

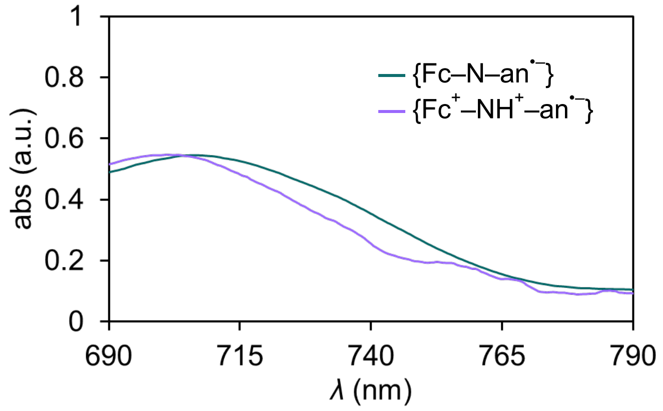

Fig. S93. Overlayed spectra for **{Fc$^+$–NH$^+$–an$^{•−}$}** (purple) generated *via* laser excitation of **{Fc–NH$^+$–an}** in DME with 100 equivalents of [PicH][OTf] and **{Fc–N–an$^{•−}$}** (green) electrochemically generated at -2.4 V vs. Fc$^{+/0}$.

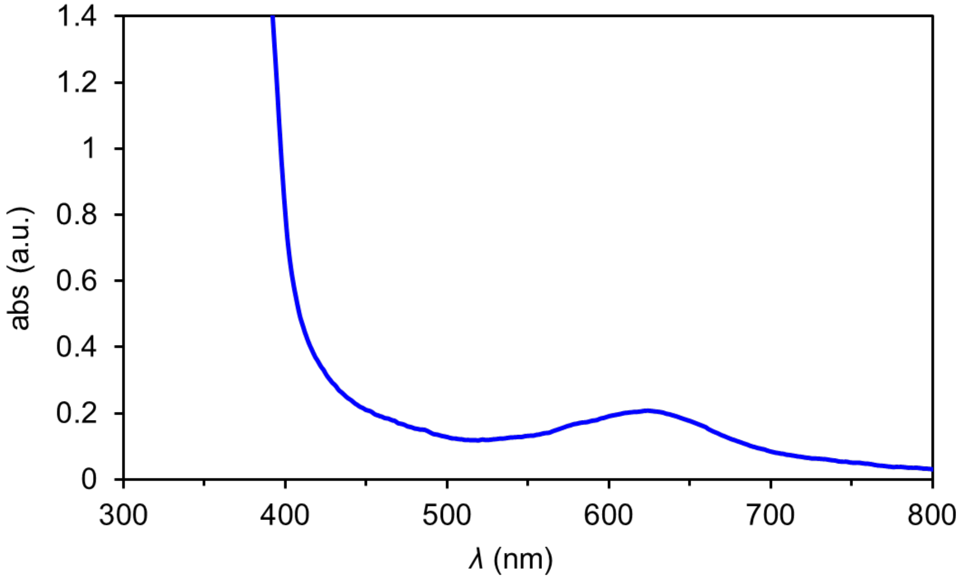

Fig. S94. UV-vis absorption spectrum for the electrochemical generation of **{Fc$^+$–N–an}** (3.7 mM) at + 0.2 V vs. Fc$^{+/0}$.

S11. Isotope scrambling experiment

Based on previous literature,[18] we speculated that upon reductive quenching to generate **{Fc$^+$–NH$^+$–an$^{\bullet-}$}**, the basicity of the anthracenyl radical anion species could be sufficient in the presence of the acids used to generate a neutral anH• species, itself a possible H-atom donor. While our previous control experiments rule out intermolecular protonation (see section S5), intramolecular protonation could still be viable in this case. If intramolecular proton transfer is occurring under irradiation, the back electron transfer (to the Fc$^+$ unit) would be proton-coupled (to the amine N).

If this process is occurring, then if D-acid was used, the re-aromatization would scramble the deuterium atom into the anthracene ring, and this aromatic proton would be detectable by D-NMR. To probe this, a 1 mM solution of **{Fc–N–an}** with 10 mM [PicD][OTf] in DME was irradiated at 390 nm for 1 h in accordance with our stoichiometric photochemical experiments. D-NMR spectra were taken before and after irradiation with a 1 mM C_6D_6 internal standard.

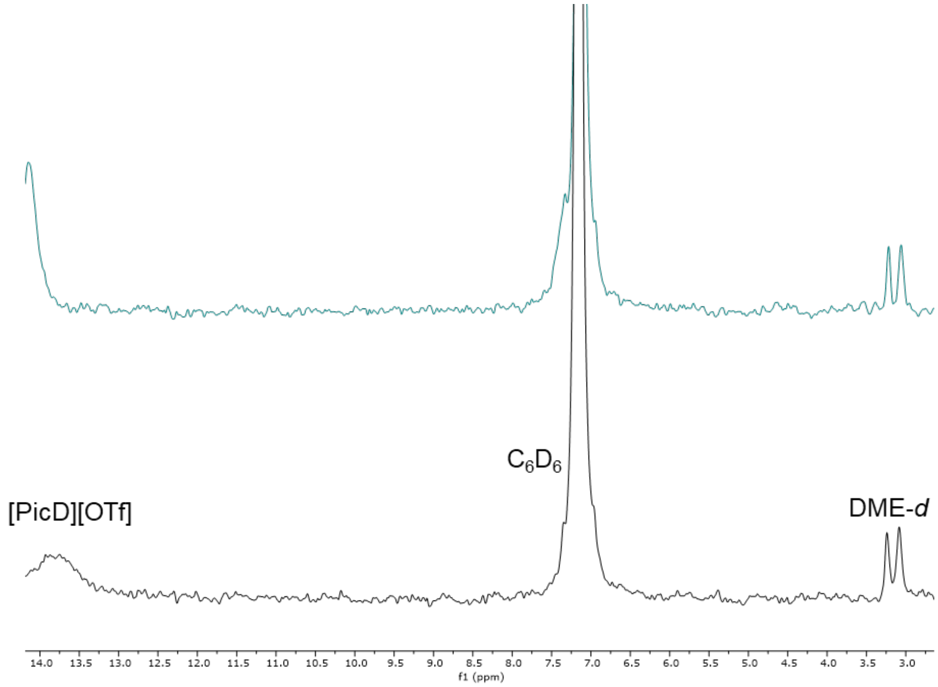

Fig. S95. ^{2}H-NMR (DME, 400 MHz) of a 1 mM solution of **{Fc–N–an}**, 10 mM [PicD][OTf], and a 1 mM C_6D_6 internal standard before (black, bottom) and after (green, top) irradiation at 390 nm for 1 h.

The results of this experiment suggest that there is no scrambling of deuterium into the anthracene ring, and therefore intra- and intermolecular protonation upon reductive quenching are unlikely.

S12. Photoelectrocatalytic reactions

General Controlled Potential Coulometry (CPC) Procedure: Carbon cloth electrodes were cut to 5 cm^2 and used as obtained from the supplier. Controlled potential coulometry (CPC) experiments were carried out in a gas-tight two compartment cell using a carbon cloth (dimensions 1 x 2 cm) working electrode, a Pt wire pseudo-reference electrode, and a carbon cloth (5 cm^2) or Zn foil (10 cm^2) counter electrode. The solution was stirred throughout the CPC. In a typical experiment with an organic substrate, a 6 mL solution of 0.15 M [TBA][PF_6] with two equivalents of acid (based on desired number of H-atom equivalents delivered to substrate) in DME was added to the solution which was added to the electrochemical cell distributing evenly between both compartments. Then, an appropriate amount of substrate (50 mM in 3 mL) and **{Fc–N–an}** (1 mM in 3 mL) was added to the working compartment. After the appropriate reaction time, the solution was quenched with 2 M HCl in ether (1 mL) except for the acid sensitive *N*-phenylbenzaldimine. After 5 min of stirring, the solvent was evaporated under reduced pressure until dryness. The resulting solids were dissolved in diethyl ether (20 mL). For the diphenylfumarate reaction, ethyl acetate (10 mL) was first added to the carbon cloth working electrode, then sonicated to extract the remaining starting material (sparingly soluble in diethyl ether) before 100 mL of diethyl ether was added. The solution was subsequently washed with either 1 M HCl (aq, acetophenone), sat. $NaHCO_3$ (aq, *N*-phenylbenzaldimine), or 0.1 M Na_2CO_3 (aq, diphenylfumarate) solution (20 mL) and extracted with ether (5 x 20 mL). The organic layers were combined, dried over Na_2SO_4, and concentrated in vacuo. A crude ^{1}H-NMR was taken with CH_2Br_2 as internal standard. In reactions where ferrocene oxidation was used as the anodic reaction, an amount of ferrocene equivalent to that of the acid in the working compartment was dissolved in the counter compartment. Upon completion of the CPC, the counter compartment is a deep blue color, indicating the formation of Fc^+.

Because of the photo-induced reduction of Ag^+ to form Ag^0 nanoparticles, we opted to use a Pt-wire pseudo-reference that was calibrated from the Fe(III/II) couple of **{Fc–NH$^+$–an}** in a CV before electrolysis.

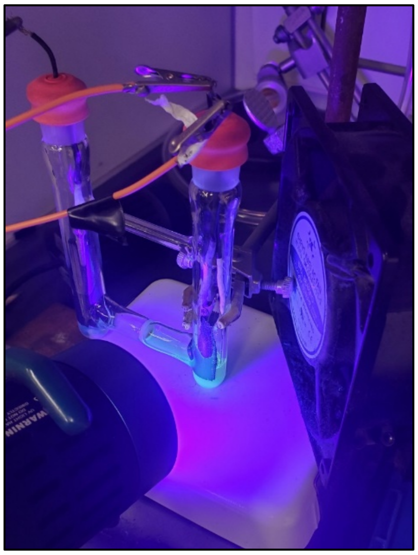

Fig. S96. Photoelectrocatalysis setup as described, with a two compartment electrochemical cell separated by a fine pore frit, 390 nm Kessil H150 lamp, and fan cooling.

Photoelectrocatalytic acetophenone reduction

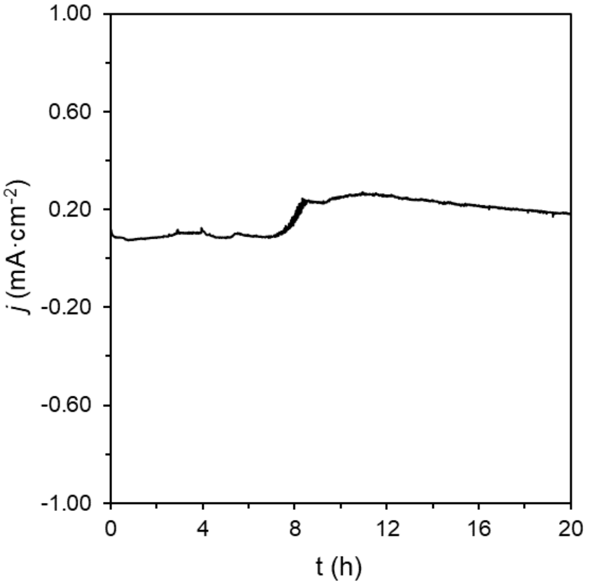

Fig. S97. Current vs. time bulk electrolysis profile for the photoelectrocatalytic reduction of acetophenone at a constant applied potential of -0.1 V vs. $Fc^{+/0}$ under 390 nm irradiation.

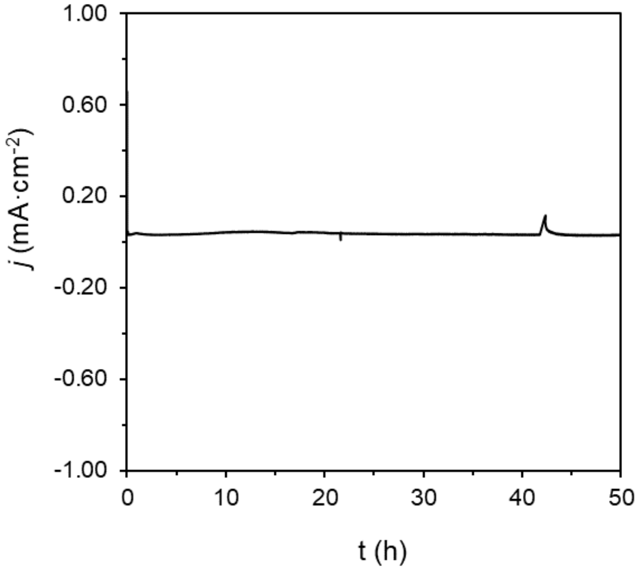

Fig. S98. Current vs. time bulk electrolysis profile for the photoelectrocatalytic reduction of acetophenone at a constant applied potential of -0.1 V vs. $Fc^{+/0}$ under 390 nm irradiation where ferrocene oxidation to ferrocenium was employed as the anodic reaction.

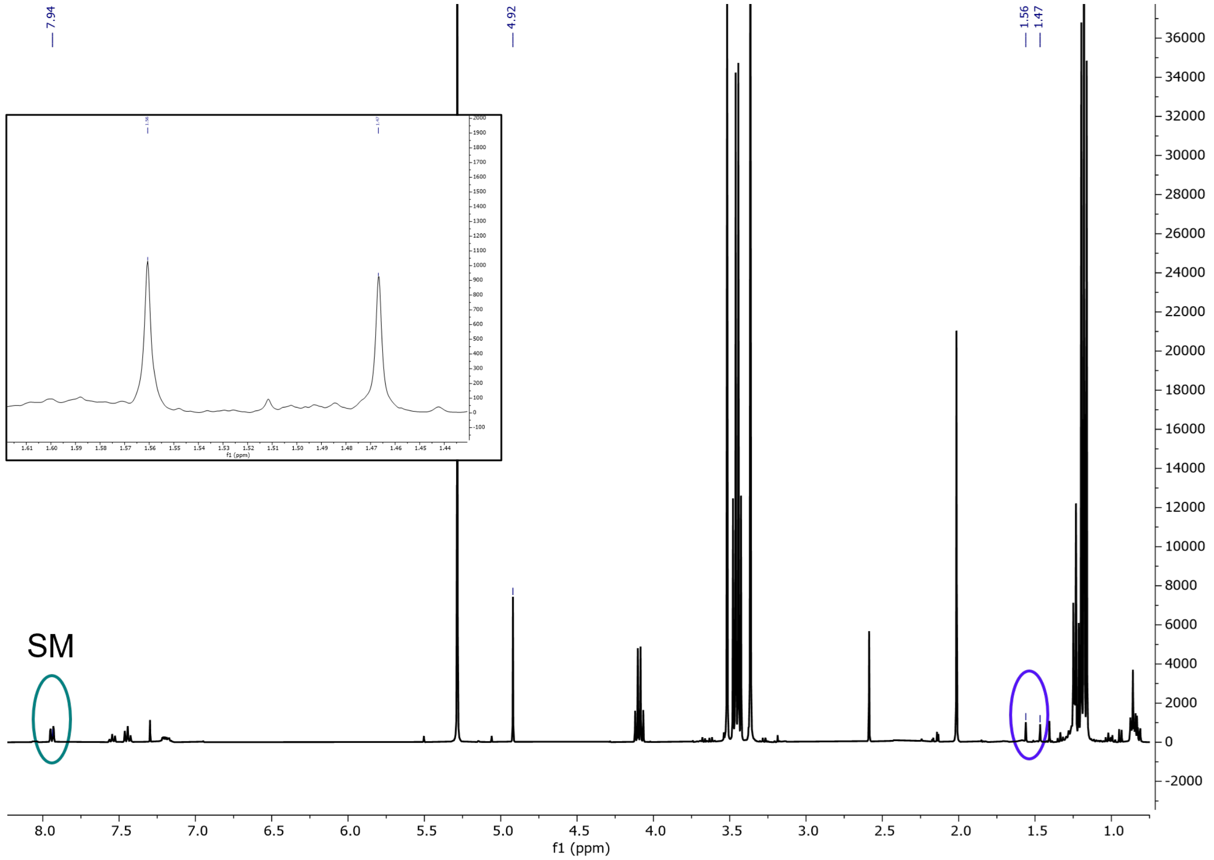

Fig. S99. ^{1}H-NMR (400 MHz, $CDCl_3$) quantification for acetophenone reduction using a 0.15 mmol CH_2Br_2 standard.

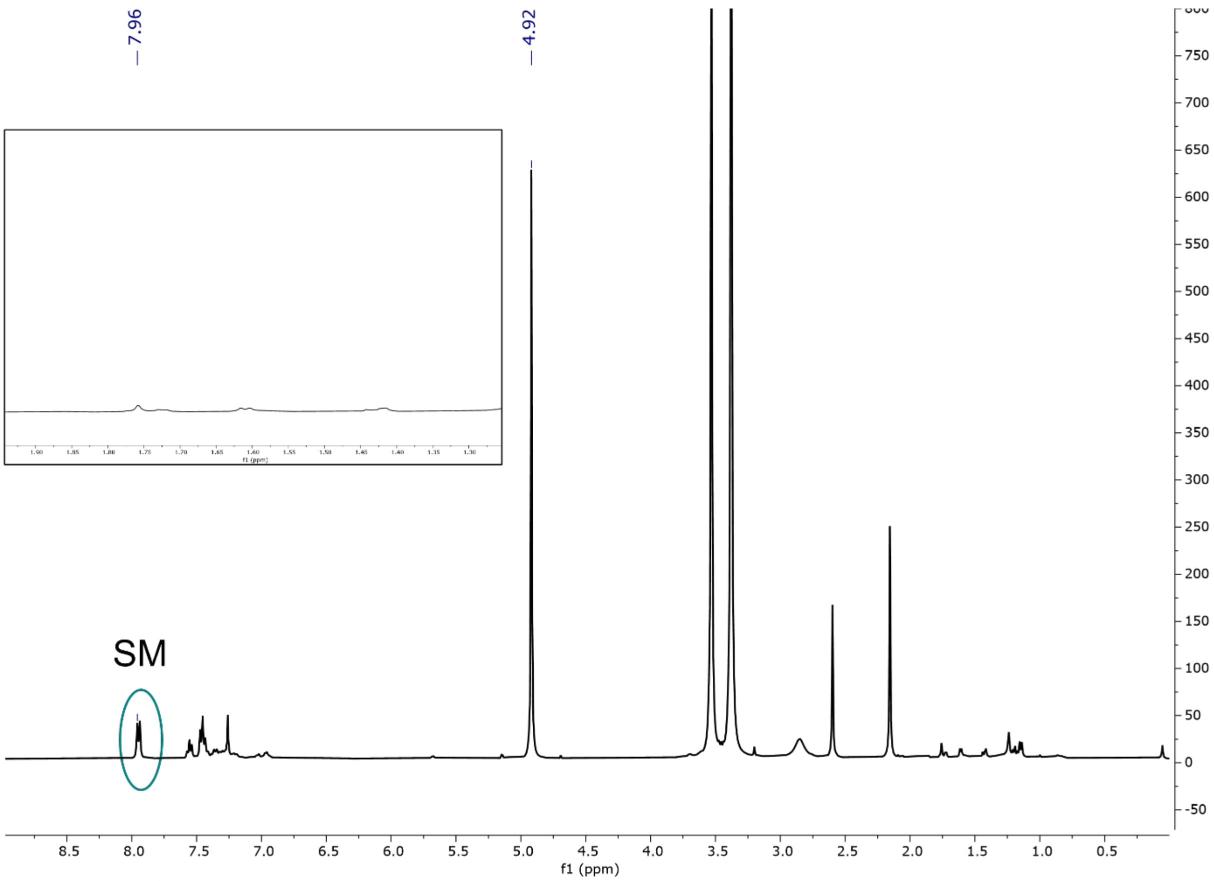

Fig. S100. ^{1}H-NMR (400 MHz, $CDCl_3$) quantification for pinacol formation in the absence of **{Fc–N–an}**, using a 0.15 mmol CH_2Br_2 standard.

Table S1. Bulk electrolyses for acetophenone reduction using **{Fc–N–an}** while irradiating at 390 nm and at a constant applied potential of -0.1 V vs $Fc^{+/0}$ unless otherwise specified. All electrolyses were conducted in DME with carbon cloth working electrode, zinc foil counter electrode, and a platinum pseudo-reference. SM = starting material. [a] Ferrocene oxidation was used as the anodic reaction. [b] Boron-doped diamond working electrode.

[acetophenone]	{Fc–N–an}	[PicHOTf]	conversion	TONs	% SM
50 mM	2 mol %	100 mM	36 ± 4 %	18 ± 2	53 ± 1 %
50 mM	2 mol %	100 mM	30 %[a]	15	69 %
50 mM	NONE[b]	50 mM	0 %	0	79 %
50 mM	2 mol %; **NO LIGHT**	50 mM	0 %	0	63 %

Diphenylfumarate (DPF) and N-phenylbenzaldimine reduction

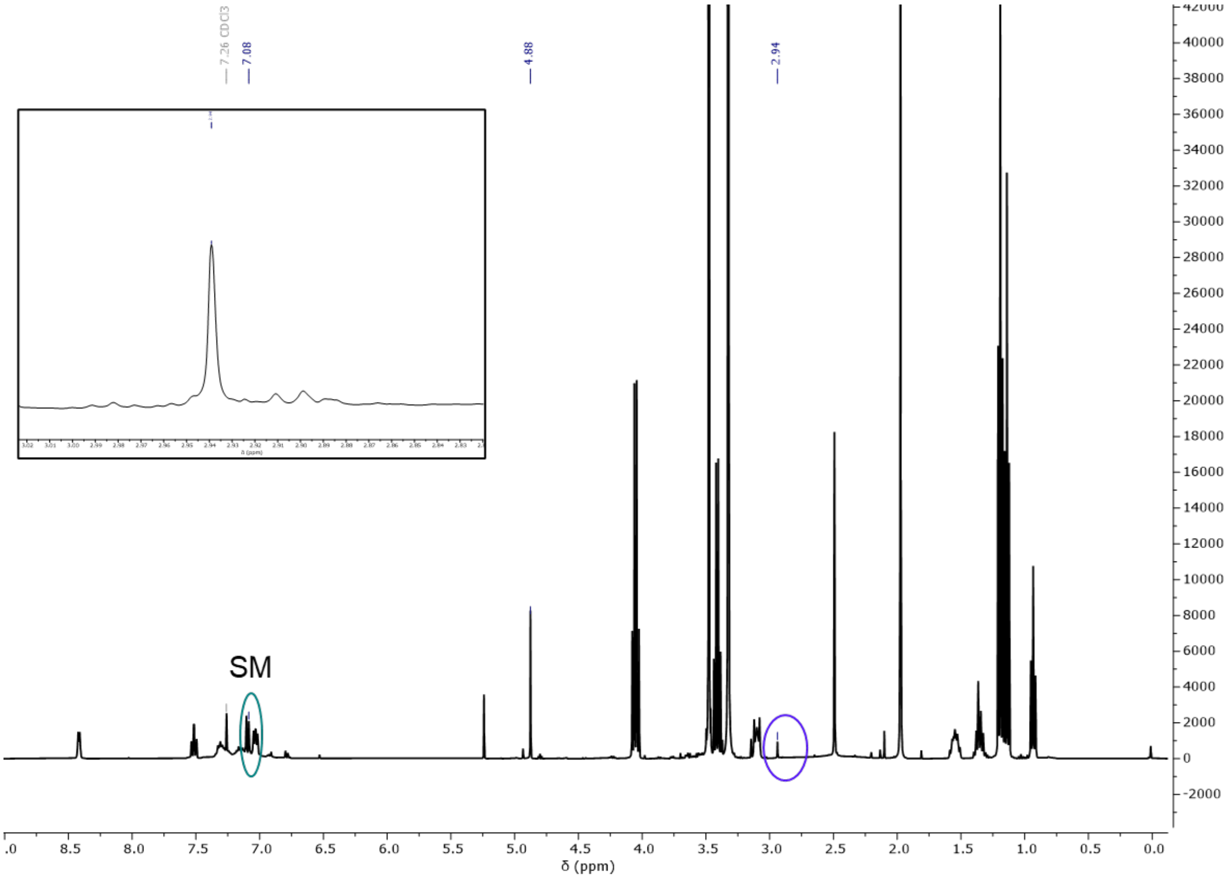

Fig. S101. ^{1}H-NMR (400 MHz, $CDCl_3$) quantification for diphenylfumarate reduction using a 0.15 mmol CH_2Br_2 standard.

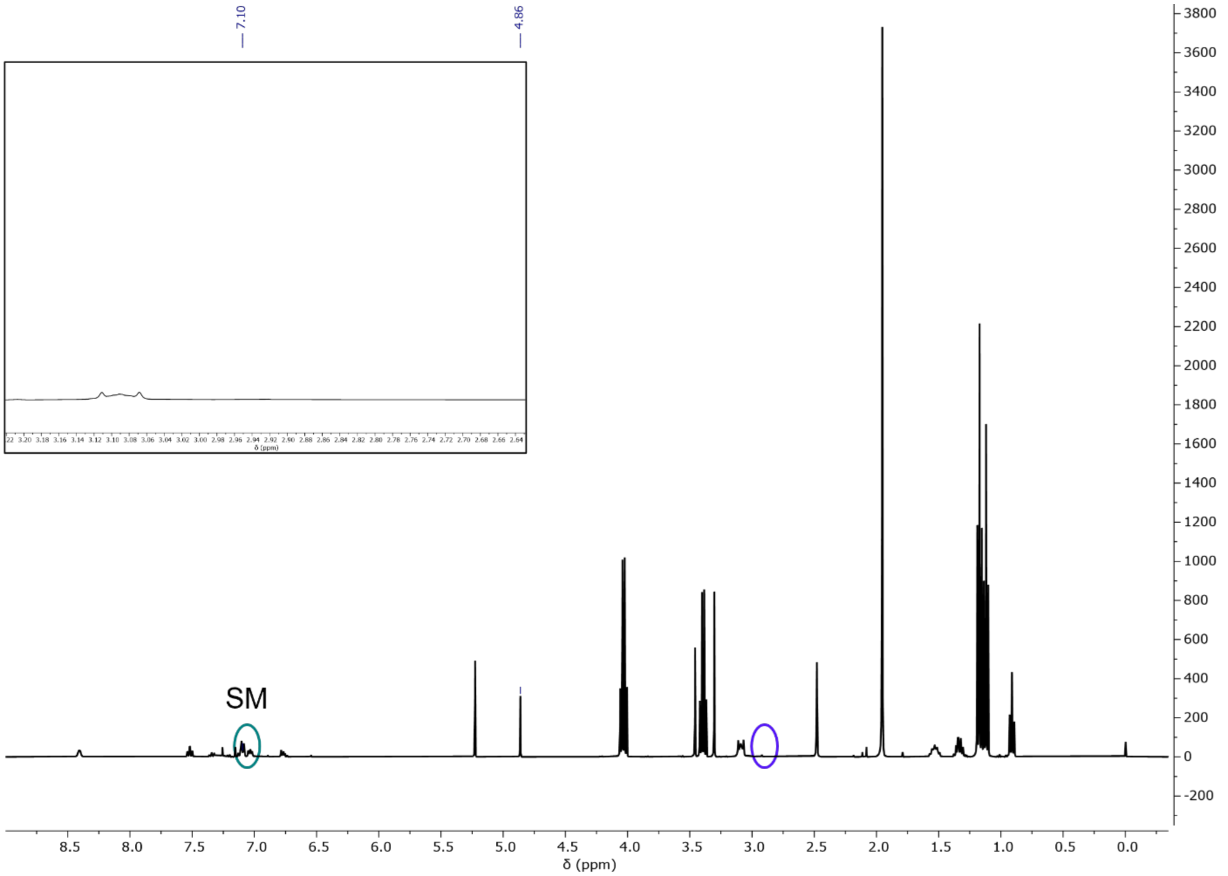

Fig. S102. ^{1}H-NMR (400 MHz, $CDCl_3$) quantification for diphenylsuccinate formation in the absence of **{Fc–N–an}** using a 0.15 mmol CH_2Br_2 standard.

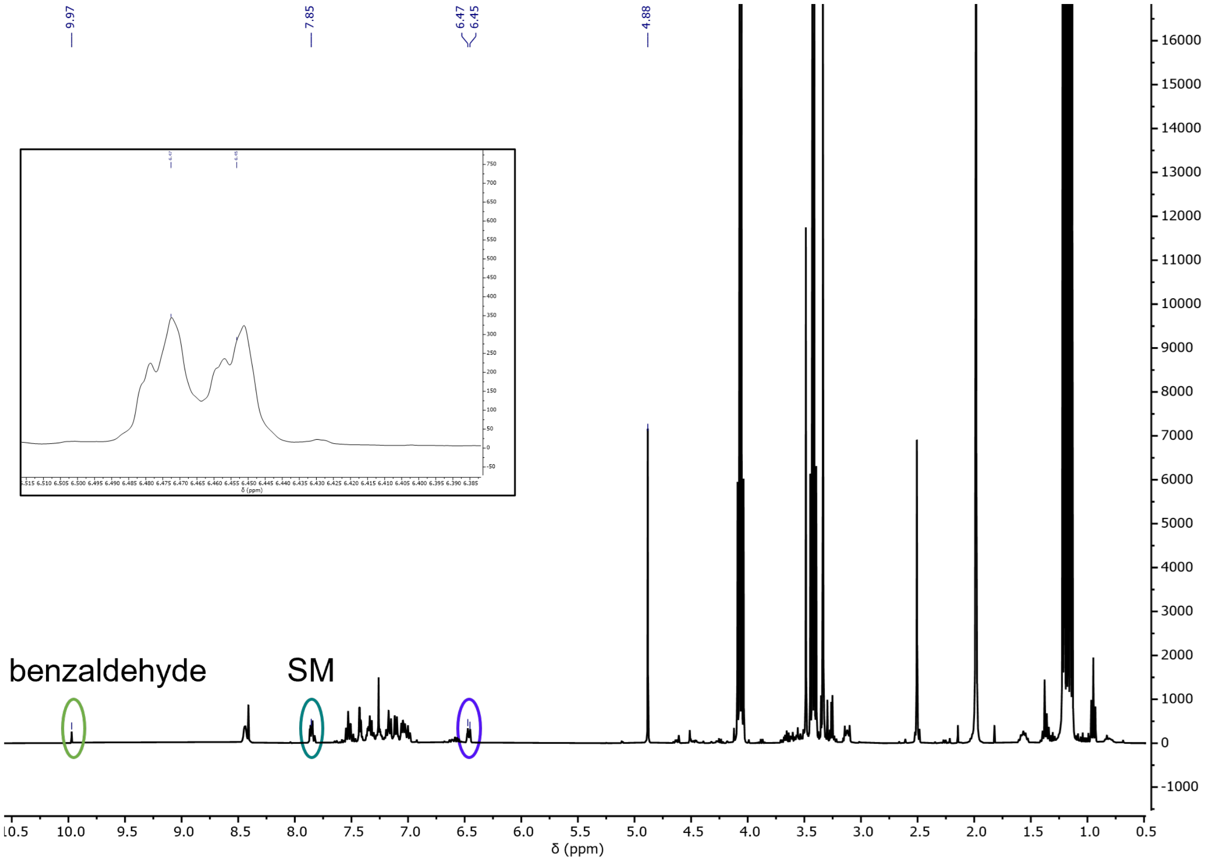

Fig. S103. ^{1}H-NMR (400 MHz, $CDCl_3$) quantification for N-phenylbenzaldimine reduction using a 0.15 mmol CH_2Br_2 standard. Benzaldehyde is formed by hydrolysis of N-phenylbenzaldimine during aqueous workup.

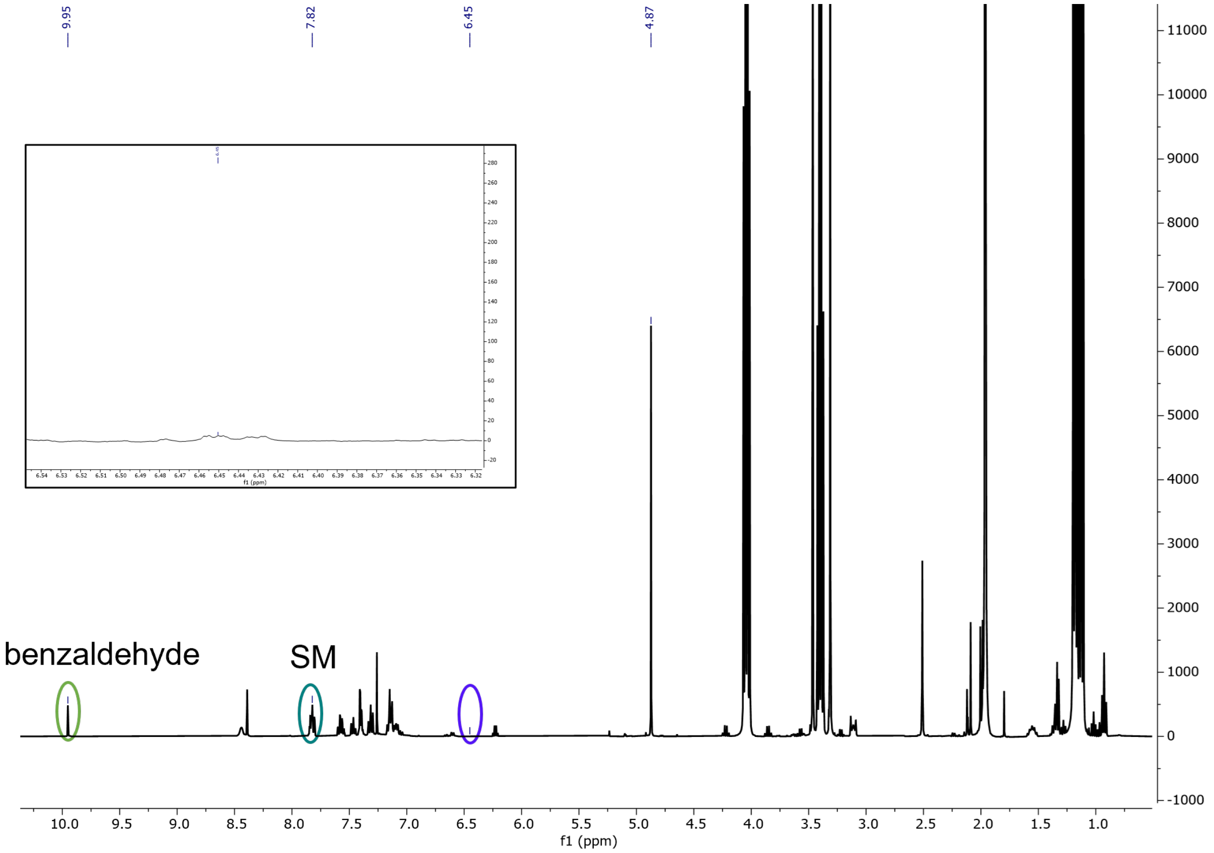

Fig. S104. ^{1}H-NMR (400 MHz, $CDCl_3$) quantification for aza-pinacol formation in the absence of **{Fc–N–an}** using a 0.15 mmol CH_2Br_2 standard. Benzaldehyde is formed by hydrolysis of N-phenylbenzaldimine during aqueous workup.

Table S2. Bulk electrolyses using **{Fc–N–an}** while irradiating at 390 nm and at a constant applied potential of -0.1 V vs $Fc^{+/0}$ unless otherwise specified. All electrolyses were conducted in DME with carbon cloth working electrode, zinc foil counter electrode, and a platinum pseudo-reference. SM recovery for N-phenylbenzaldimine includes benzaldehyde formed by hydrolysis of SM during aqueous workup.

Substrate (50 mM)	{Fc–N–an}	[PicHOTf]	conversion	TONs	% SM
DPF	2 mol %	50 mM	9 %	9	75 %
DPF	**NONE**	50 mM	0 %	0	92 %
N-phenylbenzaldimine	2 mol %	50 mM	30 %	10	55 %
N-phenylbenzaldimine	**NONE**	50 mM	0 %	0	82 %

S13. H_2 Quantification for CPE

For the photoelectrocatalytic reduction of acetophenone, the headspace was analyzed for the presence of H_2 and we were not able to detect any H_2 formed in the reaction.

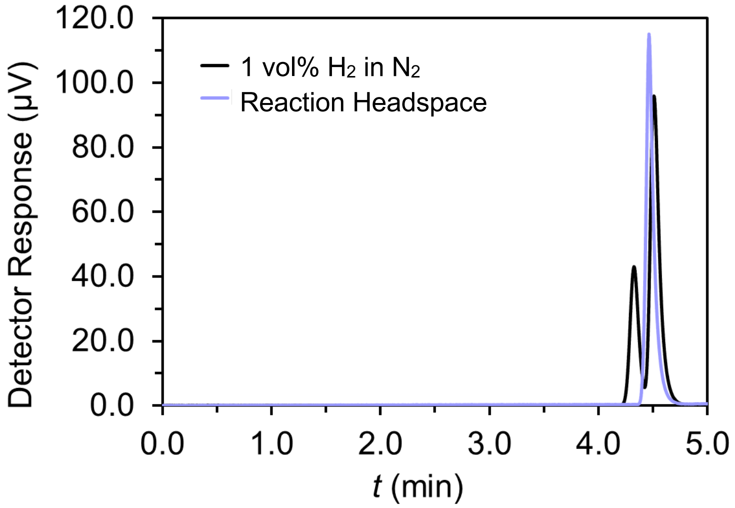

Fig. S105. GC-TCD chromatograms observed for detection of H_2 after CPE at-0.1 V vs. $Fc^{+/0}$ of a DME solution containing 50 mM acetophenone, 100 mM [PicH][OTf] and, 0.15 M $TBAPF_6$ irradiated at 390 nm for 24 h. No H_2 was detected, only the N_2 atmosphere, compared with a 1 vol % sample of H_2 in N_2 showcasing an H_2 retention time of 4.3 min and an N_2 retention time of 4.5 min.

S14. Stoichiometric reactions with inorganic substrates

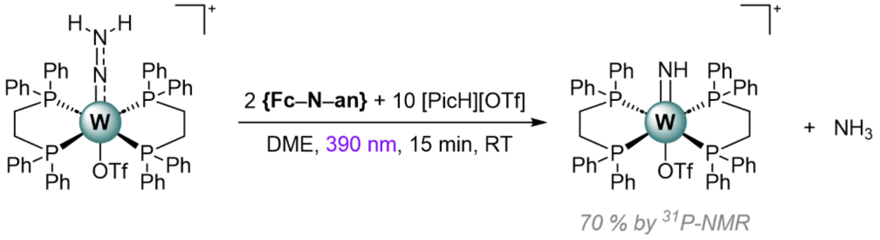

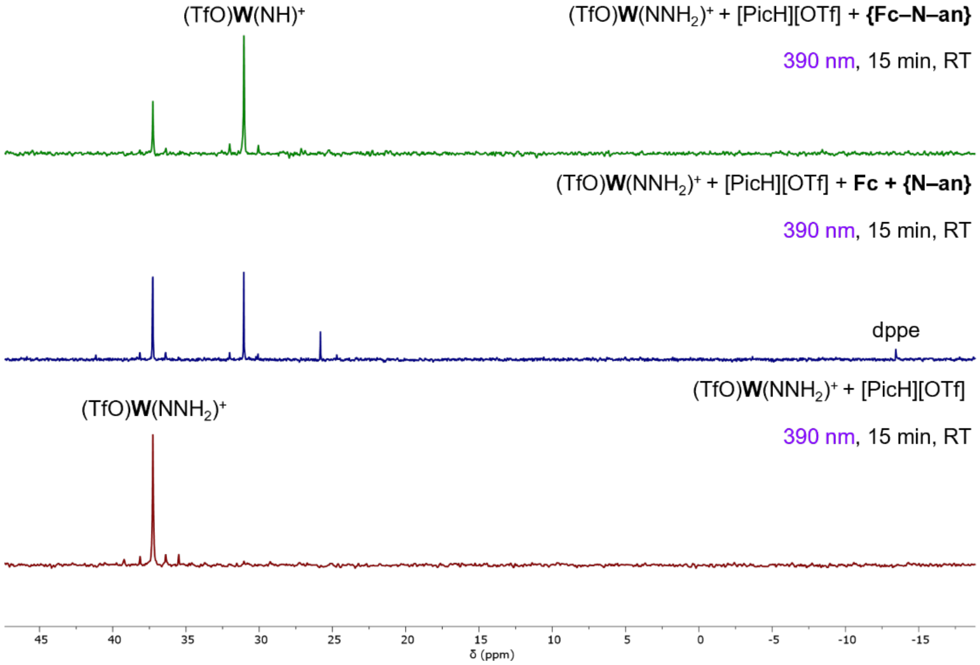

Fig. S106. ^{31}P-NMR (DME) on photochemical PCET to (TfO)**W**(NNH_2)$^+$ using **{Fc–N–an}** with control experiments comparing before and after irradiation, showing conversion of (TfO)**W**(NNH_2)$^+$ to 70 % (TfO)**W**(NH)$^+$ *via p*PCET in the presence of **{Fc–N–an}**. After irradiation, O=P($OPh)_3$ was added as an internal P standard. The identity of (TfO)**W**(NH)$^+$ was determined by comparison with previous studies.[2] In control experiments, no (TfO)**W**(NH)$^+$ was observed in the absence of **{Fc–N–an}**, and 35 % was observed when **{N–an}** and **Fc** were used in place of the **{Fc–N–an}**. [$^{15}NH_4$][OTf] was also similarly observed when the ^{15}N labeled complex was used, indicating that although proton and electron transfer are possible from the bimolecular mixture of **{N–an}** and **Fc**, there appears to be at least a kinetic advantage to covalently coupling the photosensitizer, Brønsted acid, and quencher as the yield doubles when **{Fc–N–an}** is used instead.

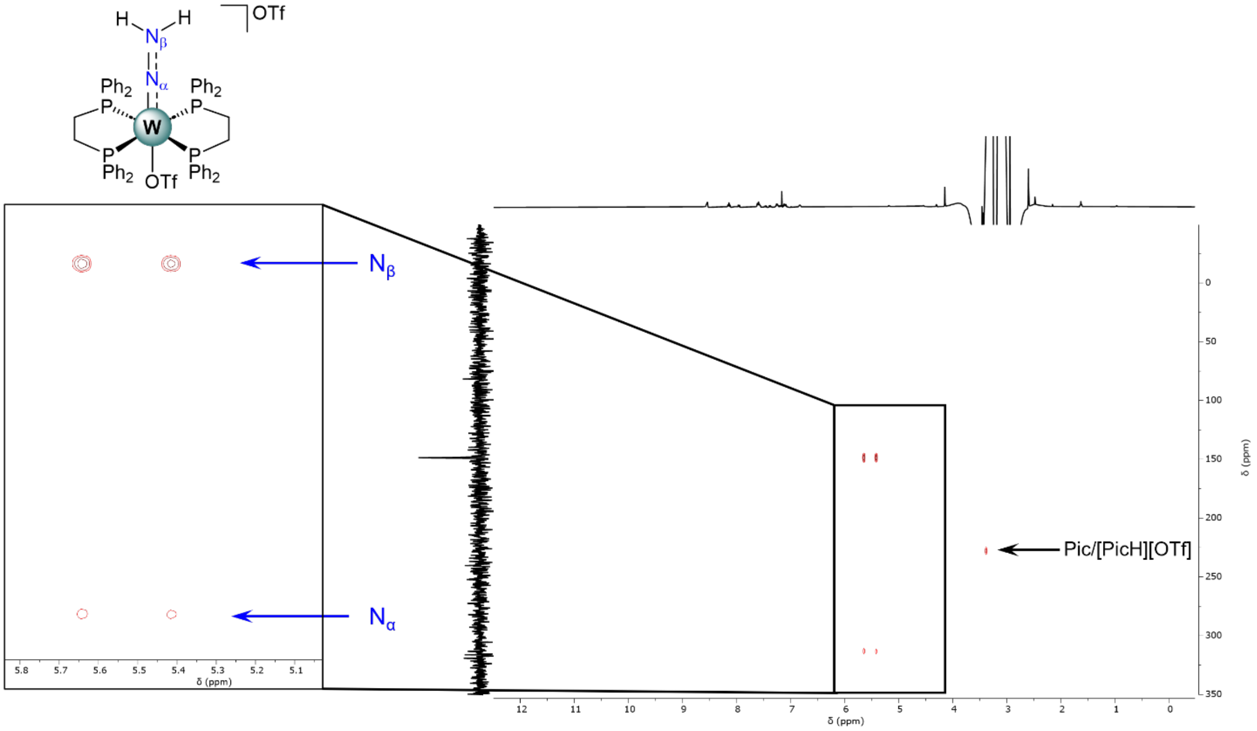

Fig. S107. ^1H–^{15}N HMBC (400 MHz) of (TfO)**W**($^{15}N^{15}NH_2$)$^+$ (2.6 mM) in DME with 2 equivalents **{Fc–N–an}** and 10 equivalents [PicH][OTf]. $^{15}N_\beta$–H_2: (5.5, 150.3, J = 92 Hz), $^{15}N_\alpha$–H_2: (5.53, 314.4).

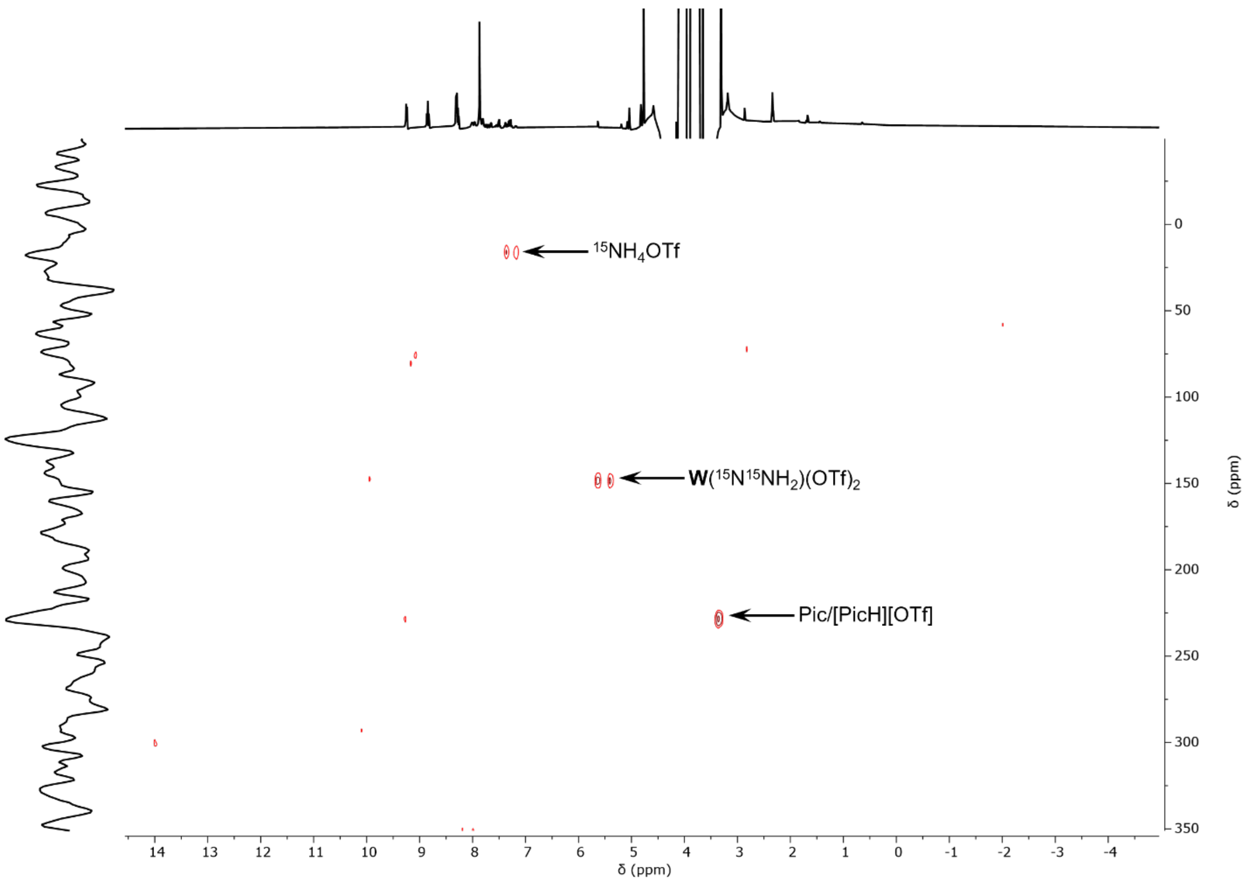

Fig. S108. ^{1}H–^{15}N HMBC (400 MHz) of (TfO)**W**($^{15}N^{15}NH_2$)$^{+}$ (1 mM) in DME with 2 equivalents **{Fc–N–an}** and 10 equivalents [PicH][OTf] after irradiation at 390 nm for 15 minutes. $^{15}N_{\beta}$–H_2: (5.5, 150.3, J = 92 Hz), $^{15}NH_4OTf$: (7.3, 17.8, J = 72 Hz).

The observation of $^{15}NH_4OTf$ in this experiment is consistent with PCET from **{Fc^{+}–NH^{+}–an$^{\bullet-}$}** to (TfO)**W**($^{15}N^{15}NH_2$)$^{+}$ followed by N–N bond cleavage to form (TfO)**W**(NH)$^{+}$. The observation of (TfO)**W**(^{15}NH)$^{+}$ by this method depends heavily on the degree of coupling between the ^{15}N–H. While imides on this platform have been observed by ^{15}N-NMR,[19] the N**H** proton is often lost in ^{1}H-NMR,[20] making its detection difficult by this method. Its formation in this experiment was instead confirmed by ^{31}P-NMR.

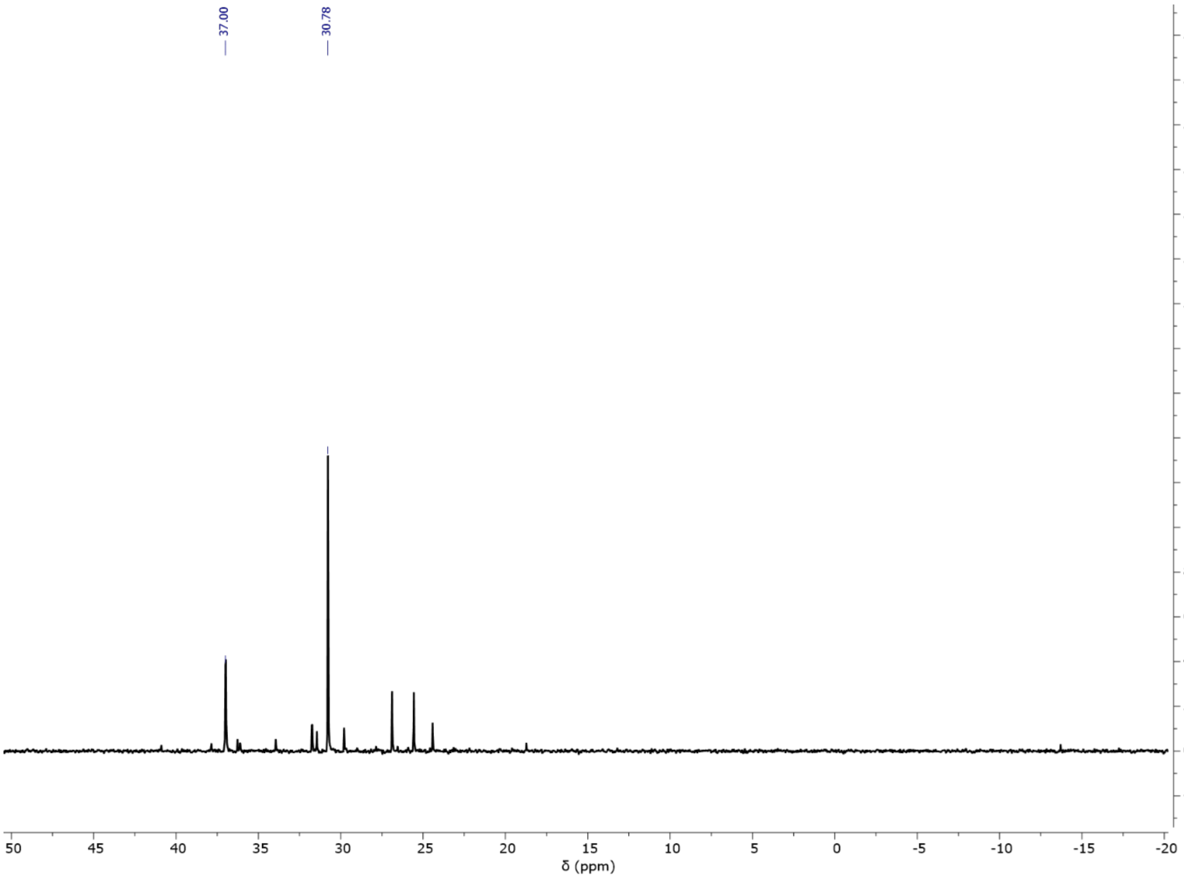

Fig S109. ^{31}P-NMR (DME) on photochemical PCET to (TfO)**W**(^{15}N^{15}NH$_2$)$^+$ using **{Fc–N–an}**, showing conversion)$^+$ to (TfO)**W**(^{15}NH)$^+$ *via p*PCET in the presence of **{Fc–N–an}**.

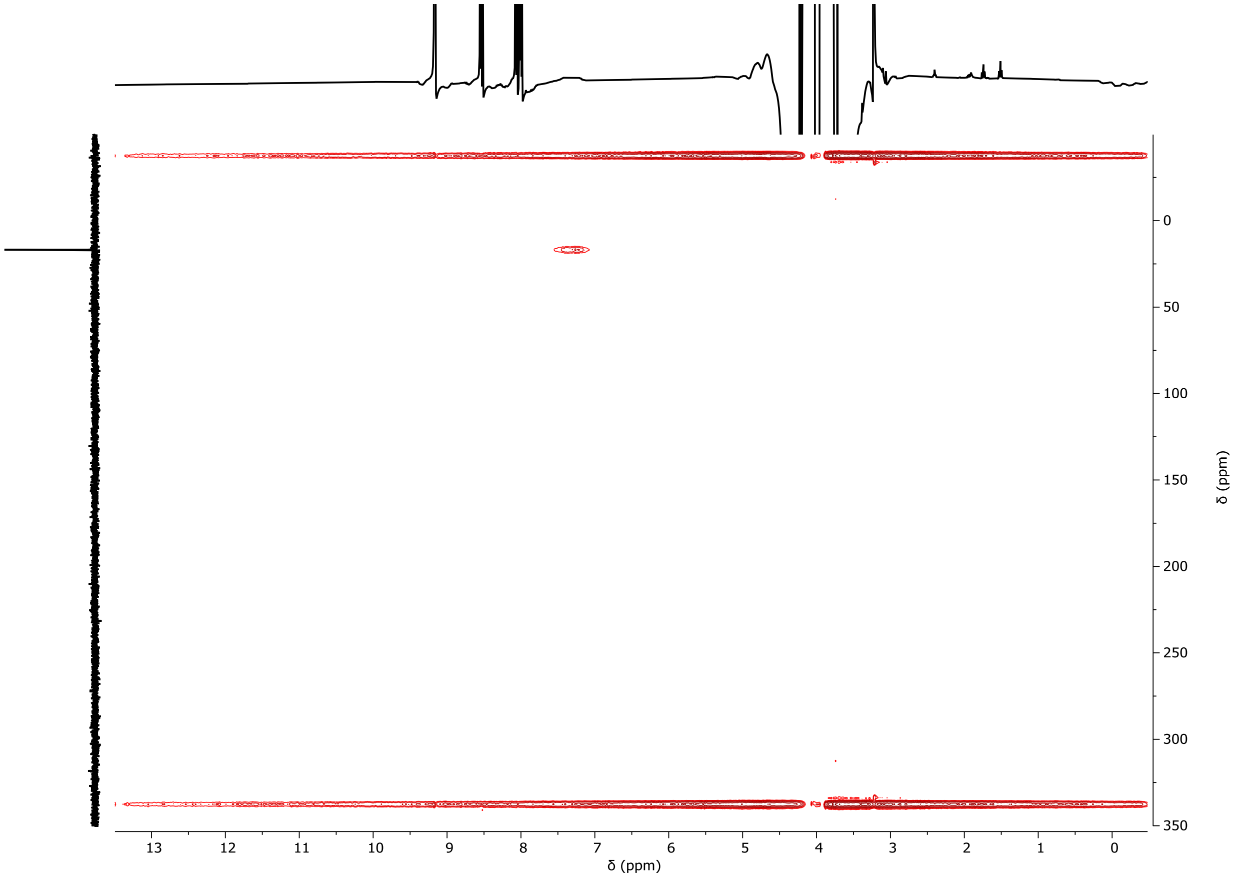

Fig. S110. ^{1}H–^{15}N HSQC (400 MHz) of $^{15}NH_4OTf$ in DME with a 3:2 mixture of Pic:[PicH][OTf] to mimic the proton environment of the $^{15}NH_4OTf$ formed post-irradiation. $^{15}NH_4OTf$: (7.3, 17.1).

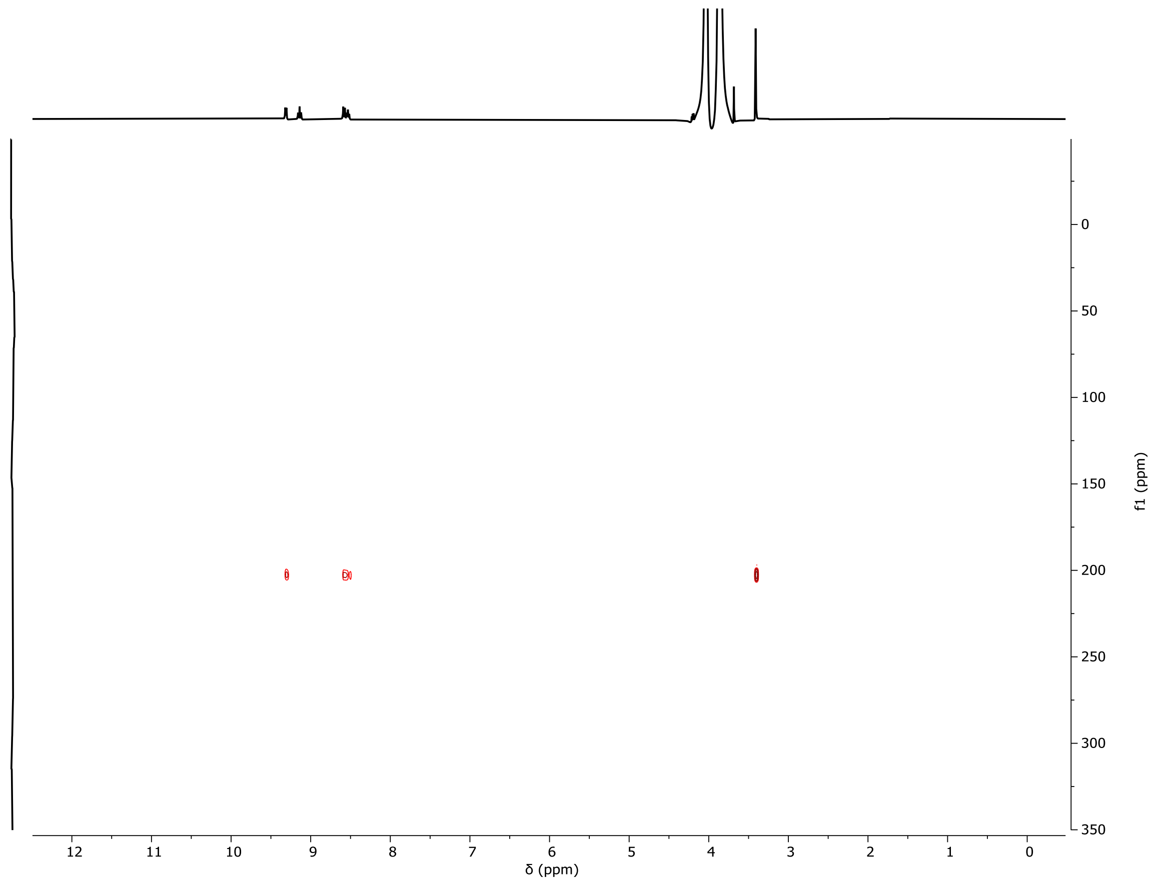

Fig. S111. ^{1}H–^{15}N HMBC (400 MHz) of [PicH][OTf] in DME. (3.4, 202.4); (8.56, 202.4); (9.3, 202.4).

S16. DFT calculations

TD-DFT Ferrocene and other calculations

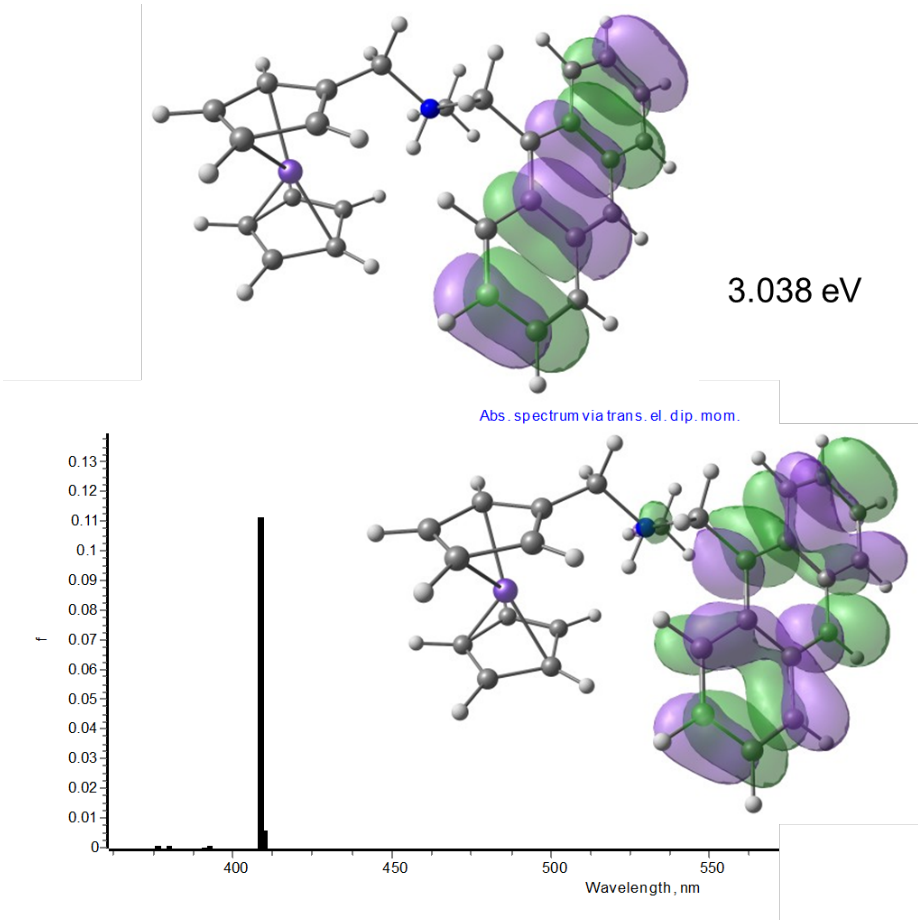

Fig. S112. TD-DFT calculation of the **{Fc–N–an}** species together with the representation of the orbitals involved in the transition observed at approximately 410 nm. The top structure is the occupied source orbital while the bottom structure represents the receiving empty orbital. Both are anthracene-centered orbitals, reflecting the π-π* transition.

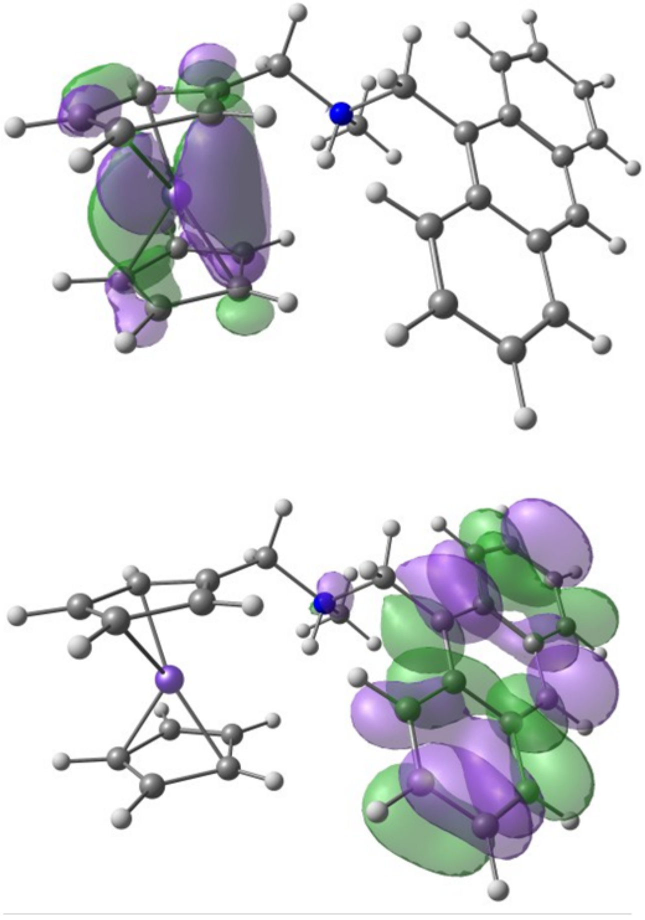

Fig. S113. DFT optimized structure of the **{Fc–N–an}** species and representation of the HOMO (top) and LUMO (bottom) orbitals, associated to the overall charge transfer process responsible for the formation of the excited-state PCET donor.

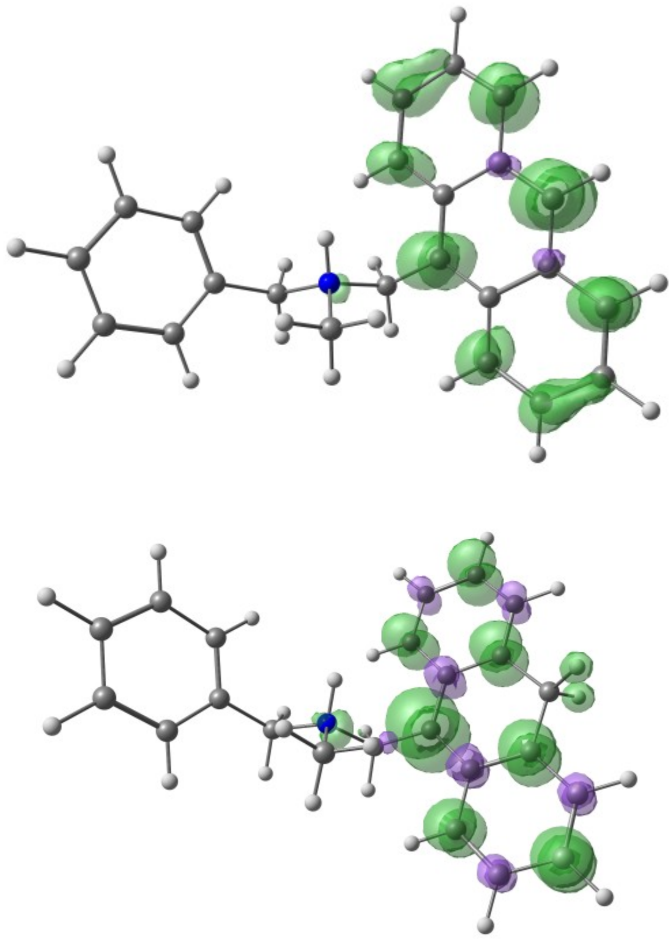

Fig. S114. DFT optimized structure of **{N−an$^{\bullet-}$}** (top) and **{NH^{+}−an$^{\bullet-}$}** (bottom) with the representation of the spin density in both cases.

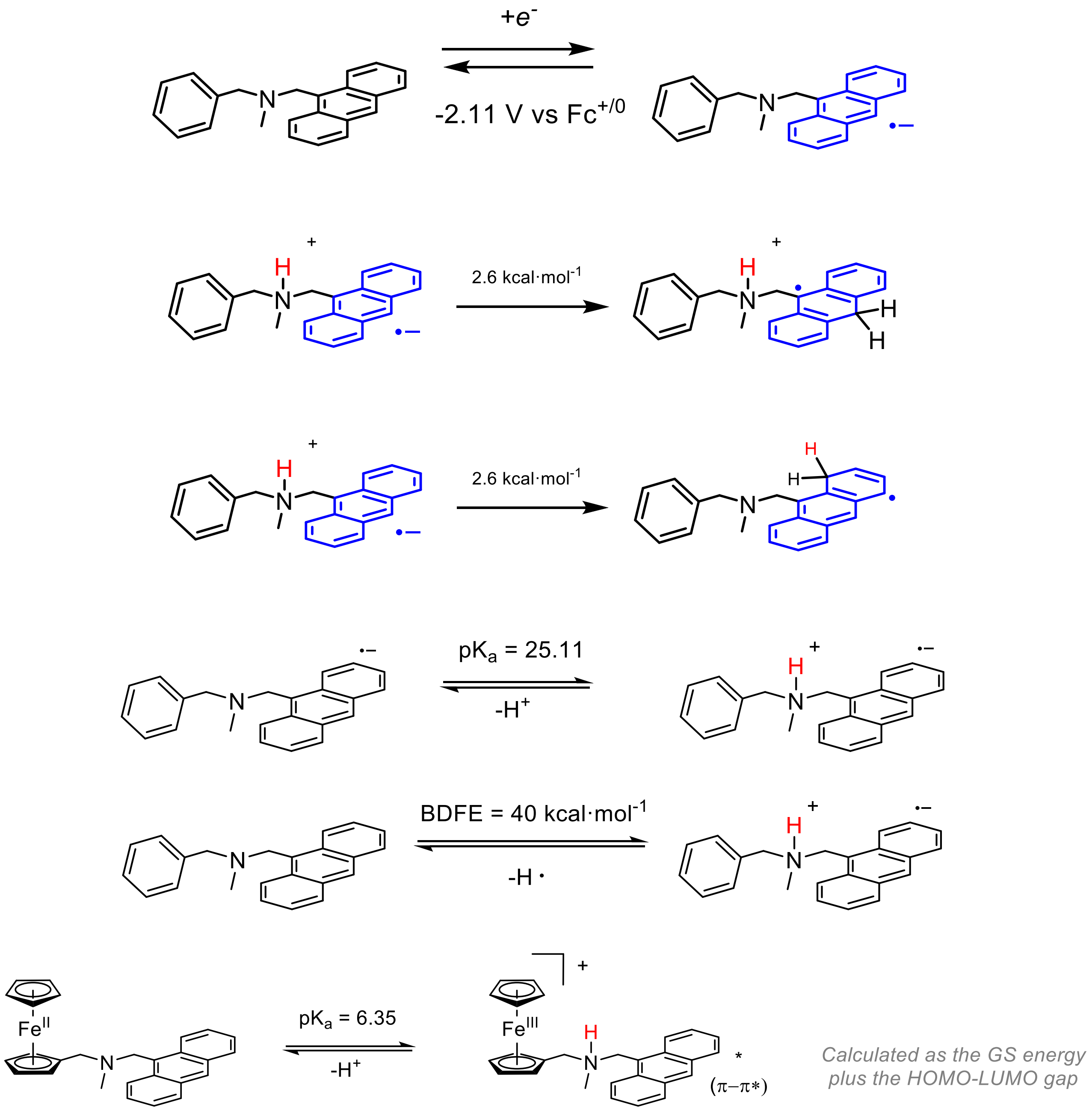

Fig. S115. Schematic representation of the potential reactions via DFT involving reduction and protonation of the anthracene fragment calculated using **{N–an}** or **{Fc–N–an}** as model platforms.

From our calculated $BDFE_{X\text{-}H}$ values, we have noticed a deviation from experimentally obtained energies of ~3 kcal·mol^{-1} and have adjusted our values to reflect this discrepancy.[21]

XYZ coordinates

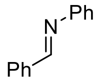

E= -557.102213565203 H

C	-0.304236000	0.695996000	-0.000718000
H	0.130908000	-0.310980000	-0.001897000
C	-1.764896000	0.735477000	-0.000380000
C	-2.471662000	-0.479777000	-0.001540000
C	-2.484336000	1.945655000	0.001088000
C	-3.865307000	-0.489231000	-0.001241000
H	-1.916697000	-1.415144000	-0.002671000
C	-3.873981000	1.932936000	0.001379000
H	-1.938040000	2.884204000	0.001991000
C	-4.569261000	0.716666000	0.000218000
H	-4.401716000	-1.433944000	-0.002145000
H	-4.423802000	2.870161000	0.002519000
H	-5.655767000	0.712596000	0.000456000
C	1.819692000	1.719032000	0.000194000
C	2.470320000	2.964569000	-0.001303000
C	2.603683000	0.547987000	0.001660000
C	3.861557000	3.047352000	-0.001488000
H	1.859255000	3.863282000	-0.002364000
C	3.992752000	0.636175000	0.001505000
H	2.137594000	-0.432520000	0.003027000
C	4.630203000	1.882036000	-0.000093000
H	4.344281000	4.020867000	-0.002699000
H	4.585502000	-0.274884000	0.002675000
H	5.715023000	1.939800000	-0.000190000
N	0.408738000	1.768797000	0.000221000

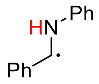

E= -557.691735760163 H

C	-1.765740000	0.723851000	0.031074000
C	-2.522261000	-0.482656000	0.115853000
C	-2.496305000	1.943901000	-0.083958000
C	-3.906936000	-0.468570000	0.086726000
H	-1.988310000	-1.426232000	0.204981000
C	-3.885086000	1.940793000	-0.111128000
H	-1.976105000	2.895581000	-0.157014000
C	-4.608819000	0.743601000	-0.026790000
H	-4.452793000	-1.406551000	0.153667000
H	-4.415026000	2.886085000	-0.201263000
H	-5.694566000	0.753030000	-0.049572000
C	1.862778000	1.745106000	0.004004000
C	2.511688000	2.996314000	-0.015093000
C	2.640486000	0.570447000	0.016046000
C	3.899248000	3.069997000	-0.017704000
H	1.910546000	3.902945000	-0.026768000
C	4.031378000	0.663785000	0.014542000
H	2.171604000	-0.407577000	0.020617000
C	4.674747000	1.903896000	-0.001285000
H	4.379090000	4.045038000	-0.032366000
H	4.617998000	-0.251273000	0.023856000
H	5.759049000	1.962420000	-0.002352000
N	0.471607000	1.730654000	0.007895000
H	0.038531000	2.648462000	-0.029736000
C	-0.352608000	0.642104000	0.063216000
H	0.123607000	-0.326564000	0.143875000

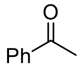

E= -385.135876025422 H

C	-2.001916000	0.578142000	0.003300000
C	-0.607527000	0.542249000	0.004778000
C	0.134472000	1.734745000	0.001875000
C	-0.553561000	2.961778000	-0.002340000
C	-1.942864000	2.996740000	-0.004290000
C	-2.671856000	1.802280000	-0.001486000
H	-2.564799000	-0.350909000	0.005820000
H	-0.107535000	-0.419894000	0.008991000
H	0.024687000	3.880546000	-0.004387000
H	-2.462253000	3.951013000	-0.008005000
H	-3.758112000	1.827726000	-0.002975000
C	1.629827000	1.767670000	0.003654000
C	2.435643000	0.485764000	-0.002090000
H	3.078384000	0.481764000	0.885199000
H	1.843615000	-0.428860000	-0.014789000
H	3.091210000	0.497994000	-0.879786000
O	2.222702000	2.847410000	0.009483000

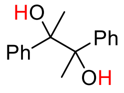

E= -771.469416353301 H

C	-6.153173000	0.218695000	0.966539000
C	-4.787803000	0.324254000	1.234024000
C	-3.878086000	0.643036000	0.215060000
C	-4.376071000	0.856168000	-1.076950000
C	-5.742282000	0.751891000	-1.346956000
C	-6.637870000	0.431009000	-0.326245000
H	-6.839983000	-0.028982000	1.771904000
H	-4.430172000	0.148276000	2.242302000
H	-3.685209000	1.105425000	-1.875653000
H	-6.104639000	0.921213000	-2.357800000
H	-7.701653000	0.350280000	-0.533381000
C	-2.377161000	0.763069000	0.494595000
C	-1.692819000	-0.661425000	0.516784000
C	-2.118357000	1.526834000	1.797095000

H	-1.045660000	1.694527000	1.921577000
H	-2.624071000	2.498229000	1.754416000
H	-2.490788000	0.982417000	2.667781000
C	-1.960275000	-1.430243000	-0.780536000
H	-1.454501000	-2.401646000	-0.738091000
H	-3.033905000	-1.597608000	-0.897624000
H	-1.593490000	-0.889115000	-1.655691000
C	-0.189726000	-0.541335000	0.786293000
C	0.315066000	-0.748018000	2.076608000
C	0.714652000	-0.229908000	-0.239705000
C	1.682978000	-0.644325000	2.338316000
H	-0.372528000	-0.992925000	2.879446000
C	2.081637000	-0.125299000	0.019418000
H	0.351605000	-0.059224000	-1.246939000
C	2.573297000	-0.330937000	1.310713000
H	2.050827000	-0.808721000	3.348004000
H	2.764344000	0.116185000	-0.791312000
H	3.638379000	-0.250989000	1.511373000
O	-1.723064000	1.452452000	-0.590459000
H	-2.003277000	2.384146000	-0.545367000
O	-2.339184000	-1.346478000	1.609436000
H	-2.092467000	-2.286105000	1.541398000

H_2

E= -1.179350823690 H

H	-2.560307000	-0.122873000	0.000000000
H	-3.304722000	-0.122873000	0.000000000

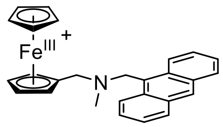

{Fc$^+$–N–an}

E= -2363.072349288148 H

C	7.859198000	0.757499000	21.519741000
H	7.223557000	-0.048701000	21.855336000
C	8.400385000	1.793410000	22.336937000
H	8.206273000	1.930584000	23.390983000
C	9.154642000	2.668324000	21.508871000
H	9.639137000	3.581981000	21.826849000
C	9.100491000	2.170145000	20.170792000
H	9.551758000	2.632856000	19.303483000
C	8.298993000	0.982373000	20.174545000
H	8.052868000	0.376718000	19.313024000
C	6.388135000	4.531387000	20.615522000
H	6.926070000	5.428746000	20.893418000
C	6.454063000	3.869061000	19.353276000
H	7.042809000	4.183734000	18.501991000
C	5.651251000	2.687287000	19.436887000
H	5.502425000	1.957623000	18.652455000
C	5.107968000	2.621084000	20.763002000
H	4.462770000	1.842599000	21.145466000
C	5.544545000	3.781506000	21.483738000
C	5.248927000	4.152427000	22.907018000
H	4.201801000	4.481174000	22.984443000
H	5.874220000	5.007969000	23.175955000
C	4.249686000	2.445039000	24.333625000
H	3.596582000	3.240252000	24.722715000
H	3.739680000	2.030136000	23.458948000
C	4.437796000	1.371873000	25.378004000
C	4.796015000	0.064811000	24.973085000
C	4.244060000	1.656051000	26.752556000
C	4.951811000	-0.981940000	25.958533000
C	5.013514000	-0.279747000	23.604495000
C	4.411344000	0.600870000	27.731437000
C	3.898687000	2.953941000	27.245197000
C	5.298523000	-2.300238000	25.534252000
C	4.757071000	-0.686485000	27.310712000
C	5.340889000	-1.561352000	23.233158000
H	4.931666000	0.489003000	22.846189000
C	4.223408000	0.887690000	29.117855000
C	3.732285000	3.191789000	28.587505000
H	3.768516000	3.774391000	26.549174000
C	5.485772000	-2.587466000	24.206183000
H	5.408300000	-3.071460000	26.292879000
H	4.876457000	-1.474148000	28.051703000
H	5.496360000	-1.795835000	22.183442000
H	4.351683000	0.076239000	29.830146000
C	3.893539000	2.149186000	29.540253000
H	3.472954000	4.190582000	28.927999000
H	5.746446000	-3.594079000	23.891554000
H	3.753653000	2.358595000	30.597054000
N	5.515815000	3.047627000	23.849568000
C	6.439966000	3.447711000	24.912745000
H	7.349220000	3.842298000	24.452371000
H	6.015354000	4.223028000	25.573408000
H	6.700049000	2.575828000	25.517915000
Fe	7.169808000	2.573526000	20.788107000

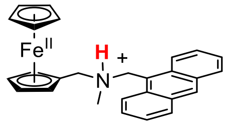

{Fc–NH$^+$–an}

E= -2363.716349167849 H

C	7.887289000	0.826631000	21.536874000
H	7.283986000	0.028499000	21.946292000
C	8.423814000	1.934920000	22.269143000
H	8.335519000	2.098520000	23.335353000
C	9.141331000	2.768708000	21.352172000
H	9.646036000	3.694743000	21.594858000
C	9.057255000	2.168996000	20.054224000
H	9.478656000	2.568906000	19.141244000
C	8.280688000	0.970189000	20.168396000
H	8.014016000	0.306024000	19.356683000
C	6.471185000	4.487561000	20.571904000

H	7.007406000	5.397115000	20.810374000
C	6.447470000	3.819770000	19.308518000
H	6.981440000	4.125730000	18.418521000
C	5.635187000	2.647931000	19.441503000
H	5.445509000	1.914240000	18.669031000
C	5.146195000	2.593935000	20.783944000
H	4.496103000	1.830833000	21.190366000
C	5.664987000	3.732683000	21.492207000
H	6.161534000	2.350759000	23.438727000
C	5.371140000	4.174650000	22.888124000
H	4.332179000	4.491472000	23.008457000
H	6.023576000	5.003845000	23.160182000
C	4.281050000	2.437398000	24.365839000
H	3.682486000	3.266193000	24.737970000
H	3.817558000	2.069217000	23.450966000
C	4.446135000	1.349395000	25.391072000
C	4.781824000	0.037927000	24.972799000
C	4.235422000	1.630128000	26.764579000
C	4.933035000	-1.009760000	25.958237000
C	4.977104000	-0.318757000	23.603410000
C	4.388983000	0.568184000	27.736955000
C	3.876732000	2.922244000	27.262281000
C	5.272954000	-2.330970000	25.537835000
C	4.737962000	-0.715543000	27.310165000
C	5.296805000	-1.602747000	23.235915000
H	4.876109000	0.432831000	22.829209000
C	4.184970000	0.842685000	29.123342000
C	3.687473000	3.146422000	28.603558000
H	3.751419000	3.751566000	26.576023000
C	5.451378000	-2.624852000	24.210951000
H	5.382011000	-3.098435000	26.300088000
H	4.855608000	-1.506086000	28.048207000
H	5.435205000	-1.841123000	22.185000000
H	4.307591000	0.025323000	29.829548000
C	3.843596000	2.098634000	29.551161000
H	3.414531000	4.140416000	28.946974000
H	5.707197000	-3.633079000	23.898190000
H	3.689893000	2.299940000	30.607433000
N	5.604788000	3.072056000	23.922299000
C	6.426270000	3.577016000	25.070412000
H	7.364477000	3.953255000	24.663312000
H	5.871621000	4.375945000	25.561265000
H	6.612199000	2.752202000	25.756635000
Fe	7.193816000	2.594417000	20.756374000

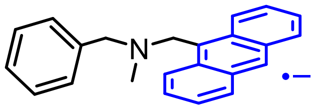

E= -945.022828071055 H

C	2.063075000	-3.088407000	0.460264000
C	3.379388000	-2.648086000	0.374851000
C	3.699853000	-1.272567000	0.219981000
C	2.623904000	-0.303822000	0.142423000
C	1.296047000	-0.795512000	0.244157000
C	1.017411000	-2.153942000	0.396566000
C	5.038335000	-0.828540000	0.147296000
C	2.955226000	1.079880000	-0.027403000
C	4.318524000	1.516256000	-0.095087000
C	5.378021000	0.533026000	-0.000342000
C	6.728988000	0.968613000	-0.062989000
H	7.514127000	0.217592000	0.007080000
C	7.058214000	2.312820000	-0.211504000
C	6.037268000	3.269989000	-0.305320000
C	4.697858000	2.876847000	-0.245831000
H	5.838318000	-1.565180000	0.211561000
H	1.846461000	-4.147250000	0.579998000
H	4.199690000	-3.362036000	0.428527000
H	0.461288000	-0.101995000	0.207836000
H	-0.016128000	-2.483990000	0.469773000
H	8.101028000	2.618147000	-0.254185000
H	6.282587000	4.322611000	-0.423743000
H	3.937569000	3.648790000	-0.336767000
C	1.890350000	2.111379000	-0.124783000
H	2.112497000	2.877511000	-0.872016000
H	0.897111000	1.710741000	-0.324052000
H	2.686667000	3.215814000	1.453704000
C	0.899094000	4.159570000	0.987859000
H	-0.107168000	3.811073000	0.749104000
H	1.331429000	4.657231000	0.117201000
C	0.912709000	5.051322000	2.197447000
C	2.048424000	5.818277000	2.494052000
C	-0.198705000	5.117512000	3.046305000
C	2.073943000	6.630942000	3.626423000
H	2.911836000	5.777261000	1.833536000
C	-0.176362000	5.936600000	4.175805000
H	-1.081519000	4.525859000	2.817512000
C	0.960870000	6.692048000	4.469304000
H	2.958316000	7.221891000	3.847985000
H	-1.045175000	5.983886000	4.826457000
H	0.979043000	7.329529000	5.348913000
C	1.227788000	2.051217000	2.308110000
H	0.205465000	1.759379000	2.067102000
H	1.873828000	1.177033000	2.379757000
H	1.257156000	2.622314000	3.235553000
N	1.738866000	2.909994000	1.197085000

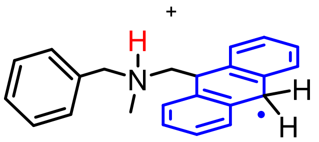

E= -945.46823919 H

C	2.064625000	-3.061603000	0.548198000
C	3.381782000	-2.626981000	0.406218000
C	3.689071000	-1.280343000	0.202735000
C	2.643477000	-0.314838000	0.140623000
C	1.308201000	-0.782965000	0.285494000

C	1.024919000	-2.125035000	0.483635000
C	2.958327000	1.080413000	-0.042304000
C	4.326270000	1.526275000	-0.162709000
C	5.396110000	0.590470000	-0.106032000
C	6.715565000	1.035251000	-0.207232000
H	7.518930000	0.303180000	-0.164528000
C	7.018979000	2.387490000	-0.362621000
C	5.977008000	3.321308000	-0.422576000
C	4.659250000	2.900408000	-0.325325000
H	1.849313000	-4.114097000	0.707479000
H	4.195740000	-3.346953000	0.454795000
H	0.480350000	-0.084723000	0.249184000
H	-0.007471000	-2.445197000	0.592039000
H	8.052958000	2.710770000	-0.438550000
H	6.195438000	4.377979000	-0.547462000
H	3.879272000	3.653777000	-0.386129000
C	1.873755000	2.112679000	-0.090427000
H	2.071083000	2.864519000	-0.855568000
H	0.883100000	1.701553000	-0.271056000
H	2.696532000	3.203822000	1.491717000
C	0.908467000	4.150903000	1.021233000
H	-0.094273000	3.800440000	0.772881000
H	1.347443000	4.652891000	0.157140000
C	0.916950000	5.038110000	2.235387000
C	2.046438000	5.815648000	2.526505000
C	-0.189925000	5.087734000	3.091118000
C	2.070082000	6.626465000	3.660998000
H	2.905522000	5.786870000	1.860209000
C	-0.169369000	5.904372000	4.223017000
H	-1.066817000	4.486287000	2.866891000
C	0.961695000	6.671428000	4.511221000
H	2.948795000	7.226901000	3.878307000
H	-1.033803000	5.939847000	4.879782000
H	0.978513000	7.306281000	5.392587000
C	1.239174000	2.036041000	2.348305000
H	0.220982000	1.734819000	2.103990000
H	1.890238000	1.168264000	2.440119000
H	1.259860000	2.619041000	3.267855000
N	1.752393000	2.893805000	1.229896000
C	5.132405000	-0.883409000	0.044734000
H	5.708021000	-1.267854000	0.898388000
H	5.548274000	-1.407358000	-0.829134000

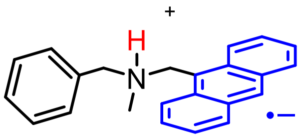

E= -944.98723988 H

C	2.117494000	-3.113370000	0.496709000
C	3.423920000	-2.651214000	0.375113000
C	3.717706000	-1.270573000	0.205784000
C	2.623641000	-0.319846000	0.149916000
C	1.306744000	-0.834589000	0.287406000
C	1.054650000	-2.196907000	0.453780000
C	5.047172000	-0.804747000	0.095903000
C	2.928030000	1.070040000	-0.031919000
C	4.283505000	1.528193000	-0.136869000
C	5.361197000	0.561992000	-0.065972000
C	6.703680000	1.019109000	-0.167219000
H	7.502774000	0.281622000	-0.116055000
C	7.007963000	2.368190000	-0.328185000
C	5.969895000	3.309509000	-0.395216000
C	4.638411000	2.895425000	-0.297384000
H	5.860533000	-1.527683000	0.142696000
H	1.922246000	-4.174907000	0.627621000
H	4.256375000	-3.351834000	0.412022000
H	0.459581000	-0.155908000	0.270115000
H	0.029025000	-2.543519000	0.554810000
H	8.044313000	2.688943000	-0.401185000
H	6.195191000	4.365565000	-0.521440000
H	3.864556000	3.657100000	-0.361780000
C	1.845659000	2.087719000	-0.102331000
H	2.036423000	2.850658000	-0.861098000
H	0.851605000	1.676044000	-0.272395000
H	2.671230000	3.181501000	1.474643000
C	0.899737000	4.160813000	1.015222000
H	-0.111974000	3.831083000	0.773555000
H	1.341369000	4.651953000	0.145746000
C	0.930260000	5.053472000	2.224865000
C	2.069796000	5.822768000	2.501243000
C	-0.163288000	5.111990000	3.097786000
C	2.115777000	6.632531000	3.635970000
H	2.919504000	5.787001000	1.823343000
C	-0.120426000	5.927405000	4.229891000
H	-1.047958000	4.517129000	2.886479000
C	1.020432000	6.686049000	4.502725000
H	3.002512000	7.225632000	3.841037000
H	-0.975098000	5.968525000	4.899183000
H	1.055129000	7.319947000	5.384298000
C	1.197679000	2.041967000	2.337937000
H	0.166501000	1.774644000	2.107149000
H	1.822238000	1.151795000	2.401769000
H	1.249766000	2.607698000	3.267514000
N	1.718040000	2.892713000	1.222325000

S17. References

1. Suryanto, B. H. R.; Matuszek, K.; Choi, J.; Hodgetts, R. Y.; Du, H.-L.; Bakker, J. M.; Kang, C. S. M.; Cherepanov, P. V.; Simonov, A. N.; MacFarlane, D. R. Nitrogen reduction to ammonia at high efficiency and rates based on a phosphonium proton shuttle. *Science* **2021**, *372* (6547), 1187–1191.
2. Garrido-Barros, P.; Derosa, J.; Chalkley, M. J.; Peters, J. C. Tandem electrocatalytic N2 fixation via proton-coupled electron transfer. *Nature* **2022**, *609* (7925), 71–76.
3. Gaussian 09, Revision D.01, M. J. Frisch; G. W. Trucks, H. B. Schlegel, G. E. Scuseria, M. A. Robb, J. R. Cheeseman, G. Scalmani, V. Barone, B. Mennucci, G. A. Petersson, H. Nakatsuji, M. Caricato, X. Li, H. P. Hratchian, A. F. Izmaylov, J. Bloino, G. Zheng, J. L. Sonnenberg, M. Hada, M. Ehara, K. Toyota, R. Fukuda, J. Hasegawa, M. Ishida, T. Nakajima, Y. Honda, O. Kitao, H. Nakai, T. Vreven, J. A. Montgomery, Jr., J. E. Peralta, F. Ogliaro, M. Bearpark, J. J. Heyd, E. Brothers, K. N. Kudin, V. N. Staroverov, R. Kobayashi, J. Normand, K. Raghavachari, A. Rendell, J. C. Burant, S. S. Iyengar, J. Tomasi, M. Cossi, N. Rega, J. M. Millam, M. Klene, J. E. Knox, J. B. Cross, V. Bakken, C. Adamo, J. Jaramillo, R. Gomperts, R. E. Stratmann, O. Yazyev, A. J. Austin, R. Cammi, C. Pomelli, J. W. Ochterski, R. L. Martin, K. Morokuma, V. G. Zakrzewski, G. A. Voth, P. Salvador, J. J. Dannenberg, S. Dapprich, A. D. Daniels, Ö. Farkas, J. B. Foresman, J. V. Ortiz, J. Cioslowski, and D. J. Fox, Gaussian, Inc., Wallingford CT, 2009.
4. Tao, J.; Perdew, J. P.; Staroverov, V. N.; Scuseria, G. E. Climbing the Density Functional Ladder: Nonempirical Meta--Generalized Gradient Approximation Designed for Molecules and Solids. *Phys. Rev. Lett.* **2003**, *91* (14), 146401.
5. Weigend, F. Accurate Coulomb-fitting basis sets for H to Rn. *Phys. Chem. Chem. Phys.* **2006**, *8* (9), 1057–1065.
6. Weigend, F.; Ahlrichs, R. Balanced basis sets of split valence, triple zeta valence and quadruple zeta valence quality for H to Rn: Design and assessment of accuracy. *Phys. Chem. Chem. Phys.* **2005**, *7* (18), 3297–3305.
7. Grimme, S.; Antony, J.; Ehrlich, S.; Krieg, H. A consistent and accurate ab initio parametrization of density functional dispersion correction (DFT-D) for the 94 elements H-Pu. *The Journal of Chemical Physics* **2010**, *132* (15), 154104.
8. Marenich, A. V.; Cramer, C. J.; Truhlar, D. G. Universal Solvation Model Based on Solute Electron Density and on a Continuum Model of the Solvent Defined by the Bulk Dielectric Constant and Atomic Surface Tensions. *J. Phys. Chem. B* **2009**, *113* (18), 6378–6396.
9. Kaljurand, I.; Kütt, A.; Sooväli, L.; Rodima, T.; Mäemets, V.; Leito, I.; Koppel, I. A. Extension of the Self-Consistent Spectrophotometric Basicity Scale in Acetonitrile to a Full Span of 28 pKa Units: Unification of Different Basicity Scales. *J. Org. Chem.* **2005**, *70* (3), 1019–1028.
10. Farrugia, T. J.; Magri, D. C. ‘Pourbaix sensors’: a new class of fluorescent pE–pH molecular AND logic gates based on photoinduced electron transfer. *New J. Chem.* **2012**, *37* (1), 148–151.
11. Pickett, C. J.; Talarmin, J. Electrosynthesis of ammonia. *Nature* **1985**, *317* (6038), 652–653.

12. Derosa, J.; Garrido-Barros, P.; Peters, J. C. Electrocatalytic Reduction of C–C π-Bonds via a Cobaltocene-Derived Concerted Proton–Electron Transfer Mediator: Fumarate Hydrogenation as a Model Study. *J. Am. Chem. Soc.* **2021**, *143* (25), 9303–9307.
13. Chapin, B. M.; Metola, P.; Vankayala, S. L.; Woodcock, H. L.; Mooibroek, T. J.; Lynch, V. M.; Larkin, J. D.; Anslyn, E. V. Disaggregation is a Mechanism for Emission Turn-On of ortho-Aminomethylphenylboronic Acid-Based Saccharide Sensors. *J. Am. Chem. Soc.* **2017**, *139* (15), 5568–5578.
14. Busch, Ed., D. H. *"The Synthesis of Molybdenum and Tungsten Dinitrogen Complexes" in Inorganic Syntheses*; John Wiley & Sons, 1980; Vol. XX.
15. Raamat, E.; Kaupmees, K.; Ovsjannikov, G.; Trummal, A.; Kütt, A.; Saame, J.; Koppel, I.; Kaljurand, I.; Lipping, L.; Rodima, T.; Pihl, V.; Koppel, I. A.; Leito, I. Acidities of strong neutral Brønsted acids in different media. *Journal of Physical Organic Chemistry* **2013**, *26* (2), 162–170.
16. Xu, K.; Zhao, J.; Escudero, D.; Mahmood, Z.; Jacquemin, D. Controlling Triplet–Triplet Annihilation Upconversion by Tuning the PET in Aminomethyleneanthracene Derivatives. *J. Phys. Chem. C* **2015**, *119* (42), 23801–23812.
17. Rabani, J.; Mamane, H.; Pousty, D.; Bolton, J. R. Practical Chemical Actinometry—A Review. *Photochemistry and Photobiology* **2021**, *97* (5), 873–902.
18. Bank, S.; Bockrath, B. Reactions of aromatic radical anions. VII. Kinetic study of the reaction of sodium anthracene with water. *J. Am. Chem. Soc.* **1972**, *94* (17), 6076–6083.
19. Donovan-Mtunzi, S.; Richards, R. L.; Mason, J. Nitrogen-15 nuclear magnetic resonance spectroscopy of hydrazido(2–)-, imido-, and nitrido-complexes of molybdenum and tungsten. *J. Chem. Soc., Dalton Trans.* **1984**, No. 7, 1329–1332.
20. Chatt, J.; Pearman, A. J.; Richards, R. L. Conversion of dinitrogen in its molybdenum and tungsten complexes into ammonia and possible relevance to the nitrogenase reaction. *J. Chem. Soc., Dalton Trans.* **1977**, No. 19, 1852–1860.
21. BDFEs were calibrated to our previously reported cobaltocene PCET mediator system (*Science* 2020, 369, 850-854, main text reference 20), wherein the reduction potential and pK_a were experimentally determined. See: Libot, C.; Pletcher, D. The reduction of carbonyl compounds at carbon electrodes in acidic water/methanol mixtures. *Electrochemistry Communications* **2000**, *2* (3), 141–144 for the experimentally determined pK_a and reduction potential of acetophenone.
